# Genomic Typing of Meningococcal Carriage Isolates in an Urban Sexual Health Clinic

**DOI:** 10.3390/pathogens15050516

**Published:** 2026-05-12

**Authors:** Yih-Ling Tzeng, Soma Sannigrahi, Abigail Norris Turner, Alexandria M. Carter, Brandon Snyder, Jose A. Bazan, David S. Stephens

**Affiliations:** 1 Division of Infectious Diseases, Department of Medicine, Emory University School of Medicine, Atlanta, GA 30322, USA; 2 Division of Infectious Diseases, Department of Internal Medicine, The Ohio State University College of Medicine, Columbus, OH 43210, USA; 3 Sexual Health Clinic, Columbus Public Health, Columbus, OH 43215, USA; 4 Division of Epidemiology, The Ohio State University College of Public Health, Columbus, OH 43210, USA

**Keywords:** *Neisseria meningitidis*, carriage, capsule, clonal complex, ST-1466, STI clinics

## Abstract

Asymptomatic pharyngeal *Neisseria meningitidis* (*Nm*) carriage is seen in >30% of sexually transmitted infection (STI) clinic attendees. With increasing reports of *Nm* urethritis and STI-related invasive disease outbreaks, longitudinal assessment and genomic characterization of *Nm* among patients at an urban STI clinic population in Columbus, Ohio, was undertaken. This study determined the genomic basis of oropharyngeal, urogenital and rectal *Nm* isolates carried by patients presenting for care. Cultures using media selective for *Neisseria* spp., *Nm*-specific PCR screening of colonies with oxidase-positive Gram-negative diplococci, PCR-based genogrouping and whole-genome sequencing of confirmed *Nm* were performed. Overall, genomic data of 453 oropharyngeal, 10 urethral, 5 rectal and 1 cervical *Nm* isolate were obtained between January 2018 and December 2019. Among oropharyngeal *Nm* isolates, genogrouping identified 37.7% as *cnl* (capsule null locus), 28% B, 13.5% E, 10.8% Z, 2.6% C and 2.6% Y. However, the *cps* locus was inactivated in ≥80% of isolates with specified genogroups. The major clonal complexes (ccs) were cc53, cc32, cc41/44, cc1157, cc198 and cc4821. Two oropharyngeal, one rectal and three urethral isolates belonged to the ST-11 *Nm* urethritis clade (*Nm*UC). Group Y ST-1466 *Nm*, recently linked to global urogenital and systemic infections, was also identified in the oropharynx.

## 1. Introduction

*Neisseria meningitidis* (*Nm*) remains a leading worldwide cause of bacterial meningitis and rapid fatal sepsis in children, young adults and otherwise healthy individuals [1]. Usually, *Nm* inhabits the human respiratory tract of 3–20% healthy individuals in the absence of outbreaks or crowding [2,3] and is transmitted from person to person by close contact of large, aerosolized droplets or via contact with oral or nasal secretions. Invasive meningococcal disease (IMD), which occurs 3–10 days after *Nm* acquisition, is caused by isolates expressing six of twelve capsular polysaccharides (CPSs), A, B, C, W, Y and X, classified based on CPS structural and genetic differences [4]. Factors influencing the establishment of carriage versus the development of IMD include host susceptibility, expression of capsule, pili and other additional virulence determinants [2,5,6,7,8]. In most instances, the acquisition of *Nm* in the upper respiratory tract does not result in IMD, which has a mortality rate that remains at 10–15% even with effective antibiotic therapy and supportive care. Meningococcal conjugated polysaccharide–protein vaccines for groups A, C, Y, X and W, and outer membrane protein vaccines targeting group B meningococcus provide protection against IMD; and the meningococcal polysaccharide–protein conjugate vaccines also interfere with transmission (herd protection).

There is growing concern about *Nm* as a sexually transmitted pathogen. Nasopharyngeal *Nm* colonization is highest among young adults and in men who have sex with men (MSM). Several *Nm* carriage studies have reported rates > 30% from STI clinics, almost exclusively focusing on MSM populations [9,10,11,12]. In the last twenty-five years, multiple outbreaks of IMD have occurred in MSM populations [13,14,15].

Starting in 2015, clusters of *Nm* urethritis cases resembling gonorrhea were reported in multiple US states and, subsequently, globally [16,17,18,19,20,21,22]. These cases were caused by a molecularly linked clade of non-groupable meningococci within the hypervirulent cc11 (the “US_*Nm*NG urethritis clade”; *Nm*UC) [23]. Recently, *Nm* isolates belonging to a group Y, ST-1466/cc174 cluster were identified globally as a cause of meningococcal urethritis and, separately, as a cause of IMD, especially in the US [24]. These events indicate the spectrum of *Nm* as a sexually transmitted pathogen. *Nm* transmission to the genital tract most likely occurs through oral sex with a partner who carries *Nm* in the nasopharynx. To better characterize the *Nm*UC carriage among the STI patients in the first large *Nm*UC outbreak reported in Columbus Ohio [16], we collected specimens from the oropharynx, urethra, cervix, and rectum regardless of symptoms and screened for *Nm* colonization. All the recovered *Nm* isolates were then characterized by whole-genome sequencing (WGS) analyses. The goal of this work was to capture circulating *Nm* including *Nm*UC isolates at all probable mucosal sites among the clinic’s population. Here, we report on the genomic characterization, including clonal complex, *cps* locus assessment and predicted meningococcal vaccine coverage of *Nm* isolates recovered from an urban STI clinic population.

## 2. Materials and Methods

### 2.1. Laboratory Procedures

During the study period, the STI clinic in Columbus was participating in the U.S. CDC’s Enhanced Gonococcal Isolate Surveillance Program (eGISP) that was established in 2017. This national (passive) surveillance program evaluated *Neisseria gonorrhoeae* (*Ng*) antimicrobial susceptibility as well as the burden of urogenital infections due to *Nm* through surveillance of both urethral and non-urethral sites (including oropharyngeal, cervical, and rectal). During the study period, all individuals who presented for STI screening were eligible for *Ng* and *Nm* culture sample collection based on the clinic’s STI screening protocol. Routinely, all patients who presented for care to the STI clinic were screened for *Ng* and *Chlamydia trachomatis* (*Ct*) from genital sites and from extragenital sites if site-specific exposure (oral sex and/or receptive anal sex) was reported in the last 12 months. Genital screening was carried out through NAAT (APTIMA Combo2 Assay, Hologic, Inc., Marlborough, MA, USA) from urine, while extragenital screening was carried out through oropharyngeal and rectal swabs for NAAT. Due to the STI clinic’s participation in eGISP during the study period, patients also had *Ng* screening with genital and extragenital swabs for culture using modified Thayer–Martin (MTM) media (Hardy Diagnostics, Santa Maria, CA, USA). All oropharyngeal, cervical, and rectal swabs were inoculated onto MTM media, while only urethral swabs with exudate that showed Gram-negative intracellular diplococci on Gram stain were inoculated onto MTM media. API NH (bioMérieux, Marcy l’Etoile, France) was performed on colonies with oxidase-positive Gram-negative diplococci to distinguish between *Ng* and *Nm*. All isolates were tested for the presence of *sodC* [25] and *porA* for confirmation of *Nm*. All recovered *Nm* carriage isolates were then examined by whole-genome sequencing (below). Initial *Nm* serogroup identification by conventional PCR with primer pairs specific to *Nm* serogroups B, C, E, W, X, Y, Z and capsule null locus (*cnl*) (Table S1) was also performed. When indicated, patients received treatment consistent with CDC guidelines. The study was approved by the Ohio State University Institutional Review Board.

### 2.2. Whole-Genome Sequencing (WGS) and Phylogenetic Analyses

Next-generation sequencing services were provided by the Yerkes NHP Genomics Core at Emory University and The Microbial Genome Sequencing Center (MiGS) facility. The nucleotide sequence reads were assembled with the SPAdes (version 3.7) algorithm [26] provided at the Bacterial and Viral Bioinformatics Resource Center (BV-BRC). The contig assembly files were uploaded to PubMLST and analyzed by the embedded tool for genogroup [27], sequence type and clonal complex determinations [27,28]. The capsule genogroup was determined based on identification of at least one capsule group-specific gene in the genome. If the capsule genes identified were shared across multiple serogroups, but no group-specific genes were identified, the genogroup was reported as “unknown”. Capsule expression was predicted by genetic variations known to impact function of capsule genes, such as premature internal stops, alleles in the phase-variable “off” state, and gene deletions/insertions by an IS element. All genome data are deposited into the publicly available PubMLST database (https://pubmlst.org/bigsdb?db=pubmlst_neisseria_isolates). The PubMLST ID number of each isolate is listed in the Supplemental Materials Table S2. The contig statistic summary of all genomes, which include the total length, contig numbers, the maximal, minimal and mean contig lengths, N50, L50, N90, L90, N95 and L95, are listed in Table S4.

The predicted vaccine coverage analyses, including both 4CMenB (Bexsero) [29,30] and MenB-fHBP (Trumenba) [31,32], are based on the Meningococcal Deduced Vaccine Antigen Reactivity (MenDeVAR) Index [33] incorporated into the PubMLST database. MenDeVAR categorized isolates into 4 groups: isolates with an “exact match” contain ≥1 exact sequence match to antigenic variants found in the vaccine. A “cross-reactive” isolate contains ≥1 antigenic variant deemed cross-reactive to vaccine variants through experimental studies. The “none” labeling indicates that all the isolate’s antigenic variants have been deemed to not be cross-reactive to vaccine variants through experimental studies. The “insufficient data” designation means isolates that contain antigens for which there is insufficient data from or that are yet to be tested in experimental studies.

Genomic data for phylogenetic comparison were retrieved from the PubMLST *Neisseria* isolate database (https://pubmlst.org/neisseria/). Capsule genogroups were compiled from the ‘capsule group’ field in PubMLST and PCR amplification data. A minimum-spanning tree was generated by the PubMLST embedded plugin, GrapeTree, using *N. meningitidis* cgMLST v3 scheme and the default MSTreeV2 algorithm and visualizers within GrapeTree [34].

## 3. Results

### 3.1. Identification of Neisseria meningitidis Carriage in an Urban STI Clinic

Per clinic procedures, all patients presenting for care to the STI clinic were screened for *Neisseria gonorrhoeae* (*Ng*) and *Chlamydia trachomatis* (*Ct*) using nucleic acid amplification testing (NAAT) from genital sites and from extragenital sites if exposure was reported in the last year. During the study period, the STI clinic participated in the US Centers for Disease Control and Prevention’s (CDC) Enhanced Gonococcal Isolate Surveillance Project (eGISP), so patients also had genital and extragenital *Ng* screening with culture using media selective for *Neisseria* spp; Analytical Profile Index *Neisseria*-*Haemophilus* (API NH) was performed on oxidase-positive colonies with Gram-negative diplococci to distinguish between *Ng* and *Nm*. The *Nm* cultures were then processed for chromosomal DNA preparations and conventional PCR amplifications using primers specific to *Nm* (*sodC* and *porA*) [25,35].

In total, 453 oropharyngeal isolates, 10 urethral isolates, 5 rectal isolates and 1 cervical *Nm* isolate were recovered from the STI clinic patients (January 2018–December 2019) (Table 1 and Table S2). Most oropharyngeal *Nm* isolates (361/453, 79.7%) and all 5 rectal isolates were recovered from male patients, which likely reflects the male-dominant patient population. All *Nm* isolates were analyzed by whole-genome sequencing (WGS).

During the study, designed to better characterize *Nm* isolates in this population, an analysis of the population screened was performed on surveillance data captured through the eGISP; demographic data is only available for patients with oropharyngeal *Nm* carriage. The detailed analysis of the population screened was conducted between July 2018 and March 2019 [36]. During this time, oropharyngeal screening occurred at 5015 patient visits and *Nm* was detected at 163 of these visits (3.3%). The analysis confirmed that individuals with oropharyngeal *Nm* were primarily Caucasian (62.6%), with non-Hispanic ethnicity (98.8%). The median age was 27 years and 5.5% were living with HIV. Among male cases, 49.1% reported sex with men; among women, 98.0% reported sex with men. Meningococcal vaccination status was unknown for 71.2%, but 26.4% had documentation of prior MenACWY polysaccharide–protein conjugate vaccination and 2.3% had prior 4CMenB vaccination.

### 3.2. Clonal Complexes

**Figure 1:**
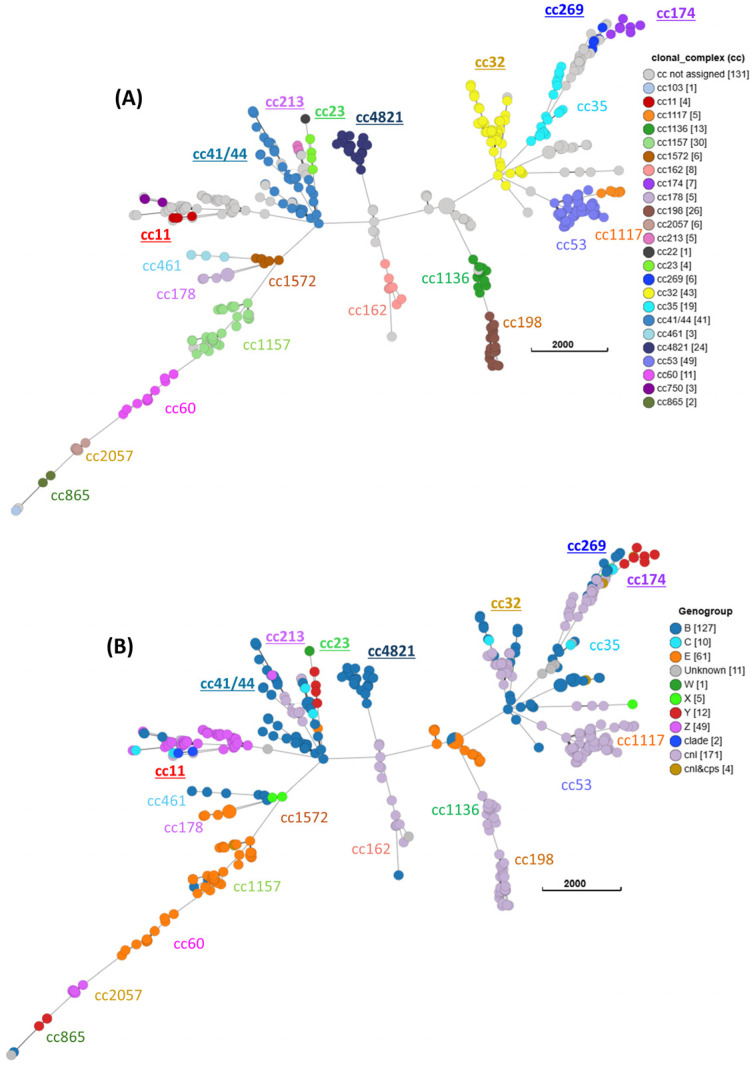
A minimal spanning tree of 453 oropharyngeal isolates recovered from Columbus, Ohio, STI clinic patient population. The number of isolates belonging to each clonal complex (**A**) or capsule group (**B**) is shown in square brackets in the index. Isolates without an assigned cc are shown in gray. Nine hyperinvasive clonal complexes are underlined. ST-1466 isolates belong to cc174. The “clade” labeling indicates isolates belonging to *Nm*UC [2,23]. The genogroup prediction was based on the genomic and PCR analyses and was not defined for 11 isolates. The tree was constructed by the Grape Tree tool embedded in PubMLST database and is based on the *N. meningitidis* core genome in the cgMLST v3 scheme.

Multi-locus sequence type (MLST) and clonal complex designation of all *Nm* isolates was achieved by WGS analyses embedded into PubMLST [37]. Of 453 oropharyngeal *Nm*, a total of 322 isolates (71%) were assigned to 24 clonal complexes (ccs), 117 *Nm* had an assigned MLST but did not belong to a cc, and no MLST was assigned to 14 isolates. Table 2 lists the distribution of ccs with associated capsule genogroups. The most common clonal complexes (>5%) were cc53 (*n* = 49, 10.8%), cc32 (*n* = 43, 9.5%), cc41/44 (*n* = 41, 9.1%), cc1157 (*n* = 30, 6.6%), cc198 (*n* = 26, 5.7%), and cc4821 (*n* = 24, 5.3%). Several ccs (cc11, cc23, cc32, cc41/44, cc174, cc213, cc269, and cc4821) have been described as hyperinvasive by various reports [7,8,11,38] (highlighted in gray in Table 2). We identified 30.2% (137/453) of the oropharyngeal isolates as belonging to a known hyperinvasive lineage; however, only 28.5% of these (39/137) appeared genetically capable of expressing capsules (see below). Using the Grape Tree tool based on the core genome [34], a minimal spanning tree was constructed for the oropharyngeal collection (Figure 1). As expected, isolates belonging to the same cc closely clustered together (Figure 1A), while the capsule genogroups were dispersed among various clonal complexes (Figure 1B). The five rectal *Nm* isolates were cc11 (*Nm*UC), cc41/44, cc4821 and two without a cc assignment. Three urethral *Nm* were *Nm*UC, 2 were cc41/44, one each of cc32, cc198 and cc1157 and two without a cc designation. The single cervical *Nm* isolate belonged to cc32. While phenotypic antibiotic resistance analyses were only performed for *Nm*UC isolates in a previous study [39] and not performed on *Nm* carriage isolates. Polymorphism analyses of ciprofloxacin resistance-conferring changes in *gyrA* and *parC* identified six oropharyngeal *cnl Nm* isolates (four ST-11563 and two ST-16088, no assigned cc) carrying both the GyrA/T91I and the ParC/S87R mutations that likely confer ciprofloxacin resistance. One urethral group B cc41/44 *Nm* isolate has a GyrA/T91I mutation.

### 3.3. Capsule Genogroup Distribution

**Figure 2:**
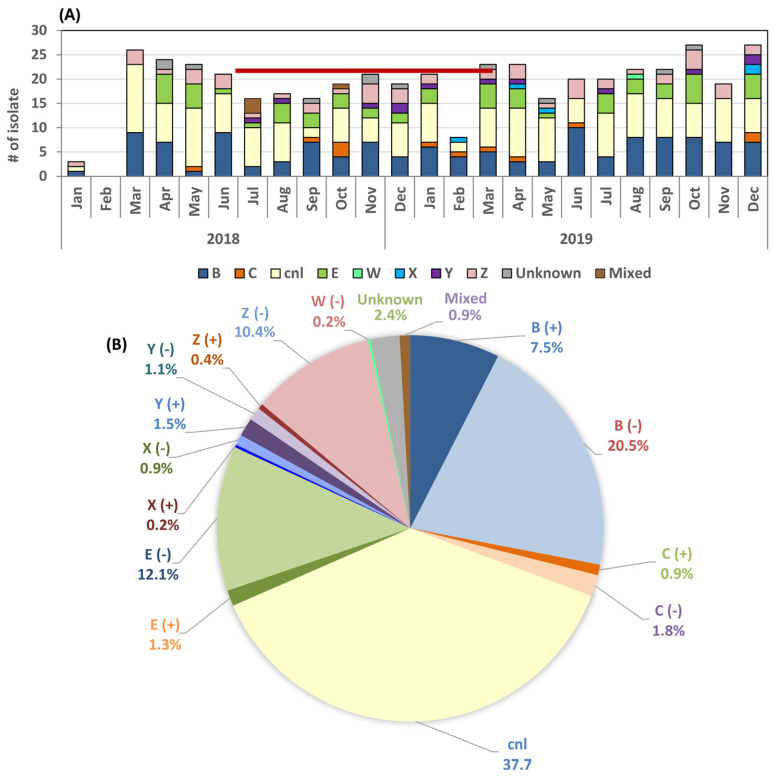
(**A**) Monthly recovery of isolates, 2018–2019. Capsule genogroup was predicted based on genome and PCR analyses. The red line marks the 9 months with detailed demographic information described in results. Few isolates were recovered in the beginning of the study (January and February 2018) due to the limited screening of patients. (**B**) The distribution of genogroups of the 453 oropharyngeal *Nm* isolates. (+) = intact *cps* locus, (−) = disrupted/inactivated *cps* locus.

The genogroup was defined by WGS using the PubMLST embedded tool and the traditional PCR screening of eight common capsule groups (Table 3) [40,41]. Among the 453 oropharyngeal isolates recovered between January 2018 and December 2019 (Figure 2A), the most common genogroup was *cnl* (capsule null locus) at 37.7%, followed by capsule group B (28.0%), E (13.5%), and Z (10.8%). Genogroups C, W, X, and Y (0.2–2.6%) were also detected. The capsule group was labeled unknown (*n* = 11, 2.4%) when these isolates contained portions of the shared *cps* genes, but serogroup-specific genes were not found and were negative on all PCR reactions (Table 3 and Figure 2B). There were four specimens (0.9%) showing positive PCR signals of both *cnl* and one capsule-specific gene, thus implying a mixed culture. The *cnl* genogroup was found mostly in cc35, cc53, cc162, cc198, cc1117 and cc1136, while many cc32 and cc41/44 isolates, which are typically group B, also carried the *cnl* locus. Group E capsule was found in cc60, cc178 and cc1157 (Table 2 and below). Group Y capsule was detected in cc174, cc23 and cc865, while group X was found in cc1572.

While the sequence-based analysis identified a group-specific backbone (e.g., capsule biosynthesis genes), many isolates were predicted to be inactivated in capsule production (Table 3). Using 127 group B genomes as an example, 9 had IS*1301* insertion in *cssA* or *csb*, 3 had IS*1655* in *ctrE*, 18 were phase-off in *csb* and 63 isolates were inactivated due to an internal stop in at least one gene. Thus, only 26.8% (34/127) of group B isolates has an intact genomic capsule locus. IS*1301* was also detected in group E (*cseC* or *cseE*, *n* = 3), group Y (*cssA*, *n* = 3) and group Z (*cszD*, *n* = 24) as well as 2 *Nm*UC isolates with IS*1301* insertion in *csc* [23]. A potentially intact capsule locus, that is, without any definitive genetic inactivating events, was only found in 54 genomes (34/127 B, 6/61 E, 1/5 X, 7/12 Y and 2/49 Z). Thus, ~12% of the *Nm* oropharyngeal carriage isolates (54/453) are expected to express capsular polysaccharide (Table 3). Among 10 urethral *Nm* isolates, 3 each were of group B and C (*Nm*UC), 2 were *cnl*, and one each was of group E and Z, all of which contained an inactivated *cps* locus. All rectal *Nm* isolates were also defective in capsule expression and belonged to groups B, C, E, Z and *cnl*. The single cervical *Nm* was *cnl*.

### 3.4. Capsule Genogroup E Carriage

Capsular genogroup E *Nm* (MenE) constituted 13.5% of all oropharyngeal isolates (61/453) collected in this study from STI clinic attendees, but only six isolates had an intact capsule (*cps*) locus by WGS. IMD due to group E *N. meningitidis* is rare and often associated with immunocompromised patients. One 2007 and two 2018 cases of IMD and one case of meningococcal conjunctivitis in previously healthy patients were reported to be caused by MenE in Queensland, Australia [42]. In both cases in 2018, the patients were found to have terminal complement (C7) deficiency. Molecular typing revealed the genotype of these Australia strains to be E:P1.21-7, 16:F5-36:ST-1157 (cc1157). Of the 30 cc1157 oropharyngeal *Nm* carriage isolates in our study collection, 13 were designated to be the same genotype as those Australian invasive isolates (two are ST-15073, a single-locus variant of ST-1157). Based on capsule genogroup, our cc1157 isolates were 28 E, 1 B and 1 mixed. However, based on WGS, all 30 cc1157 isolates were predicted to lack capsule expression due to either an internal stop in capsule genes or an IS*1301* insertion in the *cps* locus. Genogroup composition of *Nm* carriage isolates among students at three US universities shows group E to be the most common genogroup, representing 26% of these collections [43]. This differs from carriage evaluations conducted in university students in Europe and South America, which identified 2–9% group E isolates [43].

### 3.5. ST-1466/cc174 Carriage

**Figure 3:**
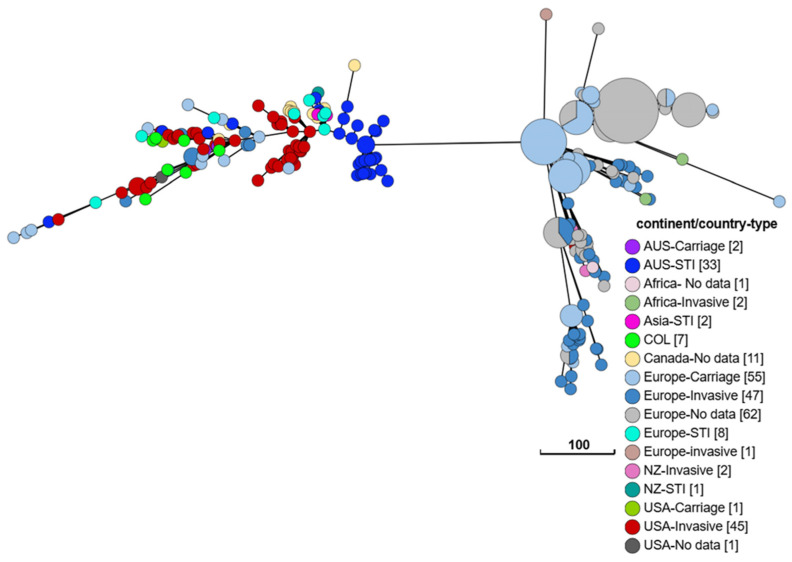
A minimal spanning tree of 281 ST-1466 (cc174) genomes curated from the PubMLST database. The geographic location and the disease status (invasive, STI or carriage) of isolates, if available, were used for grouping. The seven ST-1466 carriage isolates in our study (COL) are labeled in green. Isolates recorded with meningococcal STI in the “disease” category or labeled as urethral swab, rectal swab or female reproductive tract in the “source” category are classified as STI. Isolates lacking source information are shown in white. The number of isolates belonging to each group is shown as a square bracket in the index. The tree is constructed by the Grape Tree tool using the *N. meningitidis* cgMLST v3 scheme [34].

Seven ST-1466 carriage isolates (1.5%) were identified in our study population. ST-1466 isolates constitute the majority of cc174 and have been associated with the recent resurgence of IMD in the US, from 2022 to present [44,45], including a prolonged outbreak of group Y meningococcal disease in Virginia, US, during August 2022–March 2024 with a fatality rate of 19.4% [6]. These US ST-1466 invasive isolates share a fine-typing genetic profile: PorA (P1.21, 16), FetA (F3–7), and PorB (3–35). A 2024 CDC Health Alert Network (HAN) issued a health advisory on the increase in group Y IMD in the US, with 68% cases caused by ST-1466 isolates [24]. Importantly, an outbreak of urogenital infections in Australia in 2023 was caused by group Y/ST-1466 meningococci that were genetically most closely related to recent US IMD isolates [46] (Figure 3). ST-1466 has previously rarely been recovered in STI carriage studies.

All ST-1466 carriage isolates recovered in Columbus, Ohio, have an identical fine-typing profile to the recent US IMD isolates. Phylogenetic analyses of 281 ST-1466 genomes available in the PubMLST database (accessed on 15 December 2025) confirmed that seven Columbus Ohio ST-1466 carriage isolates (labeled as COL and colored in green, Figure 3) were very closely related to recent US ST-1466 invasive isolates (Figure 3). These ST-1466 carriage isolates and the US ST-1466 invasive isolates form a distinct branch separated from other historic ST-1466 isolates, mostly from Europe, by 196 alleles (i.e., branch length) based on core genome phylogenetic assessment. Interestingly, all recent ST-1466 urogenital STI isolates fall within this distinct branch regardless of the geographic locations that now include Australia, New Zealand, China, Spain and Scotland.

### 3.6. Nm cc4821 Carriage

Our study also identified 24 pharyngeal isolates (24/453, 5.3%) and 1 rectal cc4821 isolate in an urban STI clinic setting. While 60% of cc4821 PubMLST records are from China (583/971, 1972–2026), the recent geographic spread of cc4821 is evident based on increasing cc4821 records, now from many other countries, in the database (accessed on 5 February 2026). These cc4821 isolates are recovered from pharyngeal carriers, but many are also recovered from the rectum and urethra as well as from patients with IMD. The majority of cc4821 isolates (>60%) with capsule group assignment in the database were group B, followed by C, W, A, X, Y, K and *cnl*. The finding of cc4821 isolates expressing diverse capsule groups implies recombination events in the *cps* locus, resulting in capsule switching [47,48,49,50].

Ciprofloxacin resistance is frequently found within cc4821 [48,49,51]. However, all cc4821 carriage isolates identified in this study belong to capsule group B and are ciprofloxacin-susceptible based on polymorphism analyses of known resistance-conferring changes in *gyrA* and *parC*. A 2019 study by Lucidarme et al. examined 188 cc4821 records in PubMLST and identified four lineages [51]. In that collection, with one exception, isolates recovered outside of China exclusively belonged to the distinct RoW cluster within the lineage 2c [51]. A third of the RoW cluster isolates were from anogenital sites in men who have sex with men (MSM), and a few were from dual meningococcal/gonococcal coinfections [51]. The carriage isolates in this study grouped with North America isolates within lineage 2c in the phylogenetic analysis.

### 3.7. Meningococcal Vaccine Coverage

Based on the WGS analyses, only 54/453 (11.9%) of *Nm* isolates in this carriage study contained complete, uninterrupted coding sequences for the proteins required for capsule polysaccharide production and expression, and among C/W/Y genogroup isolates, only 11/25 (44%) were predicted to be encapsulated. Therefore, most of the *Nm* carriage isolates were un-encapsulated and would not be a target of capsular antibodies generated by meningococcal A/C/W/Y capsular polysaccharide–protein conjugate vaccines at mucosal surfaces. Two OMV/protein (4CMenB) or protein (bivalent MenB-FHbp)-based vaccines have been developed for the prevention of group B IMD. These two vaccines have different components, resulting in different levels of coverage based on the presence of vaccine antigen variants in a population, but neither vaccine significantly impacts pharyngeal meningococcal carriage [52,53] except possibly *cnl* cc53 with 4CMenB [53]. Genomic and experimental data from published sources have been used to develop and implement the MenDeVAR Index [33], which is available on PubMLST as “Bexsero reactivity” [29,30] and “Trumenba reactivity” [31,32], to categorize isolates into four groups. Among the 453 oropharyngeal isolates in this study, 68 (15%) have an “exact match”, 49 (10.8%) are “cross-reactive” and 2 are labeled “none”. The majority (334, 73.7%) are labeled “insufficient data” or are not yet tested in experimental studies. Based on the FHbp sequence variation (Trumenba reactivity) [31,32], 4 (0.9%) have an “exact match”, 255 (56.3%) are “cross-reactive” and 194 (42.8%) are labeled, “insufficient data”. The caveats of interpreting deduced coverage from sequence data are acknowledged [37].

## 4. Discussion

*Nm* carriage studies have been conducted in STI clinic settings, almost exclusively focusing on ‘men who have sex with men’ (MSM), but few report the genomic analysis of the *Nm* recovered. An oropharyngeal and rectal meningococcal carriage rate of 14.1% was found among healthy MSM in Rome, Italy, in 2016, of which cc162 and cc461, infrequent causes of IMD, were the most common *Nm* clonal complexes [9]. Group B was the most frequent capsular group identified, followed by Z, E and *cnl* [9]. Another study from Bologna, Italy, examined *Nm* carriage in a cohort of MSM, 2017–2019, and reported that 75.8% of pharyngeal specimens were positive for *sodC*, indicating a very high level of pharyngeal meningococcal carriage [54]. Non-groupable meningococci (referring to capsule groups other than A, B, C, W and Y or *cnl*) represented 71% of over four hundred pharyngeal carriage isolates followed by B (23.6%), C (2.1%), Y (1.8%) and W (1.1%) [54]. In Spain, a retrospective cross-sectional study of *Nm* carriage among MSM attending a sexual health unit, 2018–2021 [11], reported the oropharyngeal carriage rate was 29%. The most frequent groups were group B (40%) and non-groupable (45%). The most common clonal complexes were cc213 (6.6%) (also the most prevalent invasive cc in Spain), cc162 (6.6%), cc4821 (5.7%), cc53 (4.7%), cc1572 (3.8%), and cc11 (3.8%), with 20% of all isolates belonging to hyperinvasive lineages (cc11, cc4821, cc32, cc41/44, cc213, and cc269) [11]. Importantly, the definition of non-groupable varies among these studies, defined as either negative slide agglutination assay [10], negative rt-PCR detection of a disease-causing serogroup [54] or genome-based genogrouping as *cnl* or non-groupable [9,10].

In this US STI clinic population, a high percentage (~30%) of hyperinvasive lineages (Table 3), e.g., cc32 (9.5%), cc41/44 (9.1%), and cc4821 (5.3%), were found; cc213, cc162, and cc174 were found in <2%. A sexual health clinic in New York City reported oropharyngeal *Nm* carriage in MSM, 2016–2017, at 22.6%. Like our findings, WGS revealed that the *cps* locus of most NYC isolates (81.5%) was either *cnl* or incomplete, and therefore did not express capsules [10]. The most common NYC ccs detected were the hyperinvasive lineage cc4821 (14.3%, 24/168), followed by cc32, cc11, and cc41/44; of note, 4 of the 6 cc11 isolates were *Nm*UC [10].

In a different US population, cross-sectional meningococcal carriage evaluations were conducted at three US universities (2015–2016). The studies reported carriage rates ranging between 11 and 24% in college students and recovered 1514 *Nm* isolates with comprehensive WGS analyses on cc and capsule groups [43,55,56]. The most frequently observed clonal complexes in the university students were cc198 (*cnl*) (27.3%), followed by cc1157 (E) (17.4%), cc41/44 (9.8%), cc35 (7.4%), and cc32 (5.6%) [55]. While these ccs were also seen in our STI population, the percentage of isolates by cc differed: cc53 (10.8%), cc32 (9.5%), and cc4821 (5.3%) were distinctly higher in our STI population. The most common capsule genogroups in the university students, like in our study, were *cnl* (48.3%), group E (28.1%), and B (16.6%) [43], and many of these isolates (91.7%) were non-groupable phenotypically by Slide Agglutination Sero Grouping (SASG).

Detailed molecular surveillance of carriage and invasive isolates remains key in monitoring changes in the epidemiology and pathogenicity of meningococcal disease. Based on WGS analyses, only a small fraction of isolates (11.9%) in our carriage study contained complete, uninterrupted coding sequences for the genes required for capsule polysaccharide production and expression, a key determinant of invasive meningococcal disease. Thus, most *Nm* carriage isolates in the STI population were un-encapsulated, confirming other studies. Genotypical characterizations (WGS or PCR) of encapsulation status may overestimate the ability of isolates to express capsule [43]. It is possible that an even larger portion of the *Nm* carriage isolates are not expressing capsule. This is not surprising based on the selection of non-encapsulated variants due to local hypermutation and horizontal gene transfer at human pharyngeal mucosal surfaces [57,58] and the use of A/C/W/Y polysaccharide–protein conjugate vaccines in the US population that generate antibodies interfering with transmission and colonization of encapsulated *Nm* at mucosal surfaces. However, homologous recombination between meningococci can restore or switch to a functional *cps* locus. Also, encapsulated *Nm* of hyperinvasive lineages and clonal complexes associated with *Nm* urethritis outbreaks were recovered in our STI clinic population. Thus, vaccine prevention of IMD with both the polysaccharide–protein conjugates and group B-targeted protein vaccines remains important for individuals at risk in this population. *Nm* carriage studies provide insights into circulating capsule groups and clonal complexes and provide estimates of meningococcal vaccine coverage.

## Figures and Tables

**Table 1:** Distribution of *N. meningitidis* carriage isolates recovered in an STI clinic, Columbus, Ohio, 2018–2019.

Isolate	Total	469
**Gender**	Male	376
	Female	93
**Isolation site**	Oropharynx	453
	Urethra	10
	Cervix	1
	Rectum	5

**Table 2:** Clonal complex distribution of 453 oropharyngeal *N. meningitidis* *.

Clonal Complex (cc)	#	%	Capsule Genogroup
no assigned cc	131	28.9%	¥
cc53	49	10.8%	49 *cnl*
cc32	43	9.5%	19 B; 1 C; 23 *cnl*
cc41/44	41	9.1%	31 B; 1 C; 7 cnl; 1 E; 1 Z
cc1157	30	6.6%	28 E; 1 B; 1 mixed
cc198	26	5.7%	26 *cnl*
cc4821	24	5.3%	24 B
cc35	19	4.2%	5 B; 1 C; 7 cnl; 6 unknown
cc1136	13	2.9%	13 *cnl*
cc60	11	2.4%	11 E
cc162	8	1.8%	7 cnl; 1 unknown
cc174 (ST-1466)	7	1.5%	6 Y; 1 mixed
cc1572	6	1.3%	4 B; 2 X
cc2057	6	1.3%	6 Z
cc269	6	1.3%	3 B; 2 C; 1 *cnl*
cc1117	5	1.1%	5 *cnl*
cc178	5	1.1%	5 E
cc213	5	1.1%	3 B; 1 cnl; 1 unknown
cc11	4	0.9%	4 C
cc23	4	0.9%	4 Y
cc461	3	0.7%	3 B
cc750	3	0.7%	3 B
cc865	2	0.4%	2 Y
cc103	1	0.2%	1 unknown
cc22	1	0.2%	1 W

* The gray shading indicates hyperinvasive lineages. ¥ Includes 31 B; 3 C; 16 E; 3 X; 42 Z; 32 *cnl*; 2 mixed; 2 unknown.

**Table 3:** Capsule genogroup distribution based on WGS and PCR analyses.

Capsule Genogroup	Total (%)	Intact ^a^	Inactivated ^b^	IS1301 ^c^	IS1655 ^c^	Phase Off ^d^
**B**	127 (28.0)	34	63	9 (*cssA*, *csb*)	3 (*ctrE*)	18 (*csb*)
**C**	12 (2.6)	4	5	2 ^e^	1 (*ctrE*)	
**cnl**	171 (37.7)					
**E**	61 (13.5)	6	52	3 (*cseC*, *cseE*)		
**W**	1 (0.2)		1			
**X**	5 (1.1)	1	4			
**Y**	12 (2.6)	7	2	3 (*cssA*)		
**Z**	49 (10.8)	2	23	24 (*cszD*)		
**Unknown ^f^**	11 (2.4)					
**Mixed ^g^**	4 (0.9)					

^a.^ Includes isolates noted to have a *cps* locus and no defined capsule gene inactivation. ^b.^ Internal stop in at least one capsule gene. ^c.^ The capsule gene with an insertion is shown in parenthesis. ^d.^ The phase variable *csb* gene is phase-off and thus does not encode a complete coding sequence. ^e.^ IS1301 replacing *cssA/cssB/cssC* and a portion of *csc* (*Nm*UC). ^f.^ One or more capsule genes that are shared across multiple capsule groups are present, while no capsule group-specific gene is identified; thus, no genogroup can be defined. ^g.^ Both the *cnl* sequence and the capsule gene(s) are identified.

## Data Availability

The genome data of all *Nm* carriage isolates from this study had been made public accessible at the PubMLST database (https://pubmlst.org/bigsdb?db=pubmlst_neisseria_isolates).

## Supplementary Materials

The following supporting information can be downloaded at https://www.mdpi.com/article/10.3390/pathogens15050516/s1, Table S1: Primers used in the capsule PCR analysis; Table S2: Dataset of 469 *Nm* carriage isolates from STI clinics at Columbus, Ohio; Table S3: Dataset of ST-1466 isolates used in the phylogenic analysis; Table S4: Genomic contig statistical summary.

## Abbreviations

The following abbreviations are used in this manuscript:

*Nm*	*Neisseria meningitidis*
*Ng*	*Neisseria gonorrhoeae*
*Ct*	*Chlamydia trachomatis*
STI	Sexually transmitted infection
*Nm*UC	U.S. *Neisseria meningitidis* urethritis clade
cc	Clonal complex
WGS	Whole-genome sequencing
IMD	Invasive meningococcal disease
CPS	Capsular polysaccharide
*cps*	Capsule locus
MLST	Multi-locus sequence typing
*cnl*	Capsule null locus
eGISP	Enhanced Gonococcal Isolate Surveillance Program
