# Supplementary material for: Genomic Typing of Meningococcal Carriage Isolates in an Urban Sexual Health Clinic
Source: Pathogens. 2026 May 12;15(5):516. doi: 10.3390/pathogens15050516 (PMC13209751; doi:10.3390/pathogens15050516)
Supplement: Supplementary file 1 [file pathogens-15-00516-s001.zip › Table S1.pdf]

Table S1. Primer pairs used in the capsule PCR analysis

Serogroup	Primer name	Sequence (5'-3')	Product size
**E**	SgE-F2	ATTACGCTGACGGCATGTGGA	667
	ctrA-UR	TTGTCGCGGATTTGCAACTA	
**X**	SgX-F3	GTCTTTGTATAAGGCCCAAG	525
	ctrA-UR	TTGTCGCGGATTTGCAACTA	
**Z**	csz-F	TATGCGGTGCTGTTCGCTATG	667
	ctrA-UR	TTGTCGCGGATTTGCAACTA	
**B**	csb-F	TGCATGTCCCCTTTCCTGA	170
	csb-R	AATGGGGTAGCGTTGACTAACAA	
**C**	csc-F	GCACATTCAGGCGGGATTAG	442
	csc-R	TCTCTTGTTGGGCTGTATGGTGTA	
**W**	csw-F	GGTGTATGATATTCCAATCGTTGTA	300
	cswy-R	TTGGAATTCGTCCAATTCTTTTCG	
**Y**	csy-F	CATCTCAAAGCGAAGGCTTTGG	330
	cswy-R	TTGGAATTCGTCCAATTCTTTTCG	
**cnl**	cnl-HC344	GGATTGGACGAGCGAGAC	432
