# Supplementary material for: Genomic Typing of Meningococcal Carriage Isolates in an Urban Sexual Health Clinic
Source: Pathogens. 2026 May 12;15(5):516. doi: 10.3390/pathogens15050516 (PMC13209751; doi:10.3390/pathogens15050516)
Supplement: Supplementary file 1 [file pathogens-15-00516-s001.zip › Table S2.pdf]

Table S2. Dataset of 469 *Nm* carriage isolates from STI clinic at Columbus, Ohio

id	isolate	year	sex	source	capsule group	ST (MLST)	clonal_complex (MLST)	Bexsero_reactivity	fHbp peptide	NHBA peptide	NadA peptide	BAST (Bexsero Antigen Sequence Typing)	Trumenba reactivity	strain_designation	PorA_VR1	PorA_VR2	FetA_VR	gyrA	NEIS1525 parC
71954	COL201804-31	2018	male	throat swab	NG	2153	ST-162 complex	insufficient data	21	20	0		cross-reactive	NG: P1.18-1,30-5: F1-12: ST-2153 (cc162)	18-1	30-5	F1-12	4	36
72044	COL201804-48	2018	male	throat swab	E	15149	ST-60 complex	insufficient data	13	24	0	237	cross-reactive	E: P1.5,2: F5-13: ST-15149 (cc60)	5	2	F5-13	3	2231
72045	COL201904-29	2019	female	throat swab	cnl	34	ST-32 complex	exact match	1	20	1	3177	cross-reactive	cnl: P1.19,15: F5-186: ST-34 (cc32)	19	15	F5-186	2	51
72046	COL201904-31	2019	male	throat swab	cnl	823	ST-198 complex	cross-reactive	4	10	0		cross-reactive	cnl: P1.18-1,3-4: F-ND: ST-823 (cc198)	18-1	3-4		3	30
72047	COL201904-36	2019	female	throat swab	cnl	2578	ST-41/44 complex	exact match	100	2	0		insufficient data	cnl: P1.12-1,13-61: F1-5: ST-2578 (cc41/44)	12-1	13-61	F1-5	4	17
72048	COL201904-51	2019	female	throat swab	cnl	15162	ST-35 complex	insufficient data	24	21	0	4884	insufficient data	cnl: P1.7-2,13-66: F1-7: ST-15162 (cc35)	7-2	13-66	F1-7	4	29
72049	COL201904-84	2019	male	throat swab	cnl	11563		insufficient data	18	1511	0		insufficient data	cnl: P1.21-2,23-34: F3-8: ST-11563 ()	21-2	23-34	F3-8	361	243
72050	COL201904-87	2019	male	throat swab	X	15164	ST-1572 complex	insufficient data	260	20	0	3179	insufficient data	X: P1.7-12,14: F1-34: ST-15164 (cc1572)	7-12	14	F1-34	11	31
72051	COL201905-27	2019	female	throat swab	cnl	823	ST-198 complex	cross-reactive	4	10	0	741	cross-reactive	cnl: P1.18,25-15: F5-5: ST-823 (cc198)	18	25-15	F5-5	3	30
72052	COL201905-29	2019	male	throat swab	NG	3882		exact match	22	601	0		insufficient data	NG: P1.21-10,4: F5-7: ST-3882 ()	21-10	4	F5-7	11	309
72053	COL201905-30	2019	male	throat swab	cnl	823	ST-198 complex	cross-reactive	4	10	0		cross-reactive	cnl: P1.18,25-10: F5-5: ST-823 (cc198)	18	25-10	F5-5	3	30
72054	COL201905-35	2019	female	throat swab	cnl	823	ST-198 complex	cross-reactive	4	10	0		cross-reactive	cnl: P1.18-1,3-4: F-ND: ST-823 (cc198)	18-1	3-4		3	30
72055	COL201905-36	2019	female	throat swab	cnl	1136	ST-1136 complex	insufficient data	94	145	0	657	insufficient data	cnl: P1.18-4,25: F4-1: ST-1136 (cc1136)	18-4	25	F4-1	2	30
72057	COL201905-40	2019	male	throat swab	B	2840	ST-41/44 complex	insufficient data	19		0		cross-reactive	B: P1.18,25: F5-7: ST-2840 (cc41/44)	18	25	F5-7	2	8
72058	COL201905-41	2019	male	throat swab	E	178	ST-178 complex	insufficient data	13	6	0		cross-reactive	E: P1.19-5,15-23: F1-7: ST-178 (cc178)	19-5	15-23	F1-7	3	835
72060	COL201905-45	2019	male	throat swab	B	4221		insufficient data	106		0		insufficient data	B: P1.18,25: F1-62: ST-4221 ()	18	25	F1-62	12	29
72061	COL201905-47	2019	male	throat swab	cnl	34	ST-32 complex	exact match	1	20	1	3177	cross-reactive	cnl: P1.19,15: F5-1: ST-34 (cc32)	19	15	F5-1	2	51
72062	COL201905-48	2019	male	throat swab	cnl	53	ST-53 complex	insufficient data	102	58	0	651	insufficient data	cnl: P1.7,30-5: F1-2: ST-53 (cc53)	7	30-5	F1-2	28	13
72063	COL201905-50	2019	male	throat swab	B	6595	ST-4821 complex	insufficient data	16	234	0	3728	cross-reactive	B: P1.20,23: F3-36: ST-6595 (cc4821)	20	23	F3-36	12	275
72064	COL201905-51	2019	female	throat swab	X	2888		insufficient data	160	129	21	210	insufficient data	X: P1.19,15: F5-5: ST-2888 ()	19	15	F5-5	4	195
72065	COL201905-63	2019	male	throat swab	cnl	278	ST-35 complex	insufficient data	24	21	0	4884	insufficient data	cnl: P1.7-2,13-66: F1-7: ST-278 (cc35)	7-2	13-66	F1-7	4	29
72066	COL201905-64	2019	male	throat swab	Z	15143		insufficient data	22	329	0		insufficient data	Z: P1.5-1,10-85: F5-7: ST-15143 ()	5-1	10-85	F5-7	11	309
72067	COL201905-65	2019	female	throat swab	cnl	1136	ST-1136 complex	insufficient data	94	145	0		insufficient data	cnl: P1.18-4,25-26: F2-9: ST-1136 (cc1136)	18-4	25-26	F2-9	2	30
72068	COL201906-26	2019	male	throat swab	B	409	ST-41/44 complex	exact match	19	2	0	315	cross-reactive	B: P1.18-1,34: F1-5: ST-409 (cc41/44)	18-1	34	F1-5	2	156
72069	COL201906-27	2019	male	throat swab	cnl	1784	ST-32 complex	insufficient data	101	118			insufficient data	cnl: P1.18,25-18: F1-21: ST-1784 (cc32)	18	25-18	F1-21	2	2585
72070	COL201906-29	2019	male	throat swab	cnl	53	ST-53 complex	insufficient data	102	58	0	635	insufficient data	cnl: P1.7,30-3: F1-2: ST-53 (cc53)	7	30-3	F1-2	28	13
72071	COL201906-31	2019	male	urethral swab	B	897		insufficient data	13	8	0		cross-reactive	B: P1.12,16-75: F1-5: ST-897 ()	12	16-75	F1-5	12	704
72072	COL201906-34	2019	male	throat swab	Z	10866		insufficient data	16	601	0	871	cross-reactive	Z: P1.22,14-13: F5-7: ST-10866 ()	22	14-13	F5-7	11	314
72073	COL201906-37	2019	male	throat swab	cnl	53	ST-53 complex	insufficient data	102	58	0	635	insufficient data	cnl: P1.7,30-3: F1-2: ST-53 (cc53)	7	30-3	F1-2	28	13
72074	COL201906-39	2019	male	throat swab	B	15161		insufficient data	1284		0		insufficient data	B: P1.17,9-4: F1-7: ST-15161 ()	17	9-4	F1-7	2	8
72075	COL201906-40	2019	male	throat swab	B	10864	ST-269 complex	insufficient data	260	21	0		insufficient data	B: P1.19,15-1: F1-5: ST-10864 (cc269)	19	15-1	F1-5	2	311
72076	COL201906-41	2019	male	throat swab	cnl	53	ST-53 complex	insufficient data	102	58	0	754	insufficient data	cnl: P1.7-2,30-5: F1-2: ST-53 (cc53)	7-2	30-5	F1-2	28	13
72077	COL201906-43	2019	male	throat swab	Z	6502	ST-2057 complex	insufficient data	25	24	0		cross-reactive	Z: P1.19-1,15: F3-7: ST-6502 (cc2057)	19-1	15	F3-7	3	700
72078	COL201906-44	2019	female	throat swab	B	35	ST-35 complex	insufficient data	16	21	0	257	cross-reactive	B: P1.22-1,14: F4-1: ST-35 (cc35)	22-1	14	F4-1	4	29
72079	COL201906-54	2019	male	throat swab		2154		insufficient data	16	230	0		cross-reactive	ND: P1.22,9-5: F5-7: ST-2154 ()	22	9-5	F5-7	11	1600
72080	COL201906-55	2019	male	throat swab	B	10864	ST-269 complex	insufficient data	13	329	0		cross-reactive	B: P1.19,15-1: F1-5: ST-10864 (cc269)	19	15-1	F1-5	2	311
72081	COL201906-60	2019	male	throat swab	B	11393	ST-41/44 complex	insufficient data	499	21	0		insufficient data	B: P1.22,14-6: F1-5: ST-11393 (cc41/44)	22	14-6	F1-5	2	8
72082	COL201906-61	2019	male	throat swab	B	3200	ST-4821 complex	insufficient data	16		0		cross-reactive	B: P1.17-6,23-3: F3-36: ST-3200 (cc4821)	17-6	23-3	F3-36	12	275
72083	COL201906-64	2019	male	throat swab	Z	10866		insufficient data	16	53	0		cross-reactive	Z: P1.22-15,14-13: F-ND: ST-10866 ()	22-15	14-13		11	314
72084	COL201906-65	2019	male	throat swab	B	15169		exact match	19	2	3		cross-reactive	B: P1.7-2,ND: F1-18: ST-15169 ()	7-2		F1-18	12	2824
72085	COL201906-66	2019	male	throat swab	cnl	4221		insufficient data	106	1495	0		insufficient data	cnl: P1.7,ND: F5-148: ST-4221 ()	7		F5-148	12	29
72086	COL201906-67	2019	male	throat swab	Z	5953		insufficient data	22	601	0		insufficient data	Z: P1.18-1,30-4: F5-7: ST-5953 ()	18-1	30-4	F5-7	11	309
72087	COL201906-68	2019	male	throat swab	B	409	ST-41/44 complex	exact match	19	2	0	315	cross-reactive	B: P1.18-1,34: F1-5: ST-409 (cc41/44)	18-1	34	F1-5	2	156
72088	COL201906-69	2019	female	throat swab	B	409	ST-41/44 complex	insufficient data	19		0		cross-reactive	B: P1.18-1,34: F1-5: ST-409 (cc41/44)	18-1	34	F1-5	2	156
72089	COL201907-26	2019	male	throat swab	B	15170	ST-32 complex	insufficient data	510	29	1		cross-reactive	B: P1.18-4,25: F4-1: ST-15170 (cc32)	18-4	25	F4-1	2	1032
72090	COL201907-27	2019	male	throat swab	B	15166		insufficient data	106	819	0	2945	insufficient data	B: P1.19,15: F5-148: ST-15166 ()	19	15	F5-148	12	29
72091	COL201907-28	2019	male	rectal swab	Z	14816		insufficient data	1243	601	0		insufficient data	Z: P1.22-47,13-22: F5-7: ST-14816 ()	22-47	13-22	F5-7	11	1600
72092	COL201907-29	2019	male	throat swab	cnl	34	ST-32 complex	exact match	1	20	1	3177	cross-reactive	cnl: P1.19,15: F5-186: ST-34 (cc32)	19	15	F5-186	2	51
72093	COL201907-30	2019	male	throat swab	Z	11562		insufficient data	16	1296	0		cross-reactive	Z: P1.22,1: F5-7: ST-11562 ()	22	1	F5-7	11	1600
72094	COL201907-31	2019	male	throat swab	Y	1466	ST-174 complex	exact match	21	6	8	14	cross-reactive	Y: P1.21,16: F3-7: ST-1466 (cc174)	21	16	F3-7	2	69
72095	COL201907-34	2019	male	throat swab	B	3469	ST-4821 complex	insufficient data	16		0		cross-reactive	B: P1.17-6,23: F3-36: ST-3469 (cc4821)	17-6	23	F3-36	12	275
72096	COL201907-37	2019	male	throat swab	cnl	53	ST-53 complex	insufficient data	102	58	0		insufficient data	cnl: P1.7-28,30: F1-2: ST-53 (cc53)	7-28	30	F1-2	28	13
72097	COL201907-40	2019	male	throat swab	cnl	34	ST-32 complex	exact match	1	20	1	3177	cross-reactive	cnl: P1.19,15: F5-1: ST-34 (cc32)	19	15	F5-1	2	51
72098	COL201907-41	2019	male	throat swab	cnl	2578	ST-41/44 complex	exact match	100	2	0	4425	insufficient data	cnl: P1.17,9: F1-5: ST-2578 (cc41/44)	17	9	F1-5	4	1117
72099	COL201907-43	2019	female	other	cnl	34	ST-32 complex	exact match	1	20	1	3177	cross-reactive	cnl: P1.19,15: F5-12: ST-34 (cc32)	19	15	F5-12	2	51
72100	COL201907-44	2019	female	throat swab	cnl	34	ST-32 complex	exact match	1	20	1	3177	cross-reactive	cnl: P1.19,15: F5-12: ST-34 (cc32)	19	15	F5-12	2	51
72101	COL201907-45	2019	male	throat swab	cnl	15172		insufficient data	119	53	0	4944	insufficient data	cnl: P1.19-2,13-1: F1-5: ST-15172 ()	19-2	13-1	F1-5	3	2879
72102	COL201907-46	2019	male	throat swab	E	1649	ST-1157 complex	cross-reactive	13	10			cross-reactive	E: P1.22,9: F5-36: ST-1649 (cc1157)	22	9	F5-36	4	47
72103	COL201907-48	2019	male	throat swab	E	178	ST-178 complex	insufficient data	13	6	0		cross-reactive	E: P1.19-5,15-23: F1-7: ST-178 (cc178)	19-5	15-23	F1-7	3	835
72104	COL201907-49	2019	male	throat swab	Z	10866		insufficient data	16	669	0		cross-reactive	Z: P1.22,14-6: F5-7: ST-10866 ()	22	14-6	F5-7	11	314
72105	COL201907-51	2019	male	throat swab	cnl	53	ST-53 complex	insufficient data	544	58	0		insufficient data	cnl: P1.7,30-3: F1-2: ST-53 (cc53)	7	30-3	F1-2	28	2359
72106	COL201907-52	2019	male	urethral swab	cnl	34	ST-32 complex	exact match	1	20	1	3177	cross-reactive	cnl: P1.19,15: F5-12: ST-34 (cc32)	19	15	F5-12	2	51
72107	COL201907-53	2019	male	urethral swab	E	1649	ST-1157 complex	cross-reactive	13	10			cross-reactive	E: P1.22,9: F5-36: ST-1649 (cc1157)	22	9	F5-36	4	47

72108	COL201907-55	2019	male	throat swab	cnl	53	ST-53 complex	insufficient data	102	58	0	635	insufficient data	cnl: P1.7,30-3: F1-2: ST-53 (cc53)	7	30-3	F1-2	28	13
72109	COL201907-58	2019	female	throat swab	E	12912	ST-60 complex	insufficient data	13	24	0	237	cross-reactive	E: P1.5,2: F5-13: ST-12912 (cc60)	5	2	F5-13	3	2231
72110	COL201907-59	2019	male	throat swab	cnl	1136	ST-1136 complex	insufficient data	94	145	0	657	insufficient data	cnl: P1.18-4,25: F4-1: ST-1136 (cc1136)	18-4	25	F4-1	2	30
72111	COL201907-62	2019	male	throat swab	B	897		insufficient data	13	8	0		cross-reactive	B: P1.12,16-75: F1-5: ST-897 ()	12	16-75	F1-5	12	704
72112	COL201907-63	2019	male	throat swab	E	12410	ST-1157 complex	insufficient data	13	114	0	1532	cross-reactive	E: P1.17,9: F5-36: ST-12410 (cc1157)	17	9	F5-36	4	47
72113	COL201908-27	2019	male	throat swab	B	15079		insufficient data	101	237			insufficient data	B: P1.22-28,14-6: F3-15: ST-15079 ()	22-28	14-6	F3-15	2	22
72114	COL201908-28	2019	male	throat swab	Z	13017		insufficient data		1165	0			Z: P1.22-36,14-6: F5-7: ST-13017 ()	22-36	14-6	F5-7	11	1600
72115	COL201908-30	2019	male	throat swab	B	7612	ST-41/44 complex	exact match	19	2	0		cross-reactive	B: P1.18,25-32: F1-5: ST-7612 (cc41/44)	18	25-32	F1-5	4	17
72116	COL201908-31	2019	male	throat swab	B	5542		insufficient data	21	1060			cross-reactive	B: P1.22-1,1: F5-64: ST-5542 ()	22-1	1	F5-64	12	153
72117	COL201908-33	2019	male	throat swab	cnl	14928	ST-162 complex	insufficient data	1280	1388	0		insufficient data	cnl: P1.19-2,13: F1-62: ST-14928 (cc162)	19-2	13	F1-62	4	32
72118	COL201908-34	2019	male	throat swab	E	6119		insufficient data	16		0		cross-reactive	E: P1.12-8,16-179: F5-81: ST-6119 ()	12-8	16-179	F5-81	3	1525
72119	COL201908-37	2019	male	throat swab	B	13798	ST-41/44 complex	insufficient data	19		0		cross-reactive	B: P1.18-1,3: F1-5: ST-13798 (cc41/44)	18-1	3	F1-5	2	18
72120	COL201908-38	2019	male	throat swab	cnl	4221		insufficient data	106	819	0		insufficient data	cnl: P1.7-2,13-1: F5-148: ST-4221 ()	7-2	13-1	F5-148	12	29
72121	COL201908-41	2019	male	throat swab	B	11556	ST-32 complex	exact match	1	3	1	84	cross-reactive	B: P1.7-2,16: F3-3: ST-11556 (cc32)	7-2	16	F3-3	2	51
72122	COL201908-43	2019	male	throat swab	E	6119		insufficient data	16	231	0		cross-reactive	E: P1.12-8,16-119: F5-81: ST-6119 ()	12-8	16-119	F5-81	3	1525
72123	COL201908-44	2019	female	throat swab	cnl	53	ST-53 complex	insufficient data	102	58	0		insufficient data	cnl: P1.22,14-6: F1-2: ST-53 (cc53)	22	14-6	F1-2	28	13
72124	COL201908-47	2019	male	throat swab	cnl	1117	ST-1117 complex	insufficient data	21	239	0	755	cross-reactive	cnl: P1.18-1,30: F1-106: ST-1117 (cc1117)	18-1	30	F1-106	12	397
72125	COL201908-48	2019	male	throat swab	B	17449		cross-reactive	24	10	0	253	insufficient data	B: P1.17,16-3: F5-5: ST-17449 ()	17	16-3	F5-5	2	8
72126	COL201908-58	2019	male	throat swab	cnl	4221		insufficient data	106	819	0	2945	insufficient data	cnl: P1.19,15: F5-148: ST-4221 ()	19	15	F5-148	12	29
72127	COL201908-62	2019	male	throat swab	E	178	ST-178 complex	insufficient data	13	6	0		cross-reactive	E: P1.19-5,15-23: F1-7: ST-178 (cc178)	19-5	15-23	F1-7	3	835
72128	COL201908-64	2019	male	throat swab	cnl	1136	ST-1136 complex	insufficient data	94	145	0	657	insufficient data	cnl: P1.18-4,25: F4-1: ST-1136 (cc1136)	18-4	25	F4-1	2	30
72129	COL201908-72	2019	male	throat swab	B	409	ST-41/44 complex	exact match	19	2	0		cross-reactive	B: P1.18-1,ND: F1-5: ST-409 (cc41/44)	18-1		F1-5	2	156
72130	COL201908-75	2019	male	throat swab	cnl	16088		insufficient data	18	1511	0		insufficient data	cnl: P1.21-2,23-6: F3-8: ST-16088 ()	21-2	23-6	F3-8	361	243
72131	COL201908-79	2019	male	throat swab	B	16089	ST-213 complex	insufficient data	45				exact match	B: P1.22,14: F5-5: ST-16089 (cc213)	22	14	F5-5	3	49
72132	COL201908-81	2019	male	throat swab		13197	ST-22 complex	insufficient data	27	20	0		insufficient data	ND: P1.18-1,3: F-ND: ST-13197 (cc22)	18-1	3		4	19
72133	COL201908-87	2019	male	throat swab	cnl	823	ST-198 complex	cross-reactive	4	10	0	1486	cross-reactive	cnl: P1.18,25-22: F-ND: ST-823 (cc198)	18	25-22		3	30
72134	COL201908-88	2019	male	throat swab	cnl	34	ST-32 complex	exact match	1	20	1		cross-reactive	cnl: P1.19-2,15: F5-1: ST-34 (cc32)	19-2	15	F5-1	2	51
72135	COL201909-29	2019	male	throat swab	cnl	2153	ST-162 complex	insufficient data	21	20	0		cross-reactive	cnl: P1.18-1,30-3: F1-62: ST-2153 (cc162)	18-1	30-3	F1-62	4	32
72136	COL201909-31	2019	male	throat swab	cnl	15003		insufficient data	16	1506	0		cross-reactive	cnl: P1.22,14-13: F5-7: ST-15003 ()	22	14-13	F5-7	11	314
72137	COL201909-32	2019	male	throat swab	cnl			insufficient data	19	92	0		cross-reactive	cnl: P1.12-1,13-1: F5-24: ST-ND (-)	12-1	13-1	F5-24	4	17
72138	COL201909-33	2019	male	throat swab	E	60	ST-60 complex	insufficient data	13	24	0	237	cross-reactive	E: P1.5,2: F1-7: ST-60 (cc60)	5	2	F1-7	3	2496
72140	COL201808-68	2019	male	rectal swab	E	6119		insufficient data	16	231	0		cross-reactive	E: P1.12-8,16-119: F5-81: ST-6119 ()	12-8	16-119	F5-81	3	1525
72141	COL201909-34	2019	male	throat swab	cnl	34	ST-32 complex	exact match	1	20	1	3177	cross-reactive	cnl: P1.19,15: F5-186: ST-34 (cc32)	19	15	F5-186	2	51
72142	COL201909-35	2019	male	throat swab	cnl	53	ST-53 complex	insufficient data	102	58	0	1746	insufficient data	cnl: P1.7,30-4: F1-2: ST-53 (cc53)	7	30-4	F1-2	28	13
72143	COL201909-36	2019	male	throat swab	cnl	11563		insufficient data	18	1511	0	4356	insufficient data	cnl: P1.21-2,23-32: F3-8: ST-11563 ()	21-2	23-32	F3-8	361	243
72144	COL201909-37	2019	male	throat swab	Z	10866		insufficient data	16	601	0	871	cross-reactive	Z: P1.22,14-13: F5-7: ST-10866 ()	22	14-13	F5-7	11	314
72145	COL201909-38	2019	male	throat swab	B	3200	ST-4821 complex	insufficient data	16	248	0		cross-reactive	B: P1.17-6,23: F3-36: ST-3200 (cc4821)	17-6	23	F3-36	12	275
72146	COL201909-43	2019	male	throat swab	B	897		insufficient data	13	8	0		cross-reactive	B: P1.12,16-75: F1-5: ST-897 ()	12	16-75	F1-5	12	704
72147	COL201909-51	2019	male	throat swab	B	409	ST-41/44 complex	exact match	456	2	0		insufficient data	B: P1.18-1,34: F1-5: ST-409 (cc41/44)	18-1	34	F1-5	2	156
72149	COL201909-57	2019	male	throat swab	B	7460	ST-32 complex	insufficient data	510	29	187		cross-reactive	B: P1.18-1,30: F3-3: ST-7460 (cc32)	18-1	30	F3-3	2	32
72150	COL201909-58	2019	male	throat swab	cnl	1117	ST-1117 complex	insufficient data	21	239	0	755	cross-reactive	cnl: P1.18-1,30: F1-106: ST-1117 (cc1117)	18-1	30	F1-106	12	397
72151	COL201909-59	2019	male	throat swab	B	897		insufficient data	520	8	0		insufficient data	B: P1.12-1,ND: F1-5: ST-897 ()	12-1		F1-5	12	704
72152	COL201909-60	2019	male	throat swab	cnl	34	ST-32 complex	exact match	1	3	1		cross-reactive	cnl: P1.19-27,15: F5-1: ST-34 (cc32)	19-27	15	F5-1	2	51
72153	COL201909-62	2019	male	throat swab	E	6119		insufficient data	16	1784	0		cross-reactive	E: P1.12-8,16-8: F5-81: ST-6119 ()	12-8	16-8	F5-81	3	2596
72154	COL201909-71	2019	female	throat swab	B	7460	ST-32 complex	insufficient data	510	29	187		cross-reactive	B: P1.18-1,30-1: F3-3: ST-7460 (cc32)	18-1	30-1	F3-3	2	32
72155	COL201909-72	2019	male	throat swab	NG	213	ST-213 complex	none	45	18	0	224	exact match	NG: P1.22,14: F5-5: ST-213 (cc213)	22	14	F5-5	3	49
72156	COL201909-73	2019	male	throat swab	B	3200	ST-4821 complex	insufficient data	16	669	0	2963	cross-reactive	B: P1.17-6,23-6: F3-36: ST-3200 (cc4821)	17-6	23-6	F3-36	12	275
72157	COL201909-74	2019	female	throat swab	E			cross-reactive	13	10	149		cross-reactive	E: P1.22,9: F5-36: ST-ND (-)	22	9	F5-36	4	47
72158	COL201909-76	2019	male	throat swab	Z	10866		insufficient data	16	601	0	747	cross-reactive	Z: P1.22-4,14-13: F5-7: ST-10866 ()	22-4	14-13	F5-7	11	314
72159	COL201909-77	2019	male	throat swab	B	5542		insufficient data	21	1060			cross-reactive	B: P1.22-1,1: F5-64: ST-5542 ()	22-1	1	F5-64	12	153
72160	COL201910-26	2019	male	throat swab	B	409	ST-41/44 complex	exact match	19	2	0		cross-reactive	B: P1.18-1,34-6: F1-5: ST-409 (cc41/44)	18-1	34-6	F1-5	2	156
72161	COL201910-28	2019	male	throat swab	B	1947	ST-41/44 complex	insufficient data	16	161	0		cross-reactive	B: P1.18,14: F1-25: ST-1947 (cc41/44)	18	14	F1-25	4	8
72162	COL201910-29	2019	male	throat swab	B	409	ST-41/44 complex	exact match	19	2	0	315	cross-reactive	B: P1.18-1,34: F1-5: ST-409 (cc41/44)	18-1	34	F1-5	2	156
72163	COL201910-30	2019	male	throat swab	Y	17950	ST-23 complex	insufficient data	25	7	0	228	cross-reactive	Y: P1.5-2,10-1: F5-12: ST-17950 (cc23)	5-2	10-1	F5-12	5	90
72164	COL201910-32	2019	male	throat swab	Z	3882		insufficient data	22	601	0		insufficient data	Z: P1.18-1,30-51: F5-7: ST-3882 ()	18-1	30-51	F5-7	11	309
72165	COL201910-33	2019	male	throat swab	NG	35	ST-35 complex	insufficient data	16	21	0		cross-reactive	NG: P1.22-49,14: F4-1: ST-35 (cc35)	22-49	14	F4-1	4	29
72166	COL201910-34	2019	male	throat swab	cnl	1136	ST-1136 complex	insufficient data	94	145	0	657	insufficient data	cnl: P1.18-4,25: F4-1: ST-1136 (cc1136)	18-4	25	F4-1	2	30
72167	COL201910-35	2019	male	throat swab	cnl	34	ST-32 complex	insufficient data	101	550	1		insufficient data	cnl: P1.19-1,15: F5-1: ST-34 (cc32)	19-1	15	F5-1	2	51
72168	COL201910-36	2019	male	throat swab	E	1157	ST-1157 complex	insufficient data	13	114	0	271	cross-reactive	E: P1.21-7,16: F5-36: ST-1157 (cc1157)	21-7	16	F5-36	4	47
72169	COL201910-37	2019	female	throat swab	B	35	ST-35 complex	insufficient data		21	0			B: P1.22-1,14: F4-1: ST-35 (cc35)	22-1	14	F4-1	4	29
72170	COL201910-38	2019	male	throat swab	E	1649	ST-1157 complex	cross-reactive	13	10			cross-reactive	E: P1.22,9: F5-36: ST-1649 (cc1157)	22	9	F5-36	4	47
72171	COL201910-39	2019	female	throat swab	E	15149	ST-60 complex	insufficient data	31	24	0		insufficient data	E: P1.5,2: F5-13: ST-15149 (cc60)	5	2	F5-13	3	2231
72172	COL201910-43	2019	male	throat swab	cnl	53	ST-53 complex	insufficient data	102	58	0	637	insufficient data	cnl: P1.7,30: F1-2: ST-53 (cc53)	7	30	F1-2	28	13
72173	COL201910-44	2019	female	throat swab	Z	6502	ST-2057 complex	insufficient data	25	326	0		cross-reactive	Z: P1.19-1,15: F3-7: ST-6502 (cc2057)	19-1	15	F3-7	3	700
72174	COL201910-45	2019	male	throat swab	cnl	16090	ST-162 complex	insufficient data	21	20	0		cross-reactive	cnl: P1.12-1,23-3: F1-12: ST-16090 (cc162)	12-1	23-3	F1-12	4	32
72175	COL201910-47	2019	female	throat swab	cnl	823	ST-198 complex	cross-reactive	4	10	0	741	cross-reactive	cnl: P1.18,25-15: F5-5: ST-823 (cc198)	18	25-15	F5-5	3	30
72176	COL201910-49	2019	male	throat swab	B	7612	ST-41/44 complex	exact match	19	2	0		cross-reactive	B: P1.18,1: F1-34: ST-7612 (cc41/44)	18	1	F1-34	4	17
72177	COL201910-50	2019	male	throat swab	E	5768		insufficient data	436	336	0		insufficient data	E: P1.22,14-6: F5-7: ST-5768 ()	22	14-6	F5-7		2900
72178	COL201910-58	2019	male	throat swab	Z	5953		insufficient data	22	470	0		insufficient data	Z: P1.18-1,30-11: F5-7: ST-5953 ()	18-1	30-11	F5-7	11	309

72179	COL201910-60	2019	male	throat swab	E	16091	ST-1157 complex	cross-reactive	13	10	149		cross-reactive	E: P1.22-25,ND: F5-36: ST-16091 (cc1157)	22-25		F5-36	4	47
72180	COL201910-64	2019	male	throat swab	B	897		insufficient data	13	8	0		cross-reactive	B: P1.12,16-75: F1-5: ST-897 ()	12	16-75	F1-5	12	704
72181	COL201910-73	2019	male	throat swab	Z	3882		insufficient data	22	601	0		insufficient data	Z: P1.ND,30-8: F5-7: ST-3882 ()		30-8	F5-7	11	309
72182	COL201910-74	2019	male	throat swab	cnl	2578	ST-41/44 complex	exact match	100	2	0	4425	insufficient data	cnl: P1.17,9: F1-5: ST-2578 (cc41/44)	17	9	F1-5	4	1117
72183	COL201910-76	2019	male	throat swab	cnl	4221		insufficient data	106	819	0		insufficient data	cnl: P1.7-2,13-2: F5-148: ST-4221 ()	7-2	13-2	F5-148	12	29
72184	COL201910-81	2019	male	throat swab	E	1157	ST-1157 complex	insufficient data	13	114	0	271	cross-reactive	E: P1.21-7,16: F5-36: ST-1157 (cc1157)	21-7	16	F5-36	4	47
72185	COL201910-82	2019	male	rectal swab	B	13427	ST-4821 complex	insufficient data	16	669	0	811	cross-reactive	B: P1.17-6,23: F3-36: ST-13427 (cc4821)	17-6	23	F3-36	12	2901
72186	COL201910-83	2019	male	throat swab	B	13427	ST-4821 complex	insufficient data	16	669	0	811	cross-reactive	B: P1.17-6,23: F3-36: ST-13427 (cc4821)	17-6	23	F3-36	12	2901
72187	COL201910-84	2019	male	throat swab	B	11527	ST-32 complex	insufficient data	101	237			insufficient data	B: P1.22,14-6: F3-15: ST-11527 (cc32)	22	14-6	F3-15	2	22
72188	COL201911-28	2019	female	throat swab	Z	10866		insufficient data	16	601	0		cross-reactive	Z: P1.22,ND: F5-7: ST-10866 ()	22		F5-7	11	314
72189	COL201911-29	2019	male	throat swab	B	32	ST-32 complex	insufficient data		3	1			B: P1.7,16: F3-3: ST-32 (cc32)	7	16	F3-3	2	51
72190	COL201911-31	2019	male	throat swab	Z	10866		insufficient data	456	6	0		insufficient data	Z: P1.22,14-25: F5-7: ST-10866 ()	22	14-25	F5-7	11	314
72191	COL201911-32	2019	male	throat swab	cnl	4221		insufficient data	106	819	0		insufficient data	cnl: P1.7-2,13-2: F5-148: ST-4221 ()	7-2	13-2	F5-148	12	29
72193	COL201911-35	2019	male	throat swab	cnl	2578	ST-41/44 complex	exact match	100	2	0	4425	insufficient data	cnl: P1.17,9: F1-5: ST-2578 (cc41/44)	17	9	F1-5	4	1117
72194	COL201911-36	2019	male	throat swab	cnl	53	ST-53 complex	insufficient data	102	58	0	635	insufficient data	cnl: P1.7,30-3: F1-2: ST-53 (cc53)	7	30-3	F1-2	28	13
72195	COL201911-38	2019	male	throat swab	Z	3882		insufficient data	22	601	0		insufficient data	Z: P1.18-1,ND: F5-7: ST-3882 ()	18-1		F5-7	11	309
72196	COL201911-39	2019	male	throat swab	cnl	14928	ST-162 complex	insufficient data	1280	1388	0		insufficient data	cnl: P1.19-2,13: F1-62: ST-14928 (cc162)	19-2	13	F1-62	4	32
72197	COL201911-40	2019	male	throat swab	B	2160	ST-750 complex	insufficient data	16	1022	0		cross-reactive	B: P1.22,30-8: F5-7: ST-2160 (cc750)	22	30-8	F5-7	28	2837
72198	COL201911-42	2019	male	throat swab	B	6595	ST-4821 complex	insufficient data	16	234	0	3728	cross-reactive	B: P1.20,23: F3-36: ST-6595 (cc4821)	20	23	F3-36	12	275
72199	COL201911-44	2019	male	throat swab	cnl	16088		insufficient data	18	1511	0		insufficient data	cnl: P1.21-2,23-6: F3-8: ST-16088 ()	21-2	23-6	F3-8	361	243
72200	COL201911-46	2019	male	throat swab	cnl	16093	ST-1136 complex	insufficient data		145	0			cnl: P1.18-4,25: F4-1: ST-16093 (cc1136)	18-4	25	F4-1	2	30
72201	COL201911-48	2019	male	throat swab	B	1572	ST-1572 complex	insufficient data	260	20	0	3179	insufficient data	B: P1.7-12,14: F1-7: ST-1572 (cc1572)	7-12	14	F1-7	11	2902
72202	COL201911-51	2019	male	throat swab	cnl	14928	ST-162 complex	insufficient data	1280	1388	0		insufficient data	cnl: P1.19-2,13: F1-62: ST-14928 (cc162)	19-2	13	F1-62	4	32
72203	COL201911-54	2019	male	throat swab	cnl			insufficient data	544	749			insufficient data	cnl: P1.ND,ND: F5-5: ST-ND (-)			F5-5		
72204	COL201911-57	2019	male	throat swab	cnl	16094	ST-213 complex	insufficient data	102	1286	0		insufficient data	cnl: P1.19,15: F5-5: ST-16094 (cc213)	19	15	F5-5	3	49
72205	COL201911-64	2019	male	throat swab	B	897		insufficient data	13	8	0		cross-reactive	B: P1.12,16-75: F1-5: ST-897 ()	12	16-75	F1-5	12	704
72206	COL201911-65	2019	female	throat swab	B	565	ST-269 complex	insufficient data	19		0		cross-reactive	B: P1.19,13-1: F3-9: ST-565 (cc269)	19	13-1	F3-9	12	29
72207	COL201911-66	2019	male	throat swab	B	35	ST-35 complex	insufficient data	16	21	0	257	cross-reactive	B: P1.22-1,14: F4-1: ST-35 (cc35)	22-1	14	F4-1	4	29
72208	COL201912-26	2019	female	throat swab	B	409	ST-41/44 complex	exact match	19	2	0	315	cross-reactive	B: P1.18-1,34: F1-5: ST-409 (cc41/44)	18-1	34	F1-5	2	156
72209	COL201912-27	2019	male	throat swab	Z	3882		insufficient data	22	601	0	2512	insufficient data	Z: P1.18-1,30: F5-7: ST-3882 ()	18-1	30	F5-7	11	309
72210	COL201912-28	2019	male	throat swab	B	575	ST-213 complex	insufficient data	45	117	0	4346	exact match	B: P1.22,14: F1-7: ST-575 (cc213)	22	14	F1-7	3	72
72211	COL201912-29	2019	male	throat swab	Z	10866		insufficient data	16		0		cross-reactive	Z: P1.22,14-65: F5-7: ST-10866 ()	22	14-65	F5-7	11	314
72212	COL201912-30	2019	male	throat swab	X	15164	ST-1572 complex	insufficient data	260	20	0	4041	insufficient data	X: P1.7-13,14: F1-34: ST-15164 (cc1572)	7-13	14	F1-34	11	31
72213	COL201912-34	2019	male	throat swab	E	1157	ST-1157 complex	insufficient data	13	114	0	271	cross-reactive	E: P1.21-7,16: F5-36: ST-1157 (cc1157)	21-7	16	F5-36	4	47
72214	COL201912-35	2019	female	throat swab	cnl	278	ST-35 complex	insufficient data	24	21	0	750	insufficient data	cnl: P1.7-2,13-1: F1-7: ST-278 (cc35)	7-2	13-1	F1-7	4	2903
72215	COL201912-37	2019	female	throat swab	cnl	823	ST-198 complex	cross-reactive	4	10	0	1740	cross-reactive	cnl: P1.19-1,15-11: F5-5: ST-823 (cc198)	19-1	15-11	F5-5	3	30
72216	COL201912-40	2019	male	throat swab	C	11	ST-11 complex	insufficient data	896	20	2		insufficient data	C: P1.5-1,10-8: F3-6: ST-11 (cc11)	5-1	10-8	F3-6	4	1632
72217	COL201912-41	2019	male	throat swab	E	6119		insufficient data	16	1172	0		cross-reactive	E: P1.18-1,3: F2-1: ST-6119 ()	18-1	3	F2-1	3	1525
72218	COL201903-73	2019	male	throat swab	cnl	53	ST-53 complex	insufficient data	102	291	0		insufficient data	cnl: P1.7-2,30: F1-2: ST-53 (cc53)	7-2	30	F1-2	28	13
72219	COL201903-42	2019	female	throat swab	NG	7245	ST-35 complex	insufficient data	13	21	0	3929	cross-reactive	NG: P1.22-1,14: F-ND: ST-7245 (cc35)	22-1	14		4	29
72220	COL201812-51	2018	male	throat swab	NG	15160	ST-35 complex	insufficient data	16	21	0	257	cross-reactive	NG: P1.22-1,14: F-ND: ST-15160 (cc35)	22-1	14		374	29
72221	COL201812-33	2018	female	throat swab		865	ST-865 complex	insufficient data	19		0		cross-reactive	ND: P1.7-1,1: F1-6: ST-865 (cc865)	7-1	1	F1-6	4	84
72222	COL201811-33	2018	male	throat swab	NG	457	ST-35 complex	insufficient data	16	21			cross-reactive	NG: P1.22-1,14-15: F4-1: ST-457 (cc35)	22-1	14-15	F4-1	4	29
72223	COL201810-72	2018	female	throat swab	cnl	53	ST-53 complex	insufficient data	102	291	0		insufficient data	cnl: P1.7-2,30: F1-2: ST-53 (cc53)	7-2	30	F1-2	28	13
72224	COL201810-48	2018	female	throat swab	NG	11736	ST-41/44 complex	exact match	252	2	0		cross-reactive	NG: P1.18-1,34: F3-13: ST-11736 (cc41/44)	18-1	34	F3-13	2	8
72225	COL201810-37	2018	male	throat swab	cnl	2578	ST-41/44 complex	exact match	100	2	0	3598	insufficient data	cnl: P1.12-1,13-46: F1-5: ST-2578 (cc41/44)	12-1	13-46	F1-5	4	17
72226	COL201809-71	2018	male	throat swab	NG	35	ST-35 complex	insufficient data	16	21	0		cross-reactive	NG: P1.22-49,14: F4-1: ST-35 (cc35)	22-49	14	F4-1	4	29
72227	COL201809-37	2018	male	throat swab	NG	414	ST-41/44 complex	exact match	19	2	0		cross-reactive	NG: P1.18-47,25-15: F1-5: ST-414 (cc41/44)	18-47	25-15	F1-5	4	17
72228	COL201809-28	2018	male	throat swab	NG	6119		insufficient data	16	816	0		cross-reactive	NG: P1.12-8,23-6: F5-81: ST-6119 ()	12-8	23-6	F5-81	4	1525
72229	COL201808-28	2018	female	throat swab	NG	6119		insufficient data	16	816	0		cross-reactive	NG: P1.12-8,23-6: F5-81: ST-6119 ()	12-8	23-6	F5-81	4	1525
72230	COL201807-50	2018	male	throat swab		1466	ST-174 complex	exact match	21	6	8	14	cross-reactive	ND: P1.21,16: F3-7: ST-1466 (cc174)	21	16	F3-7	2	69
72231	COL201805-59	2018	male	throat swab	NG	15152	ST-35 complex	insufficient data	27	21	0		insufficient data	NG: P1.22-1,14: F-ND: ST-15152 (cc35)	22-1	14		4	29
72232	COL201804-44	2018	male	throat swab	NG	4924	ST-103 complex	insufficient data	25	24	0	1208	cross-reactive	NG: P1.5-1,10-7: F3-9: ST-4924 (cc103)	5-1	10-7	F3-9	3	37
72697	COL201803-39	2018	male	throat swab	NG	11397	ST-32 complex	insufficient data	510	29	1	1338	cross-reactive	NG: P1.18-1,30-2: F3-3: ST-11397 (cc32)	18-1	30-2	F3-3	2	51
72698	COL201806-50	2018	male	urethral swab	B	7612	ST-41/44 complex	exact match	19	2	0	1986	cross-reactive	B: P1.18,25: F1-5: ST-7612 (cc41/44)	18	25	F1-5	146	17
72699	COL201904-27	2019	male	throat swab	cnl	53	ST-53 complex	insufficient data	102	58	0	637	insufficient data	cnl: P1.7,30: F1-2: ST-53 (cc53)	7	30	F1-2	28	13
72700	COL201904-42	2019	male	throat swab	NG	414	ST-41/44 complex	exact match	19	2	0		cross-reactive	NG: P1.18-47,25: F1-5: ST-414 (cc41/44)	18-47	25	F1-5	4	17
72702	COL201912-39	2019	male	rectal swab	C	11	ST-11 complex	insufficient data	896	20	2		insufficient data	C: P1.5-1,10-8: F3-6: ST-11 (cc11)	5-1	10-8	F3-6	4	1632
72703	COL201912-42	2019	male	throat swab	B	3200	ST-4821 complex	insufficient data	16	669	0		cross-reactive	B: P1.17-6,23-35: F3-36: ST-3200 (cc4821)	17-6	23-35	F3-36	12	275
72704	COL201912-45	2019	male	throat swab	B	13011	ST-4821 complex	insufficient data	16	669	0	811	cross-reactive	B: P1.17-6,23: F3-36: ST-13011 (cc4821)	17-6	23	F3-36	12	275
72705	COL201912-47	2019	male	throat swab	B	897		insufficient data	13	8	0		cross-reactive	B: P1.12,16-75: F1-5: ST-897 ()	12	16-75	F1-5	12	704
72706	COL201912-48	2019	male	throat swab	E	1649	ST-1157 complex	cross-reactive	13	10	165		cross-reactive	E: P1.22,9: F5-36: ST-1649 (cc1157)	22	9	F5-36	4	47
72707	COL201912-52	2019	male	throat swab	cnl	14928	ST-162 complex	insufficient data	1280	1388	0		insufficient data	cnl: P1.19-2,13: F1-62: ST-14928 (cc162)	19-2	13	F1-62	4	32
72708	COL201912-53	2019	male	throat swab		1466	ST-174 complex	exact match	21	6	8	14	cross-reactive	ND: P1.21,16: F3-7: ST-1466 (cc174)	21	16	F3-7	2	69
72709	COL201912-55	2019	male	throat swab	E	6119		insufficient data	16	816	0		cross-reactive	E: P1.12-8,23-6: F5-81: ST-6119 ()	12-8	23-6	F5-81	4	1525
72710	COL201912-57	2019	male	throat swab	cnl	15024		insufficient data	1282	819	0		insufficient data	cnl: P1.18-1,3: F5-148: ST-15024 ()	18-1	3	F5-148	12	2841
72711	COL201912-59	2019	male	throat swab	B	13427	ST-4821 complex	insufficient data	16	669	0	811	cross-reactive	B: P1.17-6,23: F3-36: ST-13427 (cc4821)	17-6	23	F3-36	12	2901
72712	COL201912-66	2019	female	throat swab	E	1157	ST-1157 complex	insufficient data	13	114	0	271	cross-reactive	E: P1.21-7,16: F5-36: ST-1157 (cc1157)	21-7	16	F5-36	4	47
72713	COL201912-71	2019	male	throat swab	cnl	1136	ST-1136 complex	insufficient data	257	145	0		insufficient data	cnl: P1.18-4,25: F1-19: ST-1136 (cc1136)	18-4	25	F1-19	2	2993

72714	COL201912-72	2019	male	throat swab	X	8930		insufficient data	19	1084	179		cross-reactive	X: P1.22,9: F1-5: ST-8930 ()	22	9	F1-5	12	1001
72715	COL201912-75	2019	male	throat swab	cnl	53	ST-53 complex	insufficient data		58	0			cnl: P1.7,30: F1-2: ST-53 (cc53)	7	30	F1-2	28	13
72716	COL201912-81	2019	male	throat swab	Y	1466	ST-174 complex	exact match	21	6	8	14	cross-reactive	Y: P1.21,16: F3-7: ST-1466 (cc174)	21	16	F3-7	2	69
72717	COL201912-82	2019	male	throat swab	B	3200	ST-4821 complex	insufficient data	16	669	0	811	cross-reactive	B: P1.17-6,23: F1-80: ST-3200 (cc4821)	17-6	23	F1-80	12	275
72718	COL201912-83	2019	male	throat swab	C	11	ST-11 complex	insufficient data	896	20	2		insufficient data	C: P1.5-1,10-8: F3-6: ST-11 (cc11)	5-1	10-8	F3-6	4	1
72719	COL201912-85	2019	male	throat swab	cnl	11563		insufficient data	18	1511	0		insufficient data	cnl: P1.21-2,ND: F3-8: ST-11563 ()	21-2		F3-8	361	243
92904	COL201801-37	2018	male	throat swab	cnl			insufficient data		58	0			cnl: P1.ND,30-3: F3-7,F5-148,F5-8: ST-ND (-)		30-3	-7;F5-148;F	28	
92905	COL201801-43	2018	male	throat swab	NG	13798	ST-41/44 complex	insufficient data	19		0		cross-reactive	NG: P1.18-1,3: F1-5: ST-13798 (cc41/44)	18-1	3	F1-5	2	18
92906	COL201801-62	2018	male	throat swab	Z	5953		insufficient data	22	601	0	4888	insufficient data	Z: P1.18-1,30-8: F5-7: ST-5953 ()	18-1	30-8	F5-7	11	309
92908	COL201803-26	2018	female	throat swab	cnl	34	ST-32 complex	exact match	1	20	1	3177	cross-reactive	cnl: P1.19,15: F5-12: ST-34 (cc32)	19	15	F5-12	2	2931
92909	COL201803-27	2018	male	throat swab	B	16524		insufficient data	21	1615	0		cross-reactive	B: P1.12-1,16-80: F5-2: ST-16524 ()	12-1	16-80	F5-2		32
92910	COL201803-29	2018	male	throat swab	NG	17395	ST-53 complex	insufficient data	102	58	0	651	insufficient data	NG: P1.7,30-5: F1-2: ST-17395 (cc53)	7	30-5	F1-2	28	13
92911	COL201803-30	2018	male	throat swab	NG	823	ST-198 complex	cross-reactive	4	10	0	741	cross-reactive	NG: P1.18,25-15: F5-33: ST-823 (cc198)	18	25-15	F5-33	3	30
92912	COL201803-31	2018	male	throat swab	cnl	11563		insufficient data	18	1511	0	4356	insufficient data	cnl: P1.21-2,23-32: F3-8: ST-11563 ()	21-2	23-32	F3-8	361	243
92913	COL201803-35	2018	male	throat swab	cnl	4221		insufficient data	106	819	0		insufficient data	cnl: P1.7-2,13-43: F5-148: ST-4221 ()	7-2	13-43	F5-148	12	29
92914	COL201803-38	2018	male	throat swab	cnl	34	ST-32 complex	exact match	1	20	1	3177	cross-reactive	cnl: P1.19,15: F5-1: ST-34 (cc32)	19	15	F5-1	2	51
92916	COL201803-40	2018	male	throat swab	B			exact match	19	2	0	315	cross-reactive	B: P1.18-1,34: F1-5: ST-ND (-)	18-1	34	F1-5	2	156
92917	COL201803-41	2018	female	throat swab	B	16825		insufficient data	25	24	0		cross-reactive	B: P1.7-2,ND: F1-20: ST-16825 ()	7-2		F1-20	3	1580
92918	COL201803-42	2018	female	throat swab	NG	11397	ST-32 complex	insufficient data	510	29	1	1338	cross-reactive	NG: P1.18-1,30-2: F3-3: ST-11397 (cc32)	18-1	30-2	F3-3	2	51
92919	COL201803-45	2018	male	throat swab	Z	10866		insufficient data	16	601	0	871	cross-reactive	Z: P1.22,14-13: F1-68: ST-10866 ()	22	14-13	F1-68	11	314
92920	COL201803-46	2018	male	throat swab	NG	53	ST-53 complex	insufficient data	102	58	0	637	insufficient data	NG: P1.7,30: F1-2: ST-53 (cc53)	7	30	F1-2	28	13
92921	COL201803-47	2018	female	throat swab	Z	10866		insufficient data	16	601	0	871	cross-reactive	Z: P1.22,14-13: F6-2: ST-10866 ()	22	14-13	F6-2	11	314
92922	COL201803-48	2018	male	throat swab	cnl	53	ST-53 complex	insufficient data	102	58	0	1266	insufficient data	cnl: P1.7-2,30-2: F1-2: ST-53 (cc53)	7-2	30-2	F1-2	28	13
92923	COL201803-57	2018	male	throat swab	NG	13033	ST-461 complex	insufficient data	592	1515	0		insufficient data	NG: P1.19-2,13-1: F3-9: ST-13033 (cc461)	19-2	13-1	F3-9	2	60
92924	COL201803-59	2018	male	throat swab	NG	1117	ST-1117 complex	insufficient data	21	239	0	2051	cross-reactive	NG: P1.18-1,30-1: F3-7: ST-1117 (cc1117)	18-1	30-1	F3-7	12	397
92925	COL201803-60	2018	male	throat swab	NG	1136	ST-1136 complex	insufficient data	94	145	0	657	insufficient data	NG: P1.18-4,25: F-ND: ST-1136 (cc1136)	18-4	25		2	30
92926	COL201803-61	2018	male	throat swab	cnl	1136	ST-1136 complex	insufficient data	94	145	0	657	insufficient data	cnl: P1.18-4,25: F1-68: ST-1136 (cc1136)	18-4	25	F1-68	2	30
92927	COL201803-62	2018	male	throat swab	cnl	823	ST-198 complex	cross-reactive	4	31	0		cross-reactive	cnl: P1.18,25-15: F5-5: ST-823 (cc198)	18	25-15	F5-5	3	2932
92928	COL201803-63	2018	female	throat swab	cnl	34	ST-32 complex	exact match	1	20	1		cross-reactive	cnl: P1.19,15-40: F5-1: ST-34 (cc32)	19	15-40	F5-1	2	51
92929	COL201803-66	2018	male	throat swab	cnl	53	ST-53 complex	insufficient data		58	0			cnl: P1.7-2,30: F1-7: ST-53 (cc53)	7-2	30	F1-7	28	13
92930	COL201803-67	2018	male	urethral swab	cnl	823	ST-198 complex	cross-reactive	4	10	0		cross-reactive	cnl: P1.22-1,14: F5-5: ST-823 (cc198)	22-1	14	F5-5	3	30
92931	COL201803-68	2018	male	throat swab	B	409	ST-41/44 complex	exact match	19	2	0	315	cross-reactive	B: P1.18-1,34: F1-5: ST-409 (cc41/44)	18-1	34	F1-5	2	156
92933	COL201803-75	2018	male	throat swab	Z	6502	ST-2057 complex	insufficient data	25	24	0		cross-reactive	Z: P1.19-1,15: F3-7: ST-6502 (cc2057)	19-1	15	F3-7	3	700
92934	COL201803-82	2018	male	throat swab	NG	11397	ST-32 complex	insufficient data	510	29	1	1338	cross-reactive	NG: P1.18-1,30-2: F3-3: ST-11397 (cc32)	18-1	30-2	F3-3	2	51
92935	COL201803-83	2018	male	throat swab	B			insufficient data	101		0		insufficient data	B: P1.12-1,23: F3-21: ST-ND (-)	12-1	23	F3-21	2	
92936	COL201804-26	2018	male	throat swab	Z	3882		insufficient data	22	291	0		insufficient data	Z: P1.18-1,30-11: F5-7: ST-3882 ()	18-1	30-11	F5-7	11	309
92937	COL201804-28	2018	female	throat swab	B			insufficient data	13	114	0	373	cross-reactive	B: P1.22,14-6: F5-36: ST-ND (-)	22	14-6	F5-36	4	2933
92938	COL201804-29	2018	male	throat swab	NG	16525	ST-1136 complex	insufficient data	94	145	0		insufficient data	NG: P1.18-4,13-2: F4-1: ST-16525 (cc1136)	18-4	13-2	F4-1	2	30
92940	COL201804-32	2018	male	throat swab	cnl	4221		insufficient data	106	819	0		insufficient data	cnl: P1.7-2,13: F5-148: ST-4221 ()	7-2	13	F5-148	12	29
92941	COL201804-34	2018	female	throat swab	B	15148		insufficient data	257		0		insufficient data	B: P1.18-1,34-4: F1-19: ST-15148 ()	18-1	34-4	F1-19	2	156
92942	COL201804-35	2018	male	throat swab	E			insufficient data	13	114	0	271	cross-reactive	E: P1.21-7,16: F5-36: ST-ND (-)	21-7	16	F5-36	4	47
92943	COL201804-36	2018	male	throat swab	E	1157	ST-1157 complex	insufficient data	13	114	0	271	cross-reactive	E: P1.21-7,16: F5-36: ST-1157 (cc1157)	21-7	16	F5-36	4	47
92944	COL201804-37	2018	male	throat swab	cnl	53	ST-53 complex	insufficient data	102	58	0	637	insufficient data	cnl: P1.7,30: F1-2: ST-53 (cc53)	7	30	F1-2	28	13
92945	COL201804-38	2018	male	throat swab	cnl	823	ST-198 complex	cross-reactive	4	10	0		cross-reactive	cnl: P1.18-1,3-4: F-ND: ST-823 (cc198)	18-1	3-4		3	30
92947	COL201804-46	2018	female	throat swab	cnl	53	ST-53 complex	insufficient data	102	58	0	637	insufficient data	cnl: P1.7,30: F1-2: ST-53 (cc53)	7	30	F1-2	28	13
92948	COL201804-47	2018	male	throat swab	B	3469	ST-4821 complex	insufficient data	16	1436	0		cross-reactive	B: P1.17-6,23-6: F3-36: ST-3469 (cc4821)	17-6	23-6	F3-36	12	275
92949	COL201804-49	2018	male	throat swab	E	1157	ST-1157 complex	insufficient data	13	114	0	2028	cross-reactive	E: P1.21-7,16-83: F5-36: ST-1157 (cc1157)	21-7	16-83	F5-36	4	47
92950	COL201804-51	2018	male	throat swab	cnl	53	ST-53 complex	insufficient data	102	58	0	1744	insufficient data	cnl: P1.7-2,30: F1-2: ST-53 (cc53)	7-2	30	F1-2	28	13
92951	COL201804-58	2018	male	throat swab	NG	14931	ST-41/44 complex	exact match	19	2	0	315	cross-reactive	NG: P1.18-1,34: F1-5: ST-14931 (cc41/44)	18-1	34	F1-5	2	156
92952	COL201804-66	2018	female	throat swab	E	11739	ST-60 complex	insufficient data	13	24	0	237	cross-reactive	E: P1.5,2: F5-13: ST-11739 (cc60)	5	2	F5-13	3	2231
92954	COL201804-68	2018	female	throat swab	NG	409	ST-41/44 complex	exact match	19	2	0	315	cross-reactive	NG: P1.18-1,34: F1-5: ST-409 (cc41/44)	18-1	34	F1-5	2	156
92955	COL201804-69	2018	male	throat swab	E	178	ST-178 complex	cross-reactive	12	6	0	4351	insufficient data	E: P1.19,15: F1-18: ST-178 (cc178)	19	15	F1-18	2	144
92956	COL201804-73	2018	male	throat swab	cnl	53	ST-53 complex	insufficient data	102	58	0	640	insufficient data	cnl: P1.7,30-2: F1-2: ST-53 (cc53)	7	30-2	F1-2	28	13
92957	COL201804-74	2018	male	throat swab	cnl	823	ST-198 complex	cross-reactive	4	10	0	3213	cross-reactive	cnl: P1.17-4,9: F5-5: ST-823 (cc198)	17-4	9	F5-5	3	30
92958	COL201804-75	2018	male	throat swab	NG	13798	ST-41/44 complex	insufficient data	19		0		cross-reactive	NG: P1.18-1,3: F1-5: ST-13798 (cc41/44)	18-1	3	F1-5	2	18
92959	COL201804-81	2018	male	throat swab	NG	897		insufficient data	13	8	0		cross-reactive	NG: P1.12,16-75: F1-5: ST-897 ()	12	16-75	F1-5	12	704
92960	COL201805-05	2018	male	urethral swab	Z	2123		insufficient data	16	101	0		cross-reactive	Z: P1.18,25-15: F5-7: ST-2123 ()	18	25-15	F5-7	11	172
92961	COL201805-29	2018	male	throat swab	cnl	34	ST-32 complex	exact match	1	20	1	3177	cross-reactive	cnl: P1.19,15: F5-1: ST-34 (cc32)	19	15	F5-1	2	51
92962	COL201805-31	2018	male	throat swab	E	1157	ST-1157 complex	insufficient data	476	114	0		insufficient data	E: P1.21-7,16: F5-36: ST-1157 (cc1157)	21-7	16	F5-36	4	47
92963	COL201805-32	2018	male	throat swab	cnl	823	ST-198 complex	cross-reactive	4	10	0		cross-reactive	cnl: P1.18-1,3-4: F-ND: ST-823 (cc198)	18-1	3-4		3	30
92964	COL201805-33	2018	female	throat swab	NG	15049	ST-198 complex	cross-reactive	4	10	0		cross-reactive	NG: P1.12-1,13: F-ND: ST-15049 (cc198)	12-1	13		3	30
92965	COL201805-34	2018	female	throat swab	cnl	1117	ST-1117 complex	insufficient data	21		0		cross-reactive	cnl: P1.18-1,30: F3-7: ST-1117 (cc1117)	18-1	30	F3-7	12	397
92966	COL201805-36	2018	male	throat swab	B	136	ST-41/44 complex	cross-reactive	24	10	0	253	insufficient data	B: P1.17,16-3: F5-5: ST-136 (cc41/44)	17	16-3	F5-5	2	8
92967	COL201805-37	2018	male	throat swab	cnl	4221		insufficient data	106	1495	0		insufficient data	cnl: P1.7,ND: F5-148: ST-4221 ()	7		F5-148	12	29
92968	COL201805-38	2018	male	throat swab	Z	12926		insufficient data	16	601	0		cross-reactive	Z: P1.17-6,23-7: F5-7: ST-12926 ()	17-6	23-7	F5-7	4	1600
92969	COL201805-39	2018	male	throat swab	E	15150		insufficient data	16	1172	0		cross-reactive	E: P1.12-8,16-8: F5-81: ST-15150 ()	12-8	16-8	F5-81	3	2596
92970	COL201805-40	2018	male	throat swab	E	1157	ST-1157 complex	insufficient data	24	114	0	2541	insufficient data	E: P1.21-7,16: F5-36: ST-1157 (cc1157)	21-7	16	F5-36	4	
92971	COL201805-42	2018	male	throat swab	NG	53	ST-53 complex	insufficient data	102	58	0	635	insufficient data	NG: P1.7,30-3: F1-2: ST-53 (cc53)	7	30-3	F1-2	28	13
92972	COL201805-43	2018	male	throat swab	cnl	53	ST-53 complex	insufficient data	102	58	0	1745	insufficient data	cnl: P1.7-2,30-3: F1-2: ST-53 (cc53)	7-2	30-3	F1-2	28	13

92974	COl201805-45	2018	female	throat swab	E	1649	ST-1157 complex	cross-reactive	13	10			cross-reactive	E: P1.22,9: F5-36: ST-1649 (cc1157)	22	9	F5-36	4	47
92975	COL201805-46	2018	male	throat swab	Z	10866		insufficient data	16		0		cross-reactive	Z: P1.22-4,14-13: F5-7: ST-10866 ()	22-4	14-13	F5-7	11	1581
92977	COL201805-49	2018	male	throat swab	cnl	823	ST-198 complex	cross-reactive	4	10	0	741	cross-reactive	cnl: P1.18,25-15: F5-5: ST-823 (cc198)	18	25-15	F5-5	3	30
92978	COL201805-50	2018	male	throat swab	cnl	2578	ST-41/44 complex	exact match	100	2	0	4425	insufficient data	cnl: P1.17,9: F1-5: ST-2578 (cc41/44)	17	9	F1-5	4	1117
92979	COL201805-54	2018	male	throat swab	C	32	ST-32 complex	exact match	1	3	1	144	cross-reactive	C: P1.7,16-29: F3-3: ST-32 (cc32)	7	16-29	F3-3	2	51
92980	COL201805-55	2018	female	throat swab	cnl	823	ST-198 complex	cross-reactive	4	10	0		cross-reactive	cnl: P1.18-1,3-4: F-ND: ST-823 (cc198)	18-1	3-4		3	30
92981	COL201805-56	2018	male	throat swab	Z	10866		insufficient data	16	601	0	747	cross-reactive	Z: P1.22-4,14-13: F5-7: ST-10866 ()	22-4	14-13	F5-7	11	314
92983	COL201805-64	2018	male	throat swab	E	1649	ST-1157 complex	cross-reactive	13	10			cross-reactive	E: P1.22,9: F5-36: ST-1649 (cc1157)	22	9	F5-36	4	47
92984	COL201805-68	2018	male	throat swab	cnl	15024		insufficient data	1282	819	0		insufficient data	cnl: P1.18-44,3: F5-148: ST-15024 ()	18-44	3	F5-148	12	2841
92985	COL201805-69	2018	female	throat swab	cnl	53	ST-53 complex	insufficient data	102	58	0	1745	insufficient data	cnl: P1.7-2,30-3: F1-2: ST-53 (cc53)	7-2	30-3	F1-2	28	13
92986	COL201806-27	2018	male	throat swab	Z	5953		insufficient data	22		0		insufficient data	Z: P1.18-1,30-11: F5-7: ST-5953 ()	18-1	30-11	F5-7	11	309
92988	COL201806-30	2018	male	throat swab	cnl	34	ST-32 complex	exact match	1	20	1	3177	cross-reactive	cnl: P1.19,15: F5-1: ST-34 (cc32)	19	15	F5-1	2	51
92989	COL201806-32	2018	male	throat swab	B	7460	ST-32 complex	insufficient data		29	1			B: P1.22,14: F3-3: ST-7460 (cc32)	22	14	F3-3	2	1032
92990	COL201806-35	2018	male	throat swab	B			insufficient data	16	161	0		cross-reactive	B: P1.18,14: F1-25: ST-ND (-)	18	14	F1-25	4	1849
92991	COL201806-36	2018	male	throat swab	NG	44	ST-41/44 complex	exact match	19	2	0		cross-reactive	NG: P1.20,23-28: F1-18: ST-44 (cc41/44)	20	23-28	F1-18	2	8
92993	COL201806-38	2018	male	throat swab	cnl	34	ST-32 complex	exact match	1	20	1	3177	cross-reactive	cnl: P1.19,15: F5-1: ST-34 (cc32)	19	15	F5-1	2	51
92994	COL201806-39	2018	male	throat swab	NG	13019	ST-4821 complex	insufficient data	16	669	0	811	cross-reactive	NG: P1.17-6,23: F3-36: ST-13019 (cc4821)	17-6	23	F3-36	12	275
92996	COL201806-45	2018	male	throat swab	B	3200	ST-4821 complex	insufficient data	16	669	0		cross-reactive	B: P1.17-6,23-7: F3-36: ST-3200 (cc4821)	17-6	23-7	F3-36	12	275
92997	COL201806-48	2018	male	throat swab	E	1649	ST-1157 complex	cross-reactive	13	10			cross-reactive	E: P1.22,9: F5-36: ST-1649 (cc1157)	22	9	F5-36	4	47
92998	COL201806-66	2018	male	throat swab	NG	2160	ST-750 complex	insufficient data	16	239	0		cross-reactive	NG: P1.7-2,13-9: F5-7: ST-2160 (cc750)	7-2	13-9	F5-7	28	1600
92999	COL201806-67	2018	male	throat swab	cnl	14928	ST-162 complex	insufficient data	1280	1388	0		insufficient data	cnl: P1.19-2,13: F1-62: ST-14928 (cc162)	19-2	13	F1-62	4	32
93000	COL201806-68	2018	male	throat swab	NG	1947	ST-41/44 complex	insufficient data	16	161	0		cross-reactive	NG: P1.18,14: F1-25: ST-1947 (cc41/44)	18	14	F1-25	4	8
93001	COL201806-69	2018	male	throat swab	NG	41	ST-41/44 complex	exact match	24	26	0		insufficient data	NG: P1.7-2,4: F1-5: ST-41 (cc41/44)	7-2	4	F1-5	4	17
93002	COL201806-70	2018	male	throat swab	cnl	1117	ST-1117 complex	insufficient data	21		0		cross-reactive	cnl: P1.18-1,30: F3-7: ST-1117 (cc1117)	18-1	30	F3-7	12	1251
93003	COL201806-71	2018	female	throat swab	NG	409	ST-41/44 complex	insufficient data	19		0		cross-reactive	NG: P1.18-1,34: F1-5: ST-409 (cc41/44)	18-1	34	F1-5	2	156
93004	COL201806-72	2018	male	throat swab	Z	15143		insufficient data	22	329	0		insufficient data	Z: P1.5-1,10-85: F5-7: ST-15143 ()	5-1	10-85	F5-7	11	309
93005	COL201806-77	2018	male	throat swab	cnl	823	ST-198 complex	cross-reactive	4		0		cross-reactive	cnl: P1.7-2,16-82: F5-5: ST-823 (cc198)	7-2	16-82	F5-5	3	30
93006	COL201806-80	2018	male	throat swab	Z	5953		insufficient data	22		0		insufficient data	Z: P1.18-1,30-5: F5-7: ST-5953 ()	18-1	30-5	F5-7	11	309
93007	COL201806-81	2018	male	throat swab	cnl	4221		insufficient data	106	819	0		insufficient data	cnl: P1.7-2,13: F5-148: ST-4221 ()	7-2	13	F5-148	12	2927
93008	COL201806-86	2018	female	throat swab	cnl	53	ST-53 complex	insufficient data	102		0		insufficient data	cnl: P1.7,30: F1-2: ST-53 (cc53)	7	30	F1-2	28	13
93009	COL201806-87	2018	female	throat swab	cnl	2384	ST-198 complex	cross-reactive	4	10	0		cross-reactive	cnl: P1.17,9-5: F5-5: ST-2384 (cc198)	17	9-5	F5-5	3	30
93010	COL201807-30	2018	male	throat swab	Z	3882		insufficient data	22	601	0		insufficient data	Z: P1.18-1,30-11: F5-7: ST-3882 ()	18-1	30-11	F5-7	11	309
93011	COL201807-31	2018	female	throat swab	B	11397	ST-32 complex	insufficient data	510	29	1	1806	cross-reactive	B: P1.18-1,30-11: F3-3: ST-11397 (cc32)	18-1	30-11	F3-3	2	51
93012	COL201807-32	2018	male	throat swab	cnl	7129		insufficient data	627	912			insufficient data	cnl: P1.12-6,13-13: F5-5: ST-7129 ()	12-6	13-13	F5-5	105	36
93013	COL201807-34	2018	female	throat swab	cnl	278	ST-35 complex	insufficient data	24	21	0	4884	insufficient data	cnl: P1.7-2,13-66: F1-7: ST-278 (cc35)	7-2	13-66	F1-7	4	2928
93014	COL201807-36	2018	female	throat swab	cnl	34	ST-32 complex	exact match	1	20	1	3177	cross-reactive	cnl: P1.19,15: F5-1: ST-34 (cc32)	19	15	F5-1	2	51
93015	COL201807-38	2018	male	throat swab		1466	ST-174 complex	exact match	21	6	8	14	cross-reactive	ND: P1.21,16: F1-2,F3-7: ST-1466 (cc174)	21	16	F1-2;F3-7	2	69
93016	COL201807-39	2018	male	throat swab	B			insufficient data	520	8	0	4413	insufficient data	B: P1.12,16-75: F1-5: ST-ND (-)	12	16-75	F1-5	12	704
93017	COL201807-40	2018	female	throat swab	cnl	34	ST-32 complex	exact match	1	20	0		cross-reactive	cnl: P1.18,25-1: F5-1: ST-34 (cc32)	18	25-1	F5-1	2	51
93018	COL201807-42	2018	female	throat swab	E	15047	ST-1157 complex	cross-reactive	13	10	165		cross-reactive	E: P1.22,9: F5-36: ST-15047 (cc1157)	22	9	F5-36	4	47
93020	COL201807-44	2018	male	throat swab	E	15048	ST-269 complex	cross-reactive	15	21	0	405	cross-reactive	E: P1.18-1,3: F1-7: ST-15048 (cc269)	18-1	3	F1-7	12	19
93021	COL201807-47	2018	male	urethral swab	NG	11	ST-11 complex	insufficient data	896	20	2		insufficient data	NG: P1.5-1,10-8: F3-6: ST-11 (cc11)	5-1	10-8	F3-6	4	1
93023	COL201807-51	2018	male	throat swab				exact match	21		8		cross-reactive	ND: P1.ND,13: F3-7,F5-148: ST-ND (-)		13	F3-7;F5-148	12	1582
93026	COL201807-64	2018	male	throat swab	B	11515	ST-1572 complex	insufficient data	260	20	0	2940	insufficient data	B: P1.7-36,14: F1-7: ST-11515 (cc1572)	7-36	14	F1-7	11	31
93027	COL201807-65	2018	female	throat swab	cnl	53	ST-53 complex	insufficient data	102	58	0	651	insufficient data	cnl: P1.7,30-5: F1-2: ST-53 (cc53)	7	30-5	F1-2	28	13
93028	COL201807-66	2018	male	throat swab	cnl	53	ST-53 complex	insufficient data	102	58	0	651	insufficient data	cnl: P1.7,30-5: F1-2: ST-53 (cc53)	7	30-5	F1-2	28	13
93029	COL201807-70	2018	male	throat swab	cnl	53	ST-53 complex	insufficient data	102	58	0	637	insufficient data	cnl: P1.7,30: F1-2: ST-53 (cc53)	7	30	F1-2	28	13
93031	COL201808-27	2018	male	throat swab	E	178	ST-178 complex	cross-reactive	12	6	0		insufficient data	E: P1.19-1,15: F5-28: ST-178 (cc178)	19-1	15	F5-28	3	144
93033	COL201808-29	2018	male	throat swab	cnl	15386	ST-32 complex	exact match	1	3	1	5	cross-reactive	cnl: P1.19,15: F5-1: ST-15386 (cc32)	19	15	F5-1	2	51
93034	COL201808-33	2018	male	throat swab	cnl	10866		insufficient data	16	601	0	3718	cross-reactive	cnl: P1.22,14-56: F5-7: ST-10866 ()	22	14-56	F5-7	11	314
93036	COL201808-37	2018	male	throat swab	NG	278	ST-35 complex	insufficient data	24	21	0	750	insufficient data	NG: P1.7-2,13-1: F1-7: ST-278 (cc35)	7-2	13-1	F1-7	4	29
93037	COL201808-38	2018	male	throat swab	cnl	11312		insufficient data	544				insufficient data	cnl: P1.22-1,1: F5-5: ST-11312 ()	22-1	1	F5-5	105	36
93038	COL201808-40	2018	female	throat swab	cnl			insufficient data	94	145	0	657	insufficient data	cnl: P1.18-4,25: F4-1: ST-ND (-)	18-4	25	F4-1	2	30
93039	COl201808-41	2018	male	throat swab	cnl	53	ST-53 complex	insufficient data	544	670	0		insufficient data	cnl: P1.7,30: F1-2: ST-53 (cc53)	7	30	F1-2	28	2359
93040	COL201808-42	2018	male	rectal swab	cnl	2578	ST-41/44 complex	exact match	100	2	0	3211	insufficient data	cnl: P1.18,25-1: F1-5: ST-2578 (cc41/44)	18	25-1	F1-5	4	1117
93041	COL201808-46	2018	male	throat swab	NG	414	ST-41/44 complex	exact match	19	2	0		cross-reactive	NG: P1.18-47,25: F1-5: ST-414 (cc41/44)	18-47	25	F1-5	4	17
93042	COL201808-47	2018	male	throat swab	cnl	53	ST-53 complex	insufficient data	102	58	0	637	insufficient data	cnl: P1.7,30: F1-2: ST-53 (cc53)	7	30	F1-2	28	13
93043	COL201808-48	2018	male	throat swab	E	6119		insufficient data	16		0		cross-reactive	E: P1.12-8,16-179: F5-81: ST-6119 ()	12-8	16-179	F5-81	3	1525
93044	COL201808-49	2018	male	throat swab	E	1157	ST-1157 complex	insufficient data	13	114	0	271	cross-reactive	E: P1.21-7,16: F5-36: ST-1157 (cc1157)	21-7	16	F5-36	4	47
93045	COL201808-50	2018	male	throat swab		23	ST-23 complex	insufficient data	25	7	0	228	cross-reactive	ND: P1.5-2,10-1: F4-1: ST-23 (cc23)	5-2	10-1	F4-1	1	70
93046	COL201808-51	2018	male	throat swab	cnl	53	ST-53 complex	insufficient data	102	58	0	635	insufficient data	cnl: P1.7,30-3: F1-2: ST-53 (cc53)	7	30-3	F1-2	28	13
93047	COL201808-54	2018	male	throat swab	B	11395	ST-32 complex	exact match	1	3	1	4	cross-reactive	B: P1.7,16: F3-3: ST-11395 (cc32)	7	16	F3-3	2	51
93048	COL201808-62	2018	male	throat swab	NG	11395	ST-32 complex	exact match	1	3	1	4	cross-reactive	NG: P1.7,16: F3-3: ST-11395 (cc32)	7	16	F3-3	2	51
93050	COL201808-72	2018	male	throat swab	NG	6119		insufficient data	16	231	0		cross-reactive	NG: P1.12-8,16-119: F5-81: ST-6119 ()	12-8	16-119	F5-81	3	1525
93051	COL201809-27	2018	male	throat swab	Z	3882		insufficient data	22	601	0	1896	insufficient data	Z: P1.18-1,30-1: F5-7: ST-3882 ()	18-1	30-1	F5-7	11	309
93053	COL201809-33	2018	male	throat swab	B	12480	ST-4821 complex	insufficient data	16	669	0	2396	cross-reactive	B: P1.20,23: F1-5: ST-12480 (cc4821)	20	23	F1-5	12	275
93055	COL201809-38	2018	male	throat swab	Z	15064		insufficient data	22	912	0		insufficient data	Z: P1.12-6,13-39: F5-5: ST-15064 ()	12-6	13-39	F5-5	11	309
93056	COL201809-40	2018	male	throat swab	B			insufficient data	19	29	0		cross-reactive	B: P1.12-8,13-39: F1-18,F5-5: ST-ND (-)	12-8	13-39	F1-18;F5-5	2	8
93057	COL201809-43	2018	male	throat swab	NG	2160	ST-750 complex	insufficient data	16	1526	0		cross-reactive	NG: P1.22,13: F5-7: ST-2160 (cc750)	22	13	F5-7	28	1600

93058	COL201809-45	2018	female	throat swab	cnl	34	ST-32 complex	exact match	1	248	1		cross-reactive	cnl: P1.19,15: F5-1: ST-34 (cc32)	19	15	F5-1	2	51
93059	COL201809-46	2018	male	throat swab	NG	4221		insufficient data	106	819	0		insufficient data	NG: P1.18,25: F1-62: ST-4221 ()	18	25	F1-62	12	29
93060	COL201809-53	2018	male	throat swab	NG	32	ST-32 complex	exact match	1	92	1		cross-reactive	NG: P1.7,16-66: F3-3: ST-32 (cc32)	7	16-66	F3-3	2	51
93061	COL201809-56	2018	female	throat swab	E	1649	ST-1157 complex	insufficient data	1265	291	165		insufficient data	E: P1.22,9-31: F1-68: ST-1649 (cc1157)	22	9-31	F1-68	4	47
93062	COL201809-57	2018	male	throat swab	E	1649	ST-1157 complex	insufficient data	1265	291	165		insufficient data	E: P1.22,9-31: F1-68: ST-1649 (cc1157)	22	9-31	F1-68	4	47
93063	COL201809-58	2018	male	throat swab	cnl	53	ST-53 complex	insufficient data	102	58	0	1744	insufficient data	cnl: P1.7-2,30: F1-2: ST-53 (cc53)	7-2	30	F1-2	28	
93065	COL201809-63	2018	male	throat swab	C	2731	ST-41/44 complex	cross-reactive	19	1	0		cross-reactive	C: P1.5-1,2-99: F5-2: ST-2731 (cc41/44)	5-1	2-99	F5-2	2	8
93067	COL201809-72	2018	male	throat swab	NG	3200	ST-4821 complex	insufficient data	16	669	0	811	cross-reactive	NG: P1.17-6,23: F3-36: ST-3200 (cc4821)	17-6	23	F3-36	12	275
93068	COL201810-26	2018	male	throat swab	Z	10866		insufficient data	16	897	0		cross-reactive	Z: P1.22,14-13: F5-7: ST-10866 ()	22	14-13	F5-7	11	314
93069	COL201810-28	2018	male	throat swab	NG	467	ST-269 complex	cross-reactive	15	21	0	405	cross-reactive	NG: P1.18-1,3: F1-7: ST-467 (cc269)	18-1	3	F1-7	12	19
93070	COL201810-30	2018	male	throat swab	B	409	ST-41/44 complex	insufficient data	19		0		cross-reactive	B: P1.18-1,34: F1-5: ST-409 (cc41/44)	18-1	34	F1-5	2	156
93072	COL201810-33	2018	female	throat swab	cnl	1136	ST-1136 complex	insufficient data	94	145	0	657	insufficient data	cnl: P1.18-4,25: F-ND: ST-1136 (cc1136)	18-4	25		2	30
93073	COL201810-34	2018	female	throat swab	NG	34	ST-32 complex	exact match	1	20	1	3177	cross-reactive	NG: P1.19,15: F5-1: ST-34 (cc32)	19	15	F5-1	2	51
93074	COL201810-35	2018	female	throat swab	cnl	823	ST-198 complex	cross-reactive	4	10	0	597	cross-reactive	cnl: P1.18,25-14: F5-1: ST-823 (cc198)	18	25-14	F5-1	3	30
93075	COL201810-36	2018	male	throat swab	NG	11527	ST-32 complex	insufficient data	101	237	0	1921	insufficient data	NG: P1.22,14-6: F3-15: ST-11527 (cc32)	22	14-6	F3-15	2	22
93079	COL201810-45	2018	male	throat swab	E	15154	ST-60 complex	insufficient data	13	24	0	237	cross-reactive	E: P1.5,2: F5-13: ST-15154 (cc60)	5	2	F5-13	3	2231
93081	COL201810-51	2018	female	throat swab	E	15073	ST-1157 complex	insufficient data	13	114	0	271	cross-reactive	E: P1.21-7,16: F5-36: ST-15073 (cc1157)	21-7	16	F5-36	4	47
93082	COL201810-53	2018	female	throat swab	cnl	53	ST-53 complex	insufficient data	102	58	0	635	insufficient data	cnl: P1.7,30-3: F1-2: ST-53 (cc53)	7	30-3	F1-2	28	13
93083	COL201810-55	2018	male	throat swab	B	11397	ST-32 complex	insufficient data	510	29	1		cross-reactive	B: P1.17,9: F3-3: ST-11397 (cc32)	17	9	F3-3	2	51
93084	COL201810-66	2018	female	throat swab	C	11	ST-11 complex	insufficient data	1036	20	0		insufficient data	C: P1.5-1,10-8: F3-6: ST-11 (cc11)	5-1	10-8	F3-6	4	1
93086	COL201810-74	2018	female	throat swab	NG	15155	ST-1157 complex	cross-reactive	13	10			cross-reactive	NG: P1.22,2-59: F5-36: ST-15155 (cc1157)	22	2-59	F5-36	4	47
93087	COL201810-79	2018	male	throat swab	B	1417	ST-35 complex	insufficient data	16	921	0	2149	cross-reactive	B: P1.22,9: F1-22: ST-1417 (cc35)	22	9	F1-22	4	910
93088	COL201810-80	2018	male	throat swab	C	1679	ST-35 complex	insufficient data	16	21	0	257	cross-reactive	C: P1.22-1,14: F4-1: ST-1679 (cc35)	22-1	14	F4-1	4	231
93089	COL201810-81	2018	male	throat swab	NG	15156	ST-198 complex	cross-reactive	4		0		cross-reactive	NG: P1.18,25-15: F5-5: ST-15156 (cc198)	18	25-15	F5-5	3	30
93090	COL201811-12	2018	male	urethral swab	C	11	ST-11 complex	insufficient data	896	20	2		insufficient data	C: P1.5-1,10-8: F3-6: ST-11 (cc11)	5-1	10-8	F3-6	4	1
93091	COL201811-26	2018	female	throat swab	cnl	1136	ST-1136 complex	insufficient data	94	145	0		insufficient data	cnl: P1.18-19,25-26: F4-1: ST-1136 (cc1136)	18-19	25-26	F4-1	2	30
93092	COL201811-27	2018	female	throat swab	E	1157	ST-1157 complex	insufficient data	13	114			cross-reactive	E: P1.21-7,16: F5-36: ST-1157 (cc1157)	21-7	16	F5-36	4	47
93093	COL201811-28	2018	male	throat swab		23	ST-23 complex	insufficient data	25		0		cross-reactive	ND: P1.5-2,10-1: F5-12: ST-23 (cc23)	5-2	10-1	F5-12	5	90
93094	COL201811-29	2018	male	throat swab	B	3469	ST-4821 complex	insufficient data	16	1436	0		cross-reactive	B: P1.17-6,23-6: F3-36: ST-3469 (cc4821)	17-6	23-6	F3-36	12	275
93095	COL201811-30	2018	male	throat swab	E	1649	ST-1157 complex	cross-reactive	13	10			cross-reactive	E: P1.22,ND: F5-36: ST-1649 (cc1157)	22		F5-36	4	47
93096	COL201811-32	2018	female	throat swab	cnl	823	ST-198 complex	cross-reactive	4	10	0	1485	cross-reactive	cnl: P1.17,9: F5-5: ST-823 (cc198)	17	9	F5-5	3	30
93098	COL201811-35	2018	male	throat swab	cnl	53	ST-53 complex	insufficient data	102		0		insufficient data	cnl: P1.7,30: F1-2: ST-53 (cc53)	7	30	F1-2	28	13
93099	COL201811-37	2018	male	throat swab	B	1157	ST-1157 complex	insufficient data	13	114	0	373	cross-reactive	B: P1.22,14-6: F5-36: ST-1157 (cc1157)	22	14-6	F5-36	4	221
93100	COL201811-38	2018	male	throat swab	NG	35	ST-35 complex	insufficient data	16	21	0	257	cross-reactive	NG: P1.22-1,14: F-ND: ST-35 (cc35)	22-1	14		4	29
93101	COL201811-39	2018	male	throat swab	B	15157		insufficient data	520	8	0	4720	insufficient data	B: P1.12-1,16-75: F1-5: ST-15157 ()	12-1	16-75	F1-5	12	704
93102	COL201811-40	2018	female	throat swab	cnl	53	ST-53 complex	insufficient data	102	58	0	635	insufficient data	cnl: P1.7,30-3: F1-2: ST-53 (cc53)	7	30-3	F1-2	28	13
93103	COL201811-41	2018	male	throat swab	Z	3882		insufficient data	22		0		insufficient data	Z: P1.18-1,30-8: F5-7: ST-3882 ()	18-1	30-8	F5-7	11	309
93104	COL201811-43	2018	male	throat swab	B	3200	ST-4821 complex	insufficient data	16	669	0	811	cross-reactive	B: P1.17-6,23: F3-36: ST-3200 (cc4821)	17-6	23	F3-36	12	275
93105	COL201811-44	2018	male	throat swab	Z	5953		insufficient data	22	601	0		insufficient data	Z: P1.18-1,30-11: F5-7: ST-5953 ()	18-1	30-11	F5-7	11	309
93106	COL201811-46	2018	male	throat swab	B	461	ST-461 complex	insufficient data	47	118	0	230	cross-reactive	B: P1.19-2,13-1: F3-9: ST-461 (cc461)	19-2	13-1	F3-9	2	60
93108	COL201811-49	2018	male	throat swab	Z	3882		insufficient data	22	601	0		insufficient data	Z: P1.18-42,30-8: F5-7: ST-3882 ()	18-42	30-8	F5-7	11	309
93109	COL201811-50	2018	male	throat swab	NG	6119		insufficient data	16	1172	0		cross-reactive	NG: P1.12-8,16-8: F2-1: ST-6119 ()	12-8	16-8	F2-1	3	2596
93111	COL201811-52	2018	male	throat swab	Z	3882		insufficient data	22		0		insufficient data	Z: P1.18-1,30-1: F5-7: ST-3882 ()	18-1	30-1	F5-7	11	309
93112	COL201811-56	2018	female	throat swab	cnl	823	ST-198 complex	cross-reactive	4	149	0		cross-reactive	cnl: P1.ND,25-37: F5-5: ST-823 (cc198)		25-37	F5-5	3	30
93116	COL201811-62	2018	male	throat swab	NG	1784	ST-32 complex	insufficient data	101	1372	0		insufficient data	NG: P1.7-2,16-151: F1-95: ST-1784 (cc32)	7-2	16-151	F1-95	2	22
93117	COL201812-28	2018	female	throat swab	E	12475	ST-1157 complex	cross-reactive	13	10			cross-reactive	E: P1.22,9: F5-36: ST-12475 (cc1157)	22	9	F5-36	4	47
93118	COL201812-29	2018	male	throat swab	Z	15158		insufficient data	22	601	0	1896	insufficient data	Z: P1.18-1,30-1: F5-7: ST-15158 ()	18-1	30-1	F5-7	11	309
93121	COL201812-34	2018	male	throat swab		865	ST-865 complex	insufficient data	19		0		cross-reactive	ND: P1.7-1,1: F1-6: ST-865 (cc865)	7-1	1	F1-6	4	84
93122	COL201812-35	2018	male	throat swab	cnl	11564		insufficient data	119		0		insufficient data	cnl: P1.19-5,ND: F1-5: ST-11564 ()	19-5		F1-5	3	2879
93123	COL201812-36	2018	male	throat swab	NG	6595	ST-4821 complex	insufficient data	16	234	0	3728	cross-reactive	NG: P1.20,23: F3-36: ST-6595 (cc4821)	20	23	F3-36	12	3301
93124	COL201812-39	2018	male	throat swab	cnl	4221		insufficient data	106	1495	0		insufficient data	cnl: P1.7,ND: F5-148: ST-4221 ()	7		F5-148	12	29
93125	COL201812-40	2018	male	throat swab	B	6988		insufficient data	19	34	0		cross-reactive	B: P1.17-1,23: F1-5: ST-6988 ()	17-1	23	F1-5	12	1001
93126	COL201812-41	2018	male	throat swab	Z	3882		insufficient data	22	601	0	2512	insufficient data	Z: P1.18-1,30: F5-7: ST-3882 ()	18-1	30	F5-7	11	309
93127	COL201812-42	2018	female	throat swab	cnl	53	ST-53 complex	insufficient data	102	58	0	635	insufficient data	cnl: P1.7,30-3: F1-2: ST-53 (cc53)	7	30-3	F1-2	28	13
93128	COL201812-43	2018	male	throat swab	cnl	53	ST-53 complex	insufficient data	102	58	0	637	insufficient data	cnl: P1.7,30: F1-2: ST-53 (cc53)	7	30	F1-2	28	13
93129	COL201812-45	2018	male	throat swab	E	15149	ST-60 complex	insufficient data	13	24	0	237	cross-reactive	E: P1.5,2: F5-13: ST-15149 (cc60)	5	2	F5-13	3	2231
93130	COL201812-46	2018	male	throat swab	cnl	53	ST-53 complex	insufficient data	102	58	0	1746	insufficient data	cnl: P1.7,30-4: F1-2: ST-53 (cc53)	7	30-4	F1-2	28	13
93131	COL201812-47	2018	male	throat swab	NG	13019	ST-4821 complex	insufficient data	16	669	0		cross-reactive	NG: P1.17-6,23-7: F3-36: ST-13019 (cc4821)	17-6	23-7	F3-36	12	275
93132	COL201812-50	2018	male	throat swab	NG	6321		cross-reactive	4	10	0		cross-reactive	NG: P1.18,25-29: F5-5: ST-6321 ()	18	25-29	F5-5	3	30
93134	COL201812-57	2018	female	throat swab	Z	6502	ST-2057 complex	insufficient data	25	24	0		cross-reactive	Z: P1.19-1,15: F3-7: ST-6502 (cc2057)	19-1	15	F3-7		700
93135	COL201812-59	2018	female	throat swab	cnl	53	ST-53 complex	insufficient data	102	58	0	640	insufficient data	cnl: P1.7,30-2: F1-2: ST-53 (cc53)	7	30-2	F1-2	28	13
93136	COL201812-61	2019	male	throat swab	B	3200	ST-4821 complex	insufficient data	16	669	0		cross-reactive	B: P1.17-6,23-35: F3-36: ST-3200 (cc4821)	17-6	23-35	F3-36	12	275
93137	COL201901-26	2019	female	throat swab	B	32	ST-32 complex	insufficient data	21		100		cross-reactive	B: P1.7,16: F3-3: ST-32 (cc32)	7	16	F3-3	2	51
93138	COL201901-27	2019	male	throat swab	B	15161		insufficient data	1284		0		insufficient data	B: P1.17,9-4: F1-7: ST-15161 ()	17	9-4	F1-7	2	8
93139	COL201901-28	2019	male	throat swab	NG	409	ST-41/44 complex	exact match	19	2	0	315	cross-reactive	NG: P1.18-1,34: F1-5: ST-409 (cc41/44)	18-1	34	F1-5	2	156
93140	COL201901-30	2019	male	throat swab	NG	3200	ST-4821 complex	insufficient data	16	669	0	811	cross-reactive	NG: P1.17-6,23: F3-36: ST-3200 (cc4821)	17-6	23	F3-36	12	2934
93141	COL201901-31	2019	male	throat swab		1466	ST-174 complex	exact match	21	6	8	14	cross-reactive	ND: P1.21,16: F3-7: ST-1466 (cc174)	21	16	F3-7	2	69
93142	COL201901-32	2019	male	throat swab	cnl	278	ST-35 complex	insufficient data	24	21	0	750	insufficient data	cnl: P1.7-2,13-1: F1-7: ST-278 (cc35)	7-2	13-1	F1-7	4	29
93143	COL201901-33	2019	female	throat swab	E	12912	ST-60 complex	insufficient data	13	24	0	237	cross-reactive	E: P1.5,2: F5-13: ST-12912 (cc60)	5	2	F5-13	3	2231

93144	COL201901-34	2019	male	throat swab	NG	53	ST-53 complex	insufficient data	102	58	0	635	insufficient data	NG: P1.7,30-3: F1-2: ST-53 (cc53)	7	30-3	F1-2	28	13
93145	COL201901-35	2019	male	throat swab	Z	6502	ST-2057 complex	insufficient data	967	24	0		insufficient data	Z: P1.19,15: F3-7: ST-6502 (cc2057)	19	15	F3-7	3	700
93146	COL201901-36	2019	male	throat swab	cnl	53	ST-53 complex	insufficient data	102	58	0	637	insufficient data	cnl: P1.7,30: F1-2: ST-53 (cc53)	7	30	F1-2	28	2947
93147	COL201901-38	2019	male	throat swab	cnl	823	ST-198 complex	cross-reactive	4	10	0		cross-reactive	cnl: P1.18,25-78: F5-5: ST-823 (cc198)	18	25-78	F5-5	3	30
93148	COL201901-39	2019	female	throat swab	NG	1136	ST-1136 complex	insufficient data		145	0			NG: P1.18-19,25-26: F4-1: ST-1136 (cc1136)	18-19	25-26	F4-1	2	30
93149	COL201901-40	2019	male	throat swab	NG	467	ST-269 complex	insufficient data	210	21	0		insufficient data	NG: P1.17,9: F1-7: ST-467 (cc269)	17	9	F1-7	12	19
93150	COL201901-45	2019	female	throat swab	Z	6502	ST-2057 complex	insufficient data	25	24	0		cross-reactive	Z: P1.19-1,15: F3-7: ST-6502 (cc2057)	19-1	15	F3-7	3	700
93152	COL201901-50	2019	female	throat swab	B	14377	ST-41/44 complex	exact match	19	2	0		cross-reactive	B: P1.19-5,13: F1-5: ST-14377 (cc41/44)	19-5	13	F1-5	2	23
93153	COL201901-52	2019	female	throat swab	cnl	823	ST-198 complex	cross-reactive	1019	10	0		insufficient data	cnl: P1.18-1,3-4: F-ND: ST-823 (cc198)	18-1	3-4		3	30
93154	COL201901-53	2019	male	throat swab	cnl	15072		insufficient data	106	291	0		insufficient data	cnl: P1.7-2,13: F5-148: ST-15072 ()	7-2	13	F5-148	12	29
93155	COL201901-57	2019	male	throat swab	cnl	4221		insufficient data	106	819	0		insufficient data	cnl: P1.22,14-6: F3-7: ST-4221 ()	22	14-6	F3-7	12	29
93156	COL201901-59	2019	female	throat swab	E	15073	ST-1157 complex	insufficient data	13	114	0	271	cross-reactive	E: P1.21-7,16: F5-36: ST-15073 (cc1157)	21-7	16	F5-36	4	47
93157	COL201901-61	2019	female	throat swab	NG	409	ST-41/44 complex	exact match	19	2	0	315	cross-reactive	NG: P1.18-1,34: F1-5: ST-409 (cc41/44)	18-1	34	F1-5	2	156
93158	COL201901-63	2019	female	throat swab	E	1157	ST-1157 complex	insufficient data	13	114			cross-reactive	E: P1.21-7,16: F5-36: ST-1157 (cc1157)	21-7	16	F5-36	4	47
93159	COL201902-30	2019	male	throat swab	NG	15081	ST-213 complex	none	45	18	0		exact match	NG: P1.18-7,9: F5-5: ST-15081 (cc213)	18-7	9	F5-5	3	49
93160	COL201902-33	2019	male	throat swab	X	8930		insufficient data	19	1084	0		cross-reactive	X: P1.22,9: F1-5: ST-8930 ()	22	9	F1-5	12	1001
93161	COL201902-37	2019	male	throat swab	B	1572	ST-1572 complex	insufficient data	260	20	0	2940	insufficient data	B: P1.7-36,14: F1-7: ST-1572 (cc1572)	7-36	14	F1-7	3	31
93162	COL201902-42	2019	male	throat swab	C	15082		cross-reactive	24	10	0		insufficient data	C: P1.5,2-7: F5-5: ST-15082 ()	5	2-7	F5-5	2	8
93163	COL201902-46	2019	female	throat swab	cnl	2578	ST-41/44 complex	exact match	100	2	0	4425	insufficient data	cnl: P1.17,9: F1-5: ST-2578 (cc41/44)	17	9	F1-5	4	1117
93167	COL201902-52	2019	male	throat swab	NG	32	ST-32 complex	exact match	1	3	1	4	cross-reactive	NG: P1.7,16: F3-3: ST-32 (cc32)	7	16	F3-3	2	51
93168	COL201902-54	2019	male	throat swab	B	3200	ST-4821 complex	insufficient data	16	669	0	2396	cross-reactive	B: P1.20,23: F1-5: ST-3200 (cc4821)	20	23	F1-5	12	275
93169	COL201902-56	2019	male	throat swab	cnl	53	ST-53 complex	insufficient data	102	58	0	640	insufficient data	cnl: P1.7,30-2: F1-84: ST-53 (cc53)	7	30-2	F1-84	28	13
93170	COL201903-26	2019	male	throat swab	B	409	ST-41/44 complex	exact match	19	2	0	315	cross-reactive	B: P1.18-1,34: F1-5: ST-409 (cc41/44)	18-1	34	F1-5	2	156
93171	COL201903-27	2019	male	throat swab	cnl	53	ST-53 complex	insufficient data	102	58	0	1746	insufficient data	cnl: P1.7,30-4: F1-2: ST-53 (cc53)	7	30-4	F1-2	28	13
93172	COL201903-28	2019	male	throat swab	E	6119		insufficient data	27		0		insufficient data	E: P1.12-8,16-119: F2-9: ST-6119 ()	12-8	16-119	F2-9	3	1525
93173	COL201903-30	2019	female	throat swab	B	1572	ST-1572 complex	insufficient data	260		0		insufficient data	B: P1.5,2: F5-12: ST-1572 (cc1572)	5	2	F5-12	11	31
93174	COL201903-31	2019	female	throat swab	NG	823	ST-198 complex	cross-reactive	4	10	0	741	cross-reactive	NG: P1.18,25-15: F5-33: ST-823 (cc198)	18	25-15	F5-33	3	30
93175	COL201903-32	2019	male	throat swab	E	12912	ST-60 complex	insufficient data		24	0			E: P1.5,2: F5-13: ST-12912 (cc60)	5	2	F5-13	3	2231
93177	COL201903-34	2019	male	urethral swab	NG	414	ST-41/44 complex	exact match	19	2	0		cross-reactive	NG: P1.18-47,25-65: F1-5: ST-414 (cc41/44)	18-47	25-65	F1-5	4	17
93178	COL201903-35	2019	female	throat swab	NG	15035		insufficient data	16	19	0		cross-reactive	NG: P1.12-1,16-8: F3-6: ST-15035 ()	12-1	16-8	F3-6	12	2491
93179	COL201903-37	2019	female	throat swab	E	9852	ST-60 complex	insufficient data	13	24	0	237	cross-reactive	E: P1.5,2: F5-8: ST-9852 (cc60)	5	2	F5-8	3	79
93180	COL201903-38	2019	male	throat swab	E	12475	ST-1157 complex	cross-reactive	13	10			cross-reactive	E: P1.22,9: F5-36: ST-12475 (cc1157)	22	9	F5-36	4	47
93181	COL201903-41	2019	male	throat swab	cnl	823	ST-198 complex	cross-reactive	4	10	0		cross-reactive	cnl: P1.18,25-101: F5-5: ST-823 (cc198)	18	25-101	F5-5	3	30
93183	COL201903-43	2019	male	throat swab	C	11	ST-11 complex	insufficient data		20	0			C: P1.5-1,10-8: F3-6: ST-11 (cc11)	5-1	10-8	F3-6	4	1
93184	COL201903-44	2019	male	throat swab	cnl	53	ST-53 complex	insufficient data	102	58	0	635	insufficient data	cnl: P1.7,30-3: F1-2: ST-53 (cc53)	7	30-3	F1-2	28	13
93185	COL201903-47	2019	male	throat swab	B	3200	ST-4821 complex	insufficient data	16	248	0		cross-reactive	B: P1.17-6,23: F3-36: ST-3200 (cc4821)	17-6	23	F3-36	12	275
93186	COL201903-48	2019	male	throat swab	E	6119		insufficient data	16	1172	0		cross-reactive	E: P1.12-8,ND: F2-2: ST-6119 ()	12-8		F2-2	3	2596
93187	COL201903-52	2019	male	throat swab	Z	3882		insufficient data	22	601	0		insufficient data	Z: P1.22,14-6: F5-7: ST-3882 ()	22	14-6	F5-7	105	2936
93188	COL201903-54	2019	male	throat swab	Z	12943		exact match	1278	101	8		insufficient data	Z: P1.7-2,13-1: F5-7: ST-12943 ()	7-2	13-1	F5-7	4	29
93189	COL201903-55	2019	male	throat swab	B	4221		insufficient data	106	248	0		insufficient data	B: P1.19-1,15: F5-148: ST-4221 ()	19-1	15	F5-148	12	29
93190	COL201903-59	2019	male	throat swab	cnl	53	ST-53 complex	insufficient data	102	58	0	651	insufficient data	cnl: P1.7,30-5: F1-2: ST-53 (cc53)	7	30-5	F1-2	28	13
93191	COL201903-64	2019	female	throat swab	cnl	4221		insufficient data	106	819	0		insufficient data	cnl: P1.5,2: F5-148: ST-4221 ()	5	2	F5-148	12	29
93192	COL201903-66	2019	male	throat swab		1466	ST-174 complex	exact match	21	6	8	14	cross-reactive	ND: P1.21,16: F3-7: ST-1466 (cc174)	21	16	F3-7	2	69
93193	COL201903-71	2019	male	throat swab	cnl	34	ST-32 complex	exact match	1	20	1	3177	cross-reactive	cnl: P1.19,15: F5-1: ST-34 (cc32)	19	15	F5-1	2	51
93195	COL201904-32	2019	male	throat swab	NG			cross-reactive	15	21	0	405	cross-reactive	NG: P1.18-1,3: F1-7: ST-ND (-)	18-1	3	F1-7	12	19
93196	COL201904-37	2019	male	throat swab	Z	5953		insufficient data	22	601	0	4693	insufficient data	Z: P1.18-1,30-2: F5-7: ST-5953 ()	18-1	30-2	F5-7	11	309
93197	COL201904-38	2019	male	throat swab	E	9852	ST-60 complex	insufficient data	13	24	0	237	cross-reactive	E: P1.5,2: F5-8: ST-9852 (cc60)	5	2	F5-8	3	79
93198	COL201904-39	2019	male	throat swab	B	1946	ST-461 complex	insufficient data	47	118	0	230	cross-reactive	B: P1.19-2,13-1: F3-9: ST-1946 (cc461)	19-2	13-1	F3-9	367	60
93199	COL201904-40	2019	male	throat swab	cnl	53	ST-53 complex	insufficient data	102	58	0	635	insufficient data	cnl: P1.7,30-3: F1-2: ST-53 (cc53)	7	30-3	F1-2	28	13
93200	COL201904-43	2019	male	throat swab	E	1157	ST-1157 complex	insufficient data	24	114	0	2541	insufficient data	E: P1.21-7,16: F5-36: ST-1157 (cc1157)	21-7	16	F5-36	4	47
93201	COL201904-44	2019	male	throat swab	cnl	53	ST-53 complex	insufficient data	102	58	0	637	insufficient data	cnl: P1.7,30: F1-2: ST-53 (cc53)	7	30	F1-2	28	13
93202	COL201904-45	2019	male	throat swab	B	13018		insufficient data	520	8	0		insufficient data	B: P1.19,15: F1-5: ST-13018 ()	19	15	F1-5	12	704
93203	COL201904-46	2019	female	throat swab	cnl	15162	ST-35 complex	insufficient data	24	21	0	4884	insufficient data	cnl: P1.7-2,13-66: F1-7: ST-15162 (cc35)	7-2	13-66	F1-7	4	29
93204	COL201904-49	2019	male	throat swab	E	6119		insufficient data	16		0		cross-reactive	E: P1.12-8,16-179: F5-81: ST-6119 ()	12-8	16-179	F5-81		1525
93205	COL201904-52	2019	male	throat swab	E	6119		insufficient data	16	816	0		cross-reactive	E: P1.12-8,23-6: F5-81: ST-6119 ()	12-8	23-6	F5-81	4	1525
93206	COL201904-56	2019	female	throat swab	cnl	34	ST-32 complex	exact match	1	3	1		cross-reactive	cnl: P1.19,15-8: F5-1: ST-34 (cc32)	19	15-8	F5-1	2	51
93209	COL201904-63	2019	male	throat swab	Z	3882		insufficient data	22	601	0		insufficient data	Z: P1.ND,30-8: F5-7: ST-3882 ()		30-8	F5-7	11	309
93210	COL201904-65	2019	female	throat swab	Z	10866		insufficient data	16		0		cross-reactive	Z: P1.22-4,14-13: F5-7: ST-10866 ()	22-4	14-13	F5-7	11	314
93212	COL201904-83	2019	female	throat swab		23	ST-23 complex	insufficient data	25	7	0	228	cross-reactive	ND: P1.5-2,10-1: F4-1: ST-23 (cc23)	5-2	10-1	F4-1	1	70
93213	COL201905-33	2019	male	throat swab	cnl	2384	ST-198 complex	cross-reactive	4	10	0	1485	cross-reactive	cnl: P1.17,9: F5-5: ST-2384 (cc198)	17	9	F5-5	362	30
189701	COL201802-76	2018	male	urethral swab	C	11	ST-11 complex	insufficient data	973	20	0		insufficient data	C: P1.5-1,10-1: F3-6: ST-11 (cc11)	5-1	10-1	F3-6	4	1
