# Supplementary material for: Genomic Typing of Meningococcal Carriage Isolates in an Urban Sexual Health Clinic
Source: Pathogens. 2026 May 12;15(5):516. doi: 10.3390/pathogens15050516 (PMC13209751; doi:10.3390/pathogens15050516)
Supplement: Supplementary file 1 [file pathogens-15-00516-s001.zip › Table S3.pdf]

Table S3. Dataset of 281 ST-1466*Nm* isolates used in phylogenic analysis of Figure 3

id	isolate	country	year	disease	source	epidemiology	genogroup	PorA_VR1	PorA_VR2	FetA_VR	fHbp peptide	NHBA peptide	NadA peptide	(Bexsero Antigen
20053	M10 240666	UK [England]	2010	invasive (unspecified/other)			W/Y	21	16	F3-7	21	6	8	14
20072	M10 240694	UK [England]	2010	invasive (unspecified/other)			Y	21	16	F3-7	21	18	8	164
20115	M10 240759	UK [England]	2010	invasive (unspecified/other)			Y	21	16	F3-7	21	6	8	14
20232	M11 240073	UK [England]	2011	invasive (unspecified/other)			Y	22	9	F3-7	21	6	8	117
20282	M11 240161	UK [England]	2011	invasive (unspecified/other)			Y	21	16	F3-7	226	6	8	135
20285	M11 240165	UK [England]	2011	invasive (unspecified/other)			Y	21	16	F3-7	254	6	8	72
20308	M11 240209	UK [England]	2011	invasive (unspecified/other)			Y	21	16	F3-7	21	6	8	14
21339	M12 240076	UK [England]	2012	invasive (unspecified/other)			Y	21	16	F3-7	21	6	8	14
21421	M12 240205	UK [England]	2012	invasive (unspecified/other)			Y	21	16	F3-7	21	6	8	14
26129	0759000255	Sweden	2007				Y	22	9	F3-7	21	6	8	117
26292	BB60	UK	2012				Y	21	16	F3-7	21	6	8	14
26872	IE12Nm09	Ireland	2012	invasive (unspecified/other)	blood		Y	21	16	F3-7	21	6	8	14
26902	IE13Nm06	Ireland	2013	invasive (unspecified/other)	joint fluid		Y	18-1	3	F3-7	21	6	8	156
27506	20132	UK [England]	2009				Y	5	2	F3-7	21	6	8	1170
27517	21292	UK [England]	2009				Y	21	16	F3-7	21	6	8	14
27523	21789	UK [England]	2009				Y	22	9	F3-7	21	680	8	127
27526	20951	UK [England]	2009				Y	21	16	F3-7	13	6	8	11
27530	21092	UK [England]	2009				Y	21	16	F3-7	21	6	8	14
27538	21888	UK [England]	2009					21	16	F3-7	13	6	8	11
27539	22007	UK [England]	2009				Y		16	F3-7	21	6	8	
27540	22008	UK [England]	2009				Y	21	16	F3-7	21	6	8	14
27541	22014	UK [England]	2009				Y	21	16	F3-7	21	6	8	14
27556	22933	UK [England]	2009				Y	21	16	F3-7	21	6	8	14
27578	23214	UK [England]	2009				Y	21	16	F3-7	21	6	8	14
27580	23283	UK [England]	2009				Y	21	16	F3-7	21	6	8	14
27581	23326	UK [England]	2009				Y	21	16	F3-7	13	6	8	11
28086	M12 240854	UK [England]	2012	invasive (unspecified/other)			C	21	16	F3-7	13	6	8	11
28087	M12 240855	UK [England]	2012	invasive (unspecified/other)			C	21	16	F3-7	13		8	
28173	M12 240717	UK [England]	2012	invasive (unspecified/other)			Y	22	9	F3-7	21	680	8	127
28180	M12 240784	UK [England]	2012	invasive (unspecified/other)			Y	7-2	13-15	F3-7	21	6	131	177
28247	N241.1	UK			throat swab	carrier	Y	21	16	F3-7	13	6	8	11
28248	N51.1	UK			throat swab	carrier	Y	21	16	F3-7	13	6	8	11
28249	N52.1	UK			throat swab	carrier	Y	21	16	F3-7	13	6	8	11
28251	N58.1	UK			throat swab	carrier	Y	21	16	F3-7	13	6	8	11
28252	N59.1	UK			throat swab	carrier	Y	21	16	F3-7	13	6	8	11
28253	N88.1	UK			throat swab	carrier	Y	21	16	F3-7	13	6	8	11
28254	N138.1	UK			throat swab	carrier	Y	21	16	F3-7	13	6	8	11
28273	N349.1	UK			throat swab	carrier	Y	21	16	F3-7	13	6	8	11
28274	N424.1	UK			throat swab	carrier	Y	21	16-20	F3-7	13	6	8	34
28275	N342.1	UK			throat swab	carrier	Y	21	16-117	F3-7	13	6	8	40
28277	N429.1	UK			throat swab	carrier	Y	21	16-20	F3-7	13	6	8	34
28278	N438.1	UK			throat swab	carrier	Y	21	16-20	F3-7	13	6	8	34
28279	N449.1	UK			throat swab	carrier	Y	21	16-117	F3-7	13	6	8	40
28280	N331.1	UK			throat swab	carrier	Y	21	16-117	F3-7	13	6	8	40
28299	N59.1.1	UK					Y	21	16	F3-7	13	6	8	11
28300	N59.3	UK					Y	21	16	F3-7	13	6	8	11
28301	N59.4	UK					Y	21	16	F3-7	13	6	8	11
28303	N59.6	UK					Y	21	16	F3-7	13	6	8	11
28304	N59.7	UK					Y	21	16	F3-7	13	6	8	11
28305	N59.8	UK					Y	21	16	F3-7	13	6	8	11
28306	N59.9	UK					Y	21	16	F3-7	13	6	8	11
28307	N59.10	UK					Y	21	16	F3-7	13	6	8	11
28308	N59.11	UK					Y	21	16	F3-7	13	6	8	11
28309	N253.1	UK					Y	21	16	F3-7	13	6	8	11
28310	N253.2	UK					Y	21	16	F3-7	13	6	8	11
28311	N253.3	UK					Y	21	16	F3-7	13	6	8	11
28312	N253.4 (1)	UK					Y	21	16	F3-7	13	6	8	11
28313	N253.5	UK					Y	21	16	F3-7	13	6	8	11
28314	N253.6	UK					Y	21	16	F3-7	13	6	8	11
28315	N253.7	UK					Y	21	16	F3-7	13	6	8	11

28316	N253.8	UK					Y	21	16	F3-7	13	6	8	11
28317	N253.9	UK					Y	21	16	F3-7	13	6	8	11
28318	N253.10	UK					Y	21	16	F3-7	13	6	8	11
28319	N352.1	UK					Y	21	16	F3-7	13	6	8	11
28320	N352.2	UK					Y	21	16	F3-7	13	6	8	11
28321	N352.3	UK					Y	21	16	F3-7	13	6	8	11
28322	N352.4	UK					Y	21	16	F3-7	13	6	8	11
28323	N352.5 (1)	UK					Y	21	16	F3-7	13	6	8	11
28324	N352.6	UK					Y	21	16	F3-7	13	6	8	11
28325	N352.7	UK					Y	21	16	F3-7	13	6	8	11
28326	N352.8	UK					Y	21	16	F3-7	13	6	8	11
28327	N352.9	UK					Y	21	16	F3-7	13	6	8	11
28328	N352.10	UK					Y	21	16	F3-7	13	6	8	11
28329	N438.1.1	UK					Y	21	16	F3-7	13	6	8	11
28330	N438.2	UK					Y	21	16	F3-7	13	6	8	11
28331	N438.3	UK					Y	21	16	F3-7	13	6	8	11
28332	N438.4	UK					Y	21	16	F3-7	13	6	8	11
28333	N438.5	UK					Y	21	16	F3-7	13	6	8	11
28334	N438.6	UK					Y	21	16	F3-7	13	6	8	11
28335	N438.7	UK					Y	21	16	F3-7	13	6	8	11
28336	N438.8	UK					Y	21	16	F3-7	13	6	8	11
28337	N438.9	UK					Y	21	16	F3-7	13	6	8	11
28338	N438.10	UK					Y	21	16	F3-7	13	6	8	11
29148	IBD-819	The Netherlands	2012	invasive (unspecified/other)	CSF		Y	21	16-47	F3-7	21	6	8	1166
29149	IBD-820	The Netherlands	2012	invasive (unspecified/other)	blood		Y	21	16-47	F3-7	21	6	8	1166
29153	IBD-824	The Netherlands	2012	invasive (unspecified/other)	CSF		Y	21	16	F3-7	21	6	0	1171
30559	IBD-954	Germany	2011	invasive (unspecified/other)	blood		Y	22	9	F3-7	21	6	8	117
34921	IBD-852	The Netherlands	2011	invasive (unspecified/other)	blood		Y	21	16	F3-7	21	6	8	14
35268	09.3302.K	UK [Scotland]	2009	invasive (unspecified/other)	blood		Y	21	16	F3-7	21	6	8	14
36143	12018_2014	Ireland	2014	invasive (unspecified/other)			Y	18-1	3	F3-7	21	6	8	156
36151	12026_2014	Ireland	2014	invasive (unspecified/other)			Y	21	16	F3-7	13	6	8	11
37729	M14 240571	UK [England]	2014	invasive (unspecified/other)			Y	21	16	F3-7	21	6	8	14
37863	M15 240094	UK [England]	2015	invasive (unspecified/other)				21	16	F3-7	21	6	8	14
38129	M15 240033	UK [England]	2015	invasive (unspecified/other)				21	16	F3-7	21	6	8	14
39377	M15 240750	UK [England]	2015	invasive (unspecified/other)			Y	21	16	F3-7	21	6	8	14
40249	LNP28316	France	2015	invasive (unspecified/other)			NG	21	16	F3-7	21	6	8	14
41551	M15 240987	UK [England]	2015	invasive (unspecified/other)			Y	21	16	F3-7	21	6	8	14
41745	LNP27890	France	2014	invasive (unspecified/other)			Y	21	16	F3-7	21	6	8	14
41750	LNP27945	France	2015	invasive (unspecified/other)			Y	21	16	F3-7	21	6	8	14
42128	IPM20	Morocco	2013	invasive (unspecified/other)			Y	21		F3-7	21	6	8	
46519	4449	Tunisia	2013	invasive (unspecified/other)			NG	22-1	14	F3-7	21	6	8	
46531	6214	Tunisia	2015				NG	22	9	F3-7	21	680	8	127
46678	GL42229	UK [Scotland]	2015	carrier	throat swab	carrier	Y	21	16	F3-7	21	6	8	14
49696	LO40728	UK [England]	2015	carrier	throat swab	carrier	Y	21	16	F3-7	21	6	8	14
50111	WG40014	UK [England]	2015	carrier	throat swab	carrier	Y	21	16	F3-7	21	6	8	14
50112	WG40017	UK [England]	2015	carrier	throat swab	carrier	Y	21	16	F3-7	21	6	8	14
50213	WG41382	UK [England]	2015	carrier	throat swab	carrier	Y	5-1	2-2	F2-16	21	6	8	2020
50734	M40267	USA	2016				Y	21	16	F3-7	21	6	8	14
52864	16.8705113	UK [Scotland]	2016	invasive (unspecified/other)	blood		Y	21	16	F3-7	21	6	8	14
53889	N51.2	UK [England]	2008	carrier	throat swab	carrier	Y	21	16	F3-7	13	6	8	11
53890	N51.3	UK [England]	2008	carrier	throat swab	carrier	Y	21	16	F3-7	13	6	8	11
53891	N51.4	UK [England]	2008	carrier	throat swab	carrier	Y	21	16	F3-7	13	6	8	11
53892	N51.5	UK [England]	2008	carrier	throat swab	carrier	Y	21	16	F3-7	13	6	8	11
53893	N51.6	UK [England]	2008	carrier	throat swab	carrier	Y	21	16	F3-7	13	6	8	11
53894	N236.1 (1)	UK [England]	2008	carrier	throat swab	carrier	Y	21	16	F3-7	13	6	8	11
53895	N236.2	UK [England]	2008	carrier	throat swab	carrier	Y	21	16	F3-7	13	6	8	11
53896	N236.3	UK [England]	2008	carrier	throat swab	carrier	Y	21	16	F3-7	13	6	8	11
53897	N236.4	UK [England]	2008	carrier	throat swab	carrier	Y	21	16	F3-7	13	6	8	11
53898	N236.5	UK [England]	2008	carrier	throat swab	carrier	Y	21	16	F3-7	13	6	8	11
53899	N236.6	UK [England]	2008	carrier	throat swab	carrier	Y	21	16	F3-7	13	6	8	11
53900	N354.1	UK [England]	2009	carrier	throat swab	carrier	Y	21	16	F3-7	13	6	8	11
53901	N354.2	UK [England]	2009	carrier	throat swab	carrier	Y	21	16	F3-7	13	6	8	11
53902	N354.3	UK [England]	2009	carrier	throat swab	carrier	Y	21	16	F3-7	13	6	8	11
53903	N354.4	UK [England]	2009	carrier	throat swab	carrier	Y	21	16	F3-7	13	6	8	11

53904	N354.5	UK [England]	2009	carrier	throat swab	carrier	Y	21	16	F3-7	13	6	8	11
53905	N354.6	UK [England]	2009	carrier	throat swab	carrier	Y	21	16	F3-7	13	6	8	11
53906	N424.2	UK [England]	2009	carrier	throat swab	carrier	Y	21	16	F3-7	13	6	8	11
53907	N424.3	UK [England]	2009	carrier	throat swab	carrier	Y	21	16	F3-7	13	6	8	11
53908	N424.4	UK [England]	2009	carrier	throat swab	carrier	Y	21	16	F3-7	13	6	8	11
53909	N424.5	UK [England]	2009	carrier	throat swab	carrier	Y	21	16	F3-7	13	6	8	11
53910	N424.6	UK [England]	2009	carrier	throat swab	carrier	Y	21	16	F3-7	13	6	8	11
54461	N329	UK [England]	2009	carrier	throat swab	carrier	Y	21	16	F3-7	13	6	8	11
54596	LNP20098abd	France	2002	invasive (unspecified/other)				21	16	F3-7	21	6	8	14
60721	M18 240097	UK [England]	2018	invasive (unspecified/other)			Y	21	16	F3-7	21	6	8	14
61179	NmissMEN44	Italy	2016	carrier			Y	21	16	F3-7	21	6		
63645	M18 240563	UK [England]	2018	invasive (unspecified/other)			Y	21	16	F3-7	21	6	8	14
71470	NZ14MI0032	New Zealand	2014	invasive (unspecified/other)	CSF		Y	21	16	F3-7	21	6	8	14
71517	NZ16MI0008	New Zealand	2016	invasive (unspecified/other)	blood		Y	21	16	F3-7	21	6	8	14
72094	COL201907-31	USA	2019		throat swab	carrier	Y	21	16	F3-7	21	6	8	14
72230	COL201807-50	USA	2018		throat swab	carrier		21	16	F3-7	21	6	8	14
72312	M19 240221	UK [England]	2019	carrier				21	16	F3-7	21	6	8	14
72654	SMG-20-387	UK [Scotland]	2020	invasive (unspecified/other)	blood		Y	21	16	F3-7	21	6	8	14
72708	COL201912-53	USA	2019		throat swab	carrier		21	16	F3-7	21	6	8	14
72716	COL201912-81	USA	2019		throat swab	carrier	Y	21	16	F3-7	21	6	8	14
75076	DE14539	Germany	2019	invasive (unspecified/other)	blood	sporadic case	Y	21	16	F3-7	21	6	192	4251
79044	SMG-20-1091 (2b	UK [Scotland]	2016	other	throat swab		Y	21	16	F3-7	21	6	8	14
79966	M19 240543	UK [England]	2019	invasive (unspecified/other)			Y	21	16	F3-7	21	6	192	4251
82068	0061/18 (2b)	Czech Republic	2018	invasive (unspecified/other)	blood	sporadic case	Y	21	16	F3-7	21	6	8	14
82069	0062/18	Czech Republic	2018	invasive (unspecified/other)	throat swab	sporadic case	Y	21	16	F3-7	21	6	8	14
85388	1660355	Spain	2018		urethral swab	sporadic case	Y	21	16	F3-7	21	6	8	14
85585	M18 240331	UK [England]	2018	invasive (unspecified/other)			Y	21	16	F3-7	13	6	8	11
88171	DE14408	Germany	2019	invasive (unspecified/other)	blood	sporadic case		21	16	F3-7	21	6	8	14
90284	MB35196	Australia	2017	carrier	throat swab	carrier	Y	21	16	F3-7	21	6	8	14
90581	MB50552	Australia	2018	carrier	throat swab	carrier	Y	21	16	F3-7	21	6	8	14
93015	COL201807-38	USA	2018		throat swab	carrier		21	16	F1-2;F3-7	21	6	8	14
93141	COL201901-31	USA	2019		throat swab	carrier		21	16	F3-7	21	6	8	14
93192	COL201903-66	USA	2019		throat swab	carrier		21	16	F3-7	21	6	8	14
93651	DE14460	Germany	2019	septicaemia	blood	sporadic case	Y	21	16	F3-7	21	1639	8	
96319	M46104	USA	2017	invasive (unspecified/other)			Y	21	16	F3-7	21	6	8	14
96381	M44533	USA	2016	invasive (unspecified/other)			Y	21	16	F3-7	21	6	8	14
96427	M43294	USA	2017	invasive (unspecified/other)			Y	21	16	F3-7	21	6	8	14
96519	M41675	USA	2017	invasive (unspecified/other)			Y	21	16	F3-7	21	6	8	14
96676	M40282	USA	2016	invasive (unspecified/other)			Y	21	16	F3-7	21	6	163	
96679	M40271	USA	2016	invasive (unspecified/other)			Y	21	16	F3-7	21	6	8	14
96795	M39195	USA	2016	invasive (unspecified/other)			Y	21	16	F3-7	21	6	8	14
96796	M39194	USA	2016	invasive (unspecified/other)			Y	21	16	F3-7	21	6	8	14
96996	M37350	USA	2015	invasive (unspecified/other)			Y	21	16	F3-7	21	6	8	14
97188	M28978	USA	2013	invasive (unspecified/other)			Y	21	16	F3-7	21	6	8	14
97190	M28976	USA	2013	invasive (unspecified/other)			Y	21	16	F3-7	21	6	8	14
97298	M27478	USA	2013	invasive (unspecified/other)			Y	21	16	F3-7	21	6	8	14
100425	2020193	The Netherlands	2002				Y	21	16	F3-7	21	6	8	14
100473	2842STDY588122[illegible]	The Netherlands					Y	21	16	F3-7	21	6	8	14
100559	2842STDY588131[illegible]	The Netherlands					Y	21	16-47	F3-7	21	6	8	1166
100560	2120265	The Netherlands	2012				Y	21	16	F3-7	21	6	0	1171
100964	2070189	The Netherlands	2007				Y	21	16	F3-7	21	6	8	14
100997	2120194	The Netherlands	2012				Y	21	16-47	F3-7	21	6	8	1166
101080	2070189_II	The Netherlands					Y	21	16	F3-7	21	6	8	14
101113	2120194_II	The Netherlands					Y	21	16-47	F3-7	21	6	8	1166
115261	NM000028	USA	2019	meningitis and septicaemia			Y	21	16	F3-7	21	6	8	14
115290	NM000060	USA	2019	carrier			Y	21	16	F3-7	21	6	8	14
115344	M21 240022	UK [England]	2021	invasive (unspecified/other)			Y	21	16	F3-7	21	6	192	4251
117583	M21 240039	UK [England]	2021	invasive (unspecified/other)			Y	21	16	F3-7	21	6	8	14
124947	M53855	USA	2019	invasive (unspecified/other)	blood		Y	21	16	F3-7	21	6	8	14
125020	M52747	USA	2019	invasive (unspecified/other)	blood		Y	21	16	F3-7	21	6	8	14
126232	NML2017-010	Canada	2017				Y	21	16	F3-7	21	6	8	14
127029	M47266	USA	2018	invasive (unspecified/other)	blood		Y	21	16	F3-7	21	6	8	14
127190	M50715	USA	2019	invasive (unspecified/other)	blood		Y	21	16	F3-7	21	6	8	14
127338	H04-RESP-006	Spain	2022	other	throat swab	sporadic case	Y	21	16	F3-7	21	6	192	4251

127720	H09-RESP-006	Spain	2013	other	throat swab	sporadic case	Y	21	16	F3-7	21	6	8	14
128269	EPD012	Spain	2022	carrier	throat swab		Y	21	16	F3-7	21	6	8	14
128277	Nmen312	Spain	2021	carrier	throat swab		Y	21	16	F3-14	21	6	192	4251
133408	NML2018-011	Canada	2018				Y	21	16	F3-7	21	6	8	14
135376	H02-EMI-003	Spain	2015	invasive (unspecified/other)	blood		C	21	16-2	F3-7	21	6	8	3899
140837	H03-RESP-023	Spain	2023	other	throat swab		Y	21	16	F3-7	21	6	8	14
140843	H07-RESP-027	Spain	2023	carrier	throat swab		Y	21	16	F3-7	21	6		
140992	M58903	USA	2022	invasive (unspecified/other)	blood		Y	21	16	F3-7	21	6	8	14
140993	M58886	USA	2022	invasive (unspecified/other)	blood		Y	21	16	F3-7	21	6	192	4251
140994	M58851	USA	2022	invasive (unspecified/other)	blood		Y	21	16	F3-7	21	6	192	4251
140995	M58496	USA	2022	invasive (unspecified/other)	blood		Y	21	16	F3-7	21	6	192	4251
140996	M58488	USA	2022	invasive (unspecified/other)	blood		Y	21	16	F3-7	21	6	192	4251
140997	M58434 (2a)	USA	2022	invasive (unspecified/other)	blood		Y	21	16	F3-7	21	6	192	4251
140998	M58397	USA	2022	invasive (unspecified/other)	blood		Y	21	16	F3-7	21	6	192	4251
140999	M58393	USA	2022	invasive (unspecified/other)	blood		Y	21	16	F3-7	21	6	192	4251
141000	M58392	USA	2022	invasive (unspecified/other)	blood		Y	21	16	F3-7	21	6		
141001	M58368	USA	2022	invasive (unspecified/other)	blood		Y	21	16	F3-7	21	6	192	4251
141002	M58303	USA	2022	invasive (unspecified/other)	blood		Y	21	16	F3-7	21	6	192	4251
141003	M58295	USA	2022	invasive (unspecified/other)	blood		Y	21	16	F3-7	21	6	192	4251
141004	M58280	USA	2022	invasive (unspecified/other)	blood		Y	21	16	F3-7	21	6	192	4251
141005	M58248	USA	2022	invasive (unspecified/other)	blood		Y	21	16	F3-7	31	6	8	
141006	M58231	USA	2022	invasive (unspecified/other)	joint fluid		Y	21	16	F3-7	21	6	8	14
141007	M58173	USA	2022	invasive (unspecified/other)	blood		Y	21	16	F3-7	21	6	192	4251
141008	M58128	USA	2022	invasive (unspecified/other)	blood		Y	21	16		21	6	192	4251
141009	M58127	USA	2022	invasive (unspecified/other)	blood		Y	21	16	F3-7	21	6	192	4251
141010	M58102	USA	2022	invasive (unspecified/other)	blood		Y	21	16	F3-7	21	6	192	4251
141011	M58101	USA	2022	invasive (unspecified/other)	blood		Y	21	16	F3-7	21	6	192	4251
141012	M58100	USA	2022	invasive (unspecified/other)	blood		Y	21	16	F3-7	21	6	192	4251
141013	M58095	USA	2022	invasive (unspecified/other)	blood		Y	18-1	34	F3-7	21	6	8	
141014	M58045	USA	2022	invasive (unspecified/other)	blood		Y	21	16	F3-7	21	6	192	4251
141015	M57649	USA	2022	invasive (unspecified/other)	blood		Y	21	16	F3-7	21	6	192	4251
141016	M57352	USA	2022	invasive (unspecified/other)	blood		Y	21	16	F3-7	21	6	8	14
141057	M57512	USA	2022	invasive (unspecified/other)	blood		Y	21	16	F3-7	21	6	192	4251
141058	M57637	USA	2022	invasive (unspecified/other)	blood		Y	21	16	F3-7	21	6	192	4251
141059	M58096	USA	2022	invasive (unspecified/other)	blood		Y	21	16	F3-7	21	6	192	4251
141207	NML2023-108	Canada	2023				Y	21	16	F3-7	21	6	192	4251
141213	NML2023-116	Canada	2023				Y	21	16	F3-7	21	6	192	4251
143368	2332610583	Australia	2023				Y	21	16	F3-7	21	6	224	4742
143369	2334610973	Australia	2023				Y	21	16	F3-7	21	6	192	4251
143428	NML2023-135	Canada	2023				Y	21	16	F3-7	21	6	192	4251
144239	NML2023-144	Canada	2023				Y	21	16	F3-7	21	6	192	4251
150046	H07-RESP-036	Spain	2024	carrier	throat swab		Y	21	16	F3-7	21	20	8	4894
150048	H01-GEN-007	Spain	2024	meningococcal STI	urethral swab		Y	21	16	F3-7	21	6	8	14
150365	NML2024-059	Canada	2024				Y	21	16	F3-7	21	6	192	4251
150592	SMG-24-1523	UK [Scotland]	2024	other	urethral swab		Y	21	16	F3-7	21	6	192	4251
151635	H01-GEN-009	Spain	2024	meningococcal STI	urethral swab		Y	21	16	F3-7	21	6	192	4251
151644	H01-RESP-069	Spain	2023	carrier	throat swab		Y	21	16	F3-7	21	6	8	14
153700	H01-RESP-129	Spain	2024	carrier	throat swab		Y	21	16	F3-7	21	6	192	4251
154840	H01-RESP-155	Spain	2024	carrier	throat swab		Y	21	16	F3-7	21	6	192	4251
160960	SMG-24-2701	UK [Scotland]	2024	other	rectal swab		Y	21	16	F3-7	21	6	192	4251
161154	SMG-24-2748	UK [Scotland]	2024	other	female reproductive tract		Y	21	16	F3-7	21	6	192	4251
162369	Nm531	China	2024	meningococcal STI	urethral swab	sporadic case	Y	21	16	F3-7	21	6	192	4251
162448	23MN0320	UK [England]	2023	invasive (unspecified/other)			Y	21	16	F3-7	218	6	8	
162580	NML2024-146	Canada	2024				Y	21	16	F3-7	21	6		
165430	19R218539T	Australia	2019	meningococcal STI	urethral swab	sporadic case	Y	21	16	F3-7	21	6	8	14
165431	23K349289O	Australia	2023	meningococcal STI	urethral swab	endemic	Y	21	16	F3-7	21	6	192	4251
165432	23K352085C	Australia	2023	meningococcal STI	urethral swab	endemic	Y	21	16	F3-7	21	6		
165433	23R368825S-2	Australia	2023	meningococcal STI	urethral swab	endemic	Y	21	16	F3-7	21	6	192	4251
165434	23R540670I	Australia	2023	meningococcal STI	urethral swab	endemic	Y	21	16	F3-7	21	6	192	4251
165435	23R547202G	Australia	2023	meningococcal STI	urethral swab	endemic	Y	21	16	F3-7	21	6	192	4251
165436	23R555312H	Australia	2023	meningococcal STI	urethral swab	endemic	Y	21	16	F3-7	21	6	8	14
165437	23R557728U	Australia	2023	meningococcal STI	urethral swab	endemic	Y	21	16	F3-7	21	6	192	4251
165438	23R574398W	Australia	2023	meningococcal STI	urethral swab	endemic	Y	21	16	F3-7	21	6	192	4251
165439	23R580293N	Australia	2023	meningococcal STI	urethral swab	endemic	Y	21	16	F3-7	21	6	192	4251

165440	23R584430K	Australia	2023	meningococcal STI	urethral swab	endemic	Y	21	16	F3-7	21	6	192	4251
165441	23R591467S	Australia	2023	meningococcal STI	urethral swab	endemic	Y	21	16	F3-7	21	6	192	4251
165442	23R599334T	Australia	2023	meningococcal STI	urethral swab	endemic	Y	21	16	F3-7	21	6	192	4251
165443	23R619294R	Australia	2023	meningococcal STI	urethral swab	endemic	Y	21	16	F3-7	21	6	192	4251
165444	23R625075L	Australia	2023	meningococcal STI	urethral swab	endemic	Y	21	16	F3-7	21	6	192	4251
165445	23R625264L	Australia	2023	meningococcal STI	urethral swab	endemic	Y	21	16	F3-7	21	6	192	4251
165446	23R6310208	Australia	2023	meningococcal STI	urethral swab	endemic	Y	21	16	F3-7	21	6	192	4251
165447	23R645016I	Australia	2023	meningococcal STI	urethral swab	endemic	Y	21	16	F3-7	21	6	192	4251
165448	23R649595Y	Australia	2023	meningococcal STI	urethral swab	endemic	Y	21	16	F3-7	21	6	192	4251
165449	23R652186O	Australia	2023	meningococcal STI	urethral swab	endemic	Y	21	16	F3-7	21	6	192	4251
165450	23R652187P	Australia	2023	meningococcal STI	urethral swab	endemic	Y	21	16	F3-7	21	6	192	4251
165451	23R653919T	Australia	2023	meningococcal STI	urethral swab	endemic	Y	21	16	F3-7	21	6	192	4251
165452	23R653941O	Australia	2023	meningococcal STI	urethral swab	endemic	Y	21	16	F3-7	21	6	192	4251
165453	23R658162O	Australia	2023	meningococcal STI	urethral swab	endemic	Y	21	16	F3-7	21	6	192	4251
165454	23R673266Q	Australia	2023	meningococcal STI	urethral swab	endemic	Y	21	16	F3-7	21	6	192	4251
165455	23R681714N	Australia	2023	meningococcal STI	urethral swab	endemic	Y	21	16	F3-7	21	6	192	4251
165456	23R681788Y	Australia	2023	meningococcal STI	urethral swab	endemic	Y	21	16	F3-7	21	6	192	4251
165457	23R681793U	Australia	2023	meningococcal STI	urethral swab	endemic	Y	21	16	F3-7	21	6	192	4251
165458	23R689450S	Australia	2023	meningococcal STI	urethral swab	endemic	Y	21	16	F3-7	21	6	192	4251
165459	23R6894591	Australia	2023	meningococcal STI	urethral swab	endemic	Y	21	16	F3-7	21	6	192	4251
165460	23R692596X	Australia	2023	meningococcal STI	urethral swab	endemic	Y	21	16	F3-7	21	6	192	4251
166546	NZ24MI0028	New Zealand	2024		female reproductive tract		Y	21	16	F3-7	21	6	192	4251
167446	NML2025-024	Canada	2025				Y	21	16	F3-7	21	6		
167633	S16BD02367	Belgium	2016	invasive (unspecified/other)	blood		Y	21	16	F3-7	21	6	8	14
168407	SMG-25-858	UK [Scotland]	2025	other	urethral swab		Y	21	16	F3-7	21	6	192	4251
169073	NML2025-066	Canada	2025				Y	21	16	F3-7	21	6	192	4251
169087	NML2025-083	Canada	2025				Y	21	16	F3-7	21	6	192	4251
170006	SMG-25-1444	UK [Scotland]	2025	other	urethral swab		Y	21	16	F3-7	21	6	192	4251
178721	CD250911	China	2025	meningococcal STI	urethral swab	sporadic case	Y							
