# Supplementary material for: Genomic Typing of Meningococcal Carriage Isolates in an Urban Sexual Health Clinic
Source: Pathogens. 2026 May 12;15(5):516. doi: 10.3390/pathogens15050516 (PMC13209751; doi:10.3390/pathogens15050516)
Supplement: Supplementary file 1 [file pathogens-15-00516-s001.zip › Table S4.pdf]

Table S4. Genomic contig statistics summary

PubMLST ID	isolate	year	source	Contig #	Total length	Minimal length	Maximum length	Mean length	N50	L50	N90	L90	N95	L95	Note
71954	COL201804-31	2018	throat swab	279	2,079,794	253	81,575	7,455	16,256	40	4,831	134	2,896	162	
72044	COL201804-48	2018	throat swab	1	2,161,085										
72045	COL201904-29	2019	throat swab	446	2,153,458	100	113,561	4,829	26,645	24	6,039	87	2,742	113	
72046	COL201904-31	2019	throat swab	420	2,080,747	100	109,773	4,955	25,008	26	7,656	81	3,388	101	
72047	COL201904-36	2019	throat swab	464	2,092,072	100	86,340	4,509	24,989	27	6,102	87	2,634	111	
72048	COL201904-51	2019	throat swab	521	2,184,351	100	89,284	4,193	27,037	26	6,186	95	2,038	123	
72049	COL201904-84	2019	throat swab	159	2,166,782	301	156,073	13,628	46,694	15	10,034	50	4,625	66	
72050	COL201904-87	2019	throat swab	725	2,322,626	100	67,503	3,204	26,155	30	3,349	115	527	215	
72051	COL201905-27	2019	throat swab	156	2,043,836	251	93,481	13,102	26,247	24	9,157	73	4,615	87	
72052	COL201905-29	2019	throat swab	583	2,289,627	100	94,209	3,928	20,503	36	4,098	126	1,759	168	
72053	COL201905-30	2019	throat swab	405	2,080,590	100	87,183	5,138	26,526	28	7,359	82	3,899	101	
72054	COL201905-35	2019	throat swab	141	2,019,591	322	140,027	14,324	29,362	19	9,098	64	5,562	78	
72055	COL201905-36	2019	throat swab	409	2,096,681	100	111,655	5,127	24,455	26	7,252	83	3,771	103	
72057	COL201905-40	2019	throat swab	522	2,168,184	100	80,669	4,154	24,702	28	4,934	100	1,946	135	
72058	COL201905-41	2019	throat swab	852	2,165,878	100	120,673	2,543	21,045	30	3,006	116	275	304	
72060	COL201905-45	2019	throat swab	452	2,182,692	100	99,588	4,829	27,805	24	5,947	93	2,662	120	
72061	COL201905-47	2019	throat swab	474	2,154,265	100	113,546	4,545	25,643	25	6,039	87	2,790	112	
72062	COL201905-48	2019	throat swab	476	2,087,470	100	80,288	4,386	24,197	25	5,943	92	2,702	117	
72063	COL201905-50	2019	throat swab	3469	3,256,119	100	104,968	939	9,968	71	288	2,036	250	2,644	Contains additional DNA from Capnocytophaga sp.
72064	COL201905-51	2019	throat swab	6386	4,610,666	100	66,949	722	935	308	279	4,453	252	5,323	Contains additional DNA from Chryseobacterium sp.
72065	COL201905-63	2019	throat swab	467	2,169,497	100	98,286	4,646	27,429	25	6,065	87	2,991	111	
72066	COL201905-64	2019	throat swab	556	2,251,581	100	151,116	4,050	20,847	31	3,948	115	1,662	155	
72067	COL201905-65	2019	throat swab	408	2,103,120	100	111,696	5,155	23,089	27	7,414	86	3,457	107	
72068	COL201906-26	2019	throat swab	418	2,113,039	100	81,062	5,056	26,209	26	6,778	82	2,976	104	
72069	COL201906-27	2019	throat swab	460	2,216,727	100	78,096	4,819	21,407	31	5,917	109	2,614	136	
72070	COL201906-29	2019	throat swab	430	2,081,572	100	100,307	4,841	23,064	27	5,817	94	2,967	119	
72071	COL201906-31	2019	urethral swab	2109	2,824,979	100	68,558	1,340	14,545	48	316	927	258	1,427	Contains additional DNA from Granulicatella sp.
72072	COL201906-34	2019	throat swab	492	2,232,218	100	133,501	4,538	22,568	31	4,429	109	1,940	143	
72073	COL201906-37	2019	throat swab	497	2,095,470	100	78,520	4,217	24,011	26	5,858	92	2,604	117	
72074	COL201906-39	2019	throat swab	570	2,235,409	100	80,276	3,922	23,666	31	4,950	104	2,020	140	
72075	COL201906-40	2019	throat swab	552	2,196,923	100	87,927	3,980	23,667	29	4,920	100	1,399	139	
72076	COL201906-41	2019	throat swab	482	2,088,656	100	78,670	4,334	23,655	27	5,920	92	2,754	117	
72077	COL201906-43	2019	throat swab	552	2,140,227	100	89,877	3,878	22,409	30	5,918	96	2,254	124	
72078	COL201906-44	2019	throat swab	455	2,125,610	100	143,674	4,672	25,710	23	6,065	89	2,505	113	
72079	COL201906-54	2019	throat swab	820	2,367,078	100	74,121	2,887	23,602	32	3,050	125	434	239	
72080	COL201906-55	2019	throat swab	543	2,234,638	100	87,954	4,116	22,230	29	4,636	107	1,923	144	
72081	COL201906-60	2019	throat swab	455	2,165,176	100	106,867	4,759	25,239	26	5,748	91	2,585	118	
72082	COL201906-61	2019	throat swab	591	2,171,301	100	70,816	3,674	18,992	37	4,018	126	1,748	164	
72083	COL201906-64	2019	throat swab	528	2,254,607	100	66,802	4,271	22,285	32	4,526	116	1,820	154	
72084	COL201906-65	2019	throat swab	441	2,329,190	100	99,482	5,282	34,995	20	6,316	85	2,786	110	
72085	COL201906-66	2019	throat swab	463	2,164,685	100	99,593	4,676	27,320	24	5,753	87	2,291	116	
72086	COL201906-67	2019	throat swab	501	2,263,691	100	71,653	4,519	21,406	33	5,126	110	2,160	142	
72087	COL201906-68	2019	throat swab	455	2,121,726	100	86,192	4,664	25,209	26	6,437	85	2,716	110	
72088	COL201906-69	2019	throat swab	424	2,110,341	100	86,190	4,978	25,839	26	6,778	81	2,984	103	
72089	COL201907-26	2019	throat swab	511	2,227,597	100	144,194	4,360	28,640	23	5,459	90	1,751	124	
72090	COL201907-27	2019	throat swab	243	2,089,389	304	77,853	8,599	21,434	30	5,285	107	2,672	136	
72091	COL201907-28	2019	rectal swab	583	2,297,422	100	67,760	3,941	24,398	30	4,710	113	1,659	151	
72092	COL201907-29	2019	throat swab	482	2,153,742	100	113,556	4,469	23,828	25	5,695	94	2,076	122	
72093	COL201907-30	2019	throat swab	607	2,291,913	100	58,958	3,776	20,186	38	4,108	123	1,564	165	
72094	COL201907-31	2019	throat swab	469	2,137,316	100	79,237	4,558	25,737	25	6,074	97	3,292	120	
72095	COL201907-34	2019	throat swab	629	2,169,834	100	70,814	3,450	20,856	33	4,290	114	1,670	152	
72096	COL201907-37	2019	throat swab	445	2,087,357	100	80,678	4,691	25,422	25	6,337	87	3,336	108	
72097	COL201907-40	2019	throat swab	464	2,152,442	100	113,521	4,639	21,856	27	5,982	98	2,690	125	
72098	COL201907-41	2019	throat swab	507	2,107,971	100	100,980	4,158	23,253	27	6,024	91	2,635	114	
72099	COL201907-43	2019	other	448	2,150,116	100	113,521	4,800	26,645	24	6,039	86	2,929	111	
72100	COL201907-44	2019	throat swab	209	2,124,753	314	139,660	10,167	22,068	29	5,975	99	3,917	120	

72101	COL201907-45	2019	throat swab	515	2,157,756	100	77,269	4,190	23,912	27	5,937	94	2,262	122	
72102	COL201907-46	2019	throat swab	476	2,079,766	100	71,052	4,370	23,672	29	5,592	97	2,225	124	
72103	COL201907-48	2019	throat swab	480	2,069,179	100	94,413	4,311	21,045	30	5,394	102	2,498	128	
72104	COL201907-49	2019	throat swab	526	2,249,435	100	88,582	4,277	20,535	34	4,527	120	1,704	158	
72105	COL201907-51	2019	throat swab	476	2,087,322	100	78,156	4,386	23,982	28	6,067	91	2,842	115	
72106	COL201907-52	2019	urethral swab	456	2,150,890	100	113,506	4,717	27,205	24	6,039	86	2,937	112	
72107	COL201907-53	2019	urethral swab	525	2,085,940	100	107,698	3,974	25,377	23	5,335	88	1,985	119	
72108	COL201907-55	2019	throat swab	467	2,091,631	100	96,208	4,479	23,156	28	6,067	95	2,699	119	
72109	COL201907-58	2019	throat swab	535	2,092,236	100	71,405	3,911	21,992	29	4,594	106	1,436	140	
72110	COL201907-59	2019	throat swab	410	2,102,179	100	111,701	5,128	24,262	26	7,687	82	3,936	102	
72111	COL201907-62	2019	throat swab	558	2,274,902	100	91,768	4,077	18,195	33	5,208	122	1,864	155	
72112	COL201907-63	2019	throat swab	536	2,126,217	100	112,760	3,967	26,327	26	5,524	92	2,624	118	
72113	COL201908-27	2019	throat swab	559	2,236,285	100	112,721	4,001	26,580	25	4,020	111	1,366	156	
72114	COL201908-28	2019	throat swab	653	2,316,281	100	94,905	3,548	21,141	29	4,211	114	1,354	156	
72115	COL201908-30	2019	throat swab	451	2,113,440	101	86,367	4,687	21,984	29	7,927	92	3,134	112	
72116	COL201908-31	2019	throat swab	585	2,153,147	100	63,756	3,681	19,072	36	4,303	123	2,107	158	
72117	COL201908-33	2019	throat swab	573	2,155,305	100	108,656	3,762	20,291	32	4,663	117	1,364	154	
72118	COL201908-34	2019	throat swab	220	2,282,923	307	121,716	10,377	28,234	21	6,820	78	3,845	100	
72119	COL201908-37	2019	throat swab	545	2,231,010	100	70,598	4,094	22,409	29	4,508	104	1,841	142	
72120	COL201908-38	2019	throat swab	480	2,173,289	100	99,593	4,528	25,530	25	5,973	94	2,662	121	
72121	COL201908-41	2019	throat swab	453	2,199,997	100	89,690	4,857	24,574	27	6,535	93	2,526	120	
72122	COL201908-43	2019	throat swab	678	2,367,275	100	64,653	3,492	21,035	33	4,487	117	1,223	160	
72123	COL201908-44	2019	throat swab	454	2,086,680	100	78,241	4,597	25,661	26	6,378	88	3,363	109	
72124	COL201908-47	2019	throat swab	523	2,119,590	100	107,366	4,053	22,223	31	5,179	101	1,870	130	
72125	COL201908-48	2019	throat swab	121	2,097,759	309	157,188	17,337	50,869	13	12,091	41	8,077	51	
72126	COL201908-58	2019	throat swab	494	2,221,213	100	79,394	4,497	25,128	24	5,396	95	2,662	124	
72127	COL201908-62	2019	throat swab	481	2,070,928	100	120,627	4,306	22,395	27	5,481	94	2,498	121	
72128	COL201908-64	2019	throat swab	430	2,106,143	100	111,701	4,899	23,089	26	6,098	89	2,956	113	
72129	COL201908-72	2019	throat swab	451	2,104,736	100	86,220	4,667	25,068	27	5,545	88	2,613	115	
72130	COL201908-75	2019	throat swab	595	2,216,637	100	128,940	3,726	20,918	30	4,415	112	1,393	151	
72131	COL201908-79	2019	throat swab	659	2,287,435	100	86,152	3,472	19,052	36	4,214	131	1,396	174	
72132	COL201908-81	2019	throat swab	576	2,198,737	100	73,710	3,818	19,777	36	4,731	120	1,787	154	
72133	COL201908-87	2019	throat swab	384	2,073,782	100	120,747	5,401	26,240	24	7,976	79	4,143	97	
72134	COL201908-88	2019	throat swab	453	2,146,399	100	113,136	4,739	23,828	27	5,917	94	2,790	120	
72135	COL201909-29	2019	throat swab	606	2,164,853	100	104,214	3,573	22,538	32	4,447	115	1,364	153	
72136	COL201909-31	2019	throat swab	517	2,281,466	100	89,281	4,413	21,475	33	4,489	114	1,935	152	
72137	COL201909-32	2019	throat swab	489	2,090,574	100	80,244	4,276	18,656	36	5,421	115	2,350	144	
72138	COL201909-33	2019	throat swab	496	2,087,157	100	85,023	4,208	21,188	29	4,910	106	2,451	132	
72140	COL201808-68	2019	rectal swab	302	2,209,598	253	51,647	7,317	15,717	44	4,338	146	2,496	179	
72141	COL201909-34	2019	throat swab	243	2,075,428	255	86,483	8,541	16,764	36	5,269	118	2,947	144	
72142	COL201909-35	2019	throat swab	224	2,014,319	251	78,046	8,993	17,919	34	5,402	114	3,214	138	
72143	COL201909-36	2019	throat swab	280	2,086,665	253	82,051	7,453	14,653	40	4,548	136	2,473	166	
72144	COL201909-37	2019	throat swab	291	2,199,927	251	80,757	7,560	16,886	39	4,246	144	2,427	178	
72145	COL201909-38	2019	throat swab	278	2,072,264	251	58,792	7,455	14,589	44	4,494	142	2,891	170	
72146	COL201909-43	2019	throat swab	294	2,186,731	253	42,043	7,438	15,003	46	4,576	152	2,689	182	
72147	COL201909-51	2019	throat swab	224	2,041,196	253	81,030	9,113	18,908	34	5,429	108	3,314	131	
72149	COL201909-57	2019	throat swab	264	2,091,580	257	55,283	7,923	16,764	38	5,371	121	3,045	145	
72150	COL201909-58	2019	throat swab	253	2,035,756	252	59,950	8,047	17,752	37	4,909	123	2,903	149	
72151	COL201909-59	2019	throat swab	289	2,225,053	251	51,673	7,700	15,296	45	4,586	154	3,221	183	
72152	COL201909-60	2019	throat swab	261	2,077,030	255	87,828	7,958	16,272	39	4,689	135	2,687	163	
72153	COL201909-62	2019	throat swab	313	2,262,895	253	55,228	7,230	15,888	44	4,245	151	2,496	184	
72154	COL201909-71	2019	throat swab	288	2,140,706	257	67,796	7,434	16,703	40	4,640	133	2,619	163	
72155	COL201909-72	2019	throat swab	309	2,112,738	251	44,491	6,838	13,490	46	4,217	147	2,560	180	
72156	COL201909-73	2019	throat swab	297	2,075,702	253	50,485	6,989	14,040	45	4,294	148	2,479	178	
72157	COL201909-74	2019	throat swab	246	2,012,951	251	68,802	8,183	15,448	38	5,071	128	3,194	153	
72158	COL201909-76	2019	throat swab	280	2,154,136	254	58,915	7,694	17,175	39	4,246	137	2,386	170	
72159	COL201909-77	2019	throat swab	275	2,058,693	251	63,710	7,487	13,432	43	4,241	149	2,752	179	
72160	COL201910-26	2019	throat swab	242	2,054,883	252	81,030	8,492	17,748	37	5,776	118	3,547	140	

72161	COL201910-28	2019	throat swab	266	2,126,641	259	69,596	7,995	17,681	38	5,321	124	2,662	150	
72162	COL201910-29	2019	throat swab	227	2,044,162	254	62,564	9,006	18,553	37	5,608	113	3,881	134	
72163	COL201910-30	2019	throat swab	256	2,024,290	260	78,943	7,908	14,870	41	4,812	134	2,754	162	
72164	COL201910-32	2019	throat swab	292	2,192,873	254	53,624	7,510	15,873	45	4,275	152	2,542	184	
72165	COL201910-33	2019	throat swab	242	2,057,732	251	50,308	8,504	16,570	38	5,345	122	3,552	145	
72166	COL201910-34	2019	throat swab	240	2,061,301	254	62,439	8,589	16,559	40	4,789	130	3,297	156	
72167	COL201910-35	2019	throat swab	129	2,102,824	302	158,804	16,301	49,144	15	10,905	47	5,917	59	
72168	COL201910-36	2019	throat swab	328	4,353,093	252	286,911	13,272	36,481	23	6,207	136	3,860	178	
72169	COL201910-37	2019	throat swab	246	2,046,415	251	57,434	8,319	16,571	37	5,207	122	3,297	147	
72170	COL201910-38	2019	throat swab	235	1,998,816	251	91,059	8,506	16,603	36	5,077	123	3,222	148	
72171	COL201910-39	2019	throat swab	262	1,999,083	256	58,539	7,631	15,381	41	4,634	132	2,544	160	
72172	COL201910-43	2019	throat swab	227	2,015,527	251	65,127	8,879	16,908	35	5,114	122	3,203	147	
72173	COL201910-44	2019	throat swab	244	2,079,465	251	84,272	8,523	17,589	36	4,469	124	3,065	151	
72174	COL201910-45	2019	throat swab	316	2,159,037	253	61,462	6,833	16,501	41	4,548	143	2,664	173	
72175	COL201910-47	2019	throat swab	203	2,008,876	255	49,276	9,896	18,820	37	5,717	109	4,091	129	
72176	COL201910-49	2019	throat swab	227	2,071,839	254	69,537	9,128	17,096	36	5,594	116	3,222	138	
72177	COL201910-50	2019	throat swab	243	2,050,940	251	74,912	8,441	17,175	34	4,547	128	3,126	155	
72178	COL201910-58	2019	throat swab	291	2,156,781	254	78,519	7,412	15,022	44	4,268	149	2,637	181	
72179	COL201910-60	2019	throat swab	276	3,277,247	325	100,681	11,875	26,427	38	5,608	138	3,416	175	Contains additional DNA from Granulicatella sp.
72180	COL201910-64	2019	throat swab	301	2,185,762	253	42,903	7,262	14,584	46	4,560	156	2,625	187	
72181	COL201910-73	2019	throat swab	306	2,193,482	254	53,630	7,169	16,256	45	3,968	155	2,396	190	
72182	COL201910-74	2019	throat swab	234	2,019,949	251	64,238	8,633	15,808	37	5,569	121	3,574	144	
72183	COL201910-76	2019	throat swab	242	2,108,002	251	45,836	8,711	19,056	36	5,310	119	3,387	144	
72184	COL201910-81	2019	throat swab	214	1,993,122	252	88,757	9,314	17,531	32	5,375	109	3,222	133	
72185	COL201910-82	2019	rectal swab	286	2,076,852	253	56,459	7,262	14,503	43	4,366	143	2,544	173	
72186	COL201910-83	2019	throat swab	296	2,076,613	251	56,459	7,016	14,059	45	4,397	145	2,471	175	
72187	COL201910-84	2019	throat swab	281	2,176,465	251	58,093	7,746	16,919	40	4,126	139	2,619	170	
72188	COL201911-28	2019	throat swab	288	2,205,625	252	78,178	7,659	17,944	38	4,262	142	2,490	175	
72189	COL201911-29	2019	throat swab	225	2,049,033	252	67,514	9,107	18,134	38	5,512	121	3,806	143	
72190	COL201911-31	2019	throat swab	277	2,163,324	251	74,974	7,810	17,634	39	4,114	137	2,558	169	
72191	COL201911-32	2019	throat swab	245	2,110,142	255	52,671	8,613	19,143	35	5,255	117	3,262	143	
72193	COL201911-35	2019	throat swab	231	2,020,871	251	64,238	8,749	16,552	36	5,589	120	3,523	142	
72194	COL201911-36	2019	throat swab	226	2,013,410	251	78,073	8,909	17,194	35	5,402	117	3,309	141	
72195	COL201911-38	2019	throat swab	294	2,192,424	261	53,624	7,458	16,493	44	4,090	149	2,286	183	
72196	COL201911-39	2019	throat swab	297	2,061,190	251	53,255	6,941	15,915	42	4,738	142	2,576	169	
72197	COL201911-40	2019	throat swab	294	2,212,782	251	76,658	7,527	16,566	42	3,943	144	2,419	179	
72198	COL201911-42	2019	throat swab	282	2,068,316	251	58,797	7,335	14,502	47	4,436	146	2,758	175	
72199	COL201911-44	2019	throat swab	311	2,225,375	256	82,051	7,156	17,281	39	4,368	140	2,056	177	
72200	COL201911-46	2019	throat swab	244	2,024,432	254	108,272	8,297	16,964	37	4,646	127	3,294	153	
72201	COL201911-48	2019	throat swab	273	2,106,084	251	42,111	7,715	17,146	39	4,489	134	2,682	164	
72202	COL201911-51	2019	throat swab	300	2,060,337	251	52,628	6,868	16,089	42	4,548	141	2,553	169	
72203	COL201911-54	2019	throat swab	84	3,131,309	302	390,226	37,278	121,338	7	21,833	30	9,804	41	
72204	COL201911-57	2019	throat swab	310	2,149,651	251	43,434	6,935	14,470	45	4,195	148	2,312	182	
72205	COL201911-64	2019	throat swab	290	2,187,995	253	42,739	7,545	15,003	46	4,655	153	2,689	184	
72206	COL201911-65	2019	throat swab	243	2,050,771	252	62,772	8,440	16,529	38	5,422	125	3,410	149	
72207	COL201911-66	2019	throat swab	243	2,052,558	254	59,411	8,447	16,586	36	5,287	120	3,298	145	
72208	COL201912-26	2019	throat swab	236	2,039,256	254	81,018	8,641	18,857	36	5,432	112	3,274	136	
72209	COL201912-27	2019	throat swab	310	2,197,788	263	53,451	7,090	14,591	46	3,940	158	2,317	193	
72210	COL201912-28	2019	throat swab	287	2,116,926	251	44,882	7,377	15,824	41	4,325	137	2,615	168	
72211	COL201912-29	2019	throat swab	305	2,238,852	251	74,921	7,341	17,018	41	3,904	150	2,373	186	
72212	COL201912-30	2019	throat swab	256	2,144,483	251	52,686	8,377	19,622	36	4,825	123	3,303	150	
72213	COL201912-34	2019	throat swab	235	1,988,374	251	52,862	8,462	17,086	37	4,533	121	3,126	148	
72214	COL201912-35	2019	throat swab	240	2,054,780	254	76,455	8,562	17,894	34	5,269	118	2,973	143	
72215	COL201912-37	2019	throat swab	217	2,008,931	251	49,320	9,258	17,930	40	5,554	122	3,890	143	
72216	COL201912-40	2019	throat swab	266	2,077,440	252	78,317	7,810	18,196	34	4,656	131	2,689	158	
72217	COL201912-41	2019	throat swab	310	2,219,635	253	77,887	7,161	15,971	43	4,113	149	2,355	184	
72218	COL201903-73	2019	throat swab	221	2,018,430	251	78,103	9,134	19,988	32	5,105	115	3,045	140	
72219	COL201903-42	2019	throat swab	236	2,054,340	251	94,265	8,705	16,570	38	5,672	121	3,759	142	

72220	COL201812-51	2018	throat swab	240	2,045,893	252	80,059	8,525	16,095	36	5,175	122	3,297	147	
72221	COL201812-33	2018	throat swab	279	2,090,691	251	55,723	7,494	15,101	42	4,323	141	2,519	170	
72222	COL201811-33	2018	throat swab	230	2,084,672	253	63,224	9,064	17,060	35	5,508	116	3,974	138	
72223	COL201810-72	2018	throat swab	214	2,015,683	251	78,103	9,420	19,768	32	5,420	111	3,215	135	
72224	COL201810-48	2018	throat swab	224	2,032,838	254	62,306	9,076	17,467	35	5,771	117	3,799	138	
72225	COL201810-37	2018	throat swab	223	2,019,411	251	71,661	9,056	17,042	39	5,611	120	3,776	141	
72226	COL201809-71	2018	throat swab	242	2,098,857	251	54,472	8,673	16,627	37	5,366	123	3,466	148	
72227	COL201809-37	2018	throat swab	241	2,072,880	255	63,731	8,602	16,064	39	5,569	124	3,090	148	
72228	COL201809-28	2018	throat swab	310	2,256,579	251	51,647	7,280	16,598	44	4,626	146	2,701	177	
72229	COL201808-28	2018	throat swab	310	2,256,579	251	51,647	7,280	16,598	44	4,626	146	2,701	177	
72230	COL201807-50	2018	throat swab	254	2,111,851	258	59,057	8,315	14,415	40	4,880	137	3,477	162	
72231	COL201805-59	2018	throat swab	265	2,057,796	265	58,075	7,766	15,726	40	4,670	134	2,617	162	
72232	COL201804-44	2018	throat swab	263	2,048,461	252	41,486	7,789	15,094	43	4,286	141	2,627	171	
72697	COL201803-39	2018	throat swab	270	2,095,095	253	55,283	7,760	19,209	35	5,118	122	2,986	147	
72698	COL201806-50	2018	urethral swab	221	2,036,435	252	62,173	9,215	18,513	35	6,416	106	3,574	125	
72699	COL201904-27	2019	throat swab	245	2,015,214	251	78,111	8,226	16,576	38	4,582	130	2,804	158	
72700	COL201904-42	2019	throat swab	253	2,074,041	254	63,731	8,198	16,926	37	5,289	123	2,643	149	
72702	COL201912-39	2019	rectal swab	264	2,059,070	252	78,317	7,800	18,571	37	4,813	131	3,051	156	
72703	COL201912-42	2019	throat swab	253	2,060,460	253	85,184	8,145	14,820	43	5,288	130	3,195	155	
72704	COL201912-45	2019	throat swab	257	2,087,505	258	58,783	8,123	15,839	38	5,287	124	3,045	150	
72705	COL201912-47	2019	throat swab	265	2,187,948	253	80,035	8,257	16,095	41	5,441	136	3,290	161	
72706	COL201912-48	2019	throat swab	233	2,034,187	251	53,463	8,731	18,471	32	5,291	115	3,299	139	
72707	COL201912-52	2019	throat swab	290	2,058,496	251	94,822	7,099	16,971	39	4,698	129	2,448	158	
72708	COL201912-53	2019	throat swab	245	2,067,026	251	78,262	8,437	15,999	37	5,170	128	3,547	151	
72709	COL201912-55	2019	throat swab	312	2,257,219	254	51,647	7,235	15,567	45	4,213	153	2,707	186	
72710	COL201912-57	2019	throat swab	263	2,153,775	261	46,416	8,190	18,312	39	4,928	130	3,177	158	
72711	COL201912-59	2019	throat swab	286	2,076,611	253	56,459	7,261	14,502	43	4,311	141	2,531	172	
72712	COL201912-66	2019	throat swab	247	1,991,204	259	52,865	8,062	17,121	39	4,468	130	2,770	158	
72713	COL201912-71	2019	throat swab	254	2,027,618	254	58,193	7,983	16,285	41	4,559	134	2,789	161	
72714	COL201912-72	2019	throat swab	264	2,231,028	251	85,205	8,451	19,458	34	5,453	117	3,299	144	
72715	COL201912-75	2019	throat swab	224	2,016,981	251	60,950	9,005	19,622	33	5,289	114	3,215	138	
72716	COL201912-81	2019	throat swab	235	2,061,817	251	75,937	8,774	17,045	35	5,185	122	3,708	146	
72717	COL201912-82	2019	throat swab	259	2,077,498	252	99,269	8,022	16,018	37	4,815	126	2,775	154	
72718	COL201912-83	2019	throat swab	252	2,061,752	252	78,312	8,182	16,647	36	5,049	127	3,303	152	
72719	COL201912-85	2019	throat swab	268	2,119,947	254	106,383	7,911	18,760	36	4,681	122	2,666	151	
92904	COL201801-37	2018	throat swab	3,925	3,959,016	128	24,155	1,009	1,675	579	466	2,564	353	3,025	Contains additional DNA from Neisseria sp.
92905	COL201801-43	2018	throat swab	498	2,334,343	128	64,733	4,688	20,811	33	2,916	130	510	228	
92906	COL201801-62	2018	throat swab	616	2,372,480	128	66,867	3,852	21,552	36	2,067	133	472	309	
92908	COL201803-26	2018	throat swab	457	2,225,484	128	70,214	4,870	18,893	35	3,451	134	760	198	
92909	COL201803-27	2018	throat swab	475	2,264,492	128	87,198	4,768	20,284	33	2,769	125	533	206	
92910	COL201803-29	2018	throat swab	557	2,248,232	128	84,561	4,037	19,495	36	3,013	125	471	279	
92911	COL201803-30	2018	throat swab	495	2,220,322	128	61,323	4,486	20,632	33	3,430	114	487	234	
92912	COL201803-31	2018	throat swab	682	2,390,091	128	58,661	3,505	15,968	42	1,063	190	476	400	
92913	COL201803-35	2018	throat swab	454	2,296,123	128	85,542	5,058	24,364	31	4,085	108	511	183	
92914	COL201803-38	2018	throat swab	542	2,278,248	128	54,812	4,204	18,656	37	2,682	145	487	274	
92916	COL201803-40	2018	throat swab	434	2,223,604	128	101,312	5,124	32,473	23	4,396	89	499	170	
92917	COL201803-41	2018	throat swab	596	2,318,082	128	74,034	3,890	18,402	38	1,676	154	485	313	
92918	COL201803-42	2018	throat swab	618	2,309,000	128	54,901	3,737	15,054	44	1,655	184	478	349	
92919	COL201803-45	2018	throat swab	770	2,484,691	128	74,588	3,227	16,588	45	968	226	465	460	
92920	COL201803-46	2018	throat swab	333	2,144,356	128	99,870	6,440	22,847	30	6,493	90	1,604	116	
92921	COL201803-47	2018	throat swab	696	2,444,422	128	67,762	3,513	17,303	44	1,222	191	472	399	
92922	COL201803-48	2018	throat swab	581	2,228,213	128	58,815	3,836	16,362	44	2,203	169	491	307	
92923	COL201803-57	2018	throat swab	513	2,357,287	128	76,859	4,596	19,864	36	2,357	142	526	234	
92924	COL201803-59	2018	throat swab	373	2,161,015	128	92,737	5,794	20,383	32	4,737	108	1,449	144	
92925	COL201803-60	2018	throat swab	463	2,246,905	128	78,688	4,853	23,382	32	4,246	105	496	197	
92926	COL201803-61	2018	throat swab	396	2,216,267	128	117,947	5,597	22,253	28	4,825	101	850	149	
92927	COL201803-62	2018	throat swab	440	2,192,404	128	109,844	4,983	17,621	34	3,743	124	548	196	
92928	COL201803-63	2018	throat swab	492	2,288,410	128	85,642	4,652	30,288	24	3,810	91	482	222	

92929	COL201803-66	2018	throat swab	472	2,198,680	128	117,556	4,659	20,667	31	3,231	118	498	214	
92930	COL201803-67	2018	urethral swab	359	2,145,927	128	119,679	5,978	20,435	31	5,393	104	1,677	136	
92931	COL201803-68	2018	throat swab	376	2,170,603	128	87,082	5,773	22,075	28	4,727	96	1,152	139	
92933	COL201803-75	2018	throat swab	506	2,243,413	128	67,465	4,434	18,301	39	2,929	141	513	230	
92934	COL201803-82	2018	throat swab	533	2,290,206	128	70,687	4,297	20,891	36	2,478	141	495	263	
92935	COL201803-83	2018	throat swab	393	2,301,452	128	88,583	5,857	25,570	28	4,657	104	1,104	149	
92936	COL201804-26	2018	throat swab	436	2,371,750	128	100,423	5,440	23,674	27	4,120	106	964	157	
92937	COL201804-28	2018	throat swab	451	2,139,076	128	63,679	4,743	16,622	42	2,806	153	946	214	
92938	COL201804-29	2018	throat swab	437	2,207,956	128	85,454	5,053	24,891	28	4,491	98	500	185	
92940	COL201804-32	2018	throat swab	433	2,249,793	128	86,109	5,196	25,831	28	4,782	95	520	167	
92941	COL201804-34	2018	throat swab	454	2,265,219	128	90,531	4,990	24,708	27	3,742	108	514	184	
92942	COL201804-35	2018	throat swab	428	2,203,654	128	63,205	5,149	18,036	40	3,781	131	929	186	
92943	COL201804-36	2018	throat swab	321	2,140,052	128	75,888	6,667	29,556	25	4,939	84	1,844	115	
92944	COL201804-37	2018	throat swab	553	2,237,744	128	60,740	4,047	17,816	40	2,261	133	474	281	
92945	COL201804-38	2018	throat swab	595	2,243,983	128	57,105	3,772	15,434	43	1,960	157	482	329	
92947	COL201804-46	2018	throat swab	393	2,167,780	128	75,801	5,516	21,509	33	5,380	105	734	151	
92948	COL201804-47	2018	throat swab	532	2,291,473	128	82,767	4,308	22,374	29	2,522	118	492	247	
92949	COL201804-49	2018	throat swab	502	2,207,402	128	62,130	4,398	16,101	46	2,976	160	502	247	
92950	COL201804-51	2018	throat swab	512	2,225,386	128	93,212	4,347	20,679	37	3,042	118	481	231	
92951	COL201804-58	2018	throat swab	555	2,265,552	128	79,082	4,083	21,837	32	2,133	126	482	284	
92952	COL201804-66	2018	throat swab	465	2,199,104	128	75,647	4,730	19,783	32	3,057	131	536	215	
92954	COL201804-68	2018	throat swab	524	2,227,013	128	58,272	4,251	17,521	38	2,706	152	501	265	
92955	COL201804-69	2018	throat swab	479	2,148,163	128	63,043	4,485	18,385	38	3,037	136	498	226	
92956	COL201804-73	2018	throat swab	413	2,188,298	128	93,354	5,299	23,858	31	4,606	97	651	150	
92957	COL201804-74	2018	throat swab	456	2,175,431	128	99,774	4,771	18,186	39	3,283	130	574	206	
92958	COL201804-75	2018	throat swab	633	2,491,225	202	69,916	3,936	18,104	42	1,811	179	474	348	
92959	COL201804-81	2018	throat swab	532	2,375,363	128	70,085	4,465	19,196	44	2,640	155	535	245	
92960	COL201805-05	2018	urethral swab	541	2,279,227	128	54,427	4,213	15,551	47	2,332	170	503	277	
92961	COL201805-29	2018	throat swab	542	2,317,302	128	58,476	4,276	20,220	36	2,810	144	495	267	
92962	COL201805-31	2018	throat swab	505	2,198,546	128	80,753	4,354	19,923	32	3,073	121	492	245	
92963	COL201805-32	2018	throat swab	353	2,139,000	128	68,038	6,060	23,491	30	6,314	93	1,321	123	
92964	COL201805-33	2018	throat swab	502	2,218,715	128	83,823	4,420	21,899	32	3,502	105	485	227	
92965	COL201805-34	2018	throat swab	583	2,262,475	128	79,405	3,881	20,695	36	2,094	143	478	311	
92966	COL201805-36	2018	throat swab	596	2,318,555	128	83,614	3,891	19,114	35	1,812	156	482	325	
92967	COL201805-37	2018	throat swab	487	2,279,723	128	99,404	4,682	21,898	31	2,995	121	501	220	
92968	COL201805-38	2018	throat swab	472	2,316,014	128	79,754	4,907	21,942	34	3,716	121	543	193	
92969	COL201805-39	2018	throat swab	505	2,415,268	128	88,316	4,783	17,801	42	2,895	153	797	231	
92970	COL201805-40	2018	throat swab	444	2,184,574	128	73,431	4,921	24,229	30	3,719	107	501	187	
92971	COL201805-42	2018	throat swab	523	2,249,670	128	89,749	4,302	24,308	28	3,013	105	481	244	
92972	COL201805-43	2018	throat swab	484	2,206,805	128	61,815	4,560	20,220	33	4,013	118	492	220	
92974	COl201805-45	2018	throat swab	564	2,252,917	128	68,209	3,995	14,456	48	2,284	171	493	294	
92975	COL201805-46	2018	throat swab	591	2,313,817	128	89,161	3,916	14,875	49	2,386	178	494	311	
92977	COL201805-49	2018	throat swab	400	2,145,590	128	48,402	5,364	18,177	41	4,336	128	931	174	
92978	COL201805-50	2018	throat swab	531	2,232,419	128	84,257	4,205	18,847	33	2,244	137	485	266	
92979	COL201805-54	2018	throat swab	557	2,293,933	128	94,518	4,119	28,943	25	1,795	108	484	290	
92980	COL201805-55	2018	throat swab	532	2,258,719	128	64,366	4,246	19,479	38	2,973	139	493	261	
92981	COL201805-56	2018	throat swab	474	2,283,196	128	64,310	4,817	19,898	37	3,083	130	782	198	
92983	COL201805-64	2018	throat swab	507	2,241,765	128	92,624	4,422	30,775	24	3,089	96	471	244	
92984	COL201805-68	2018	throat swab	550	2,369,172	128	92,032	4,308	20,189	37	2,828	144	490	265	
92985	COL201805-69	2018	throat swab	457	2,230,206	128	76,601	4,881	24,549	31	4,087	98	496	195	
92986	COL201806-27	2018	throat swab	449	2,314,970	128	60,003	5,156	21,762	36	3,483	127	743	192	
92988	COL201806-30	2018	throat swab	473	2,263,798	128	65,221	4,787	22,259	35	3,528	122	500	206	
92989	COL201806-32	2018	throat swab	365	2,231,851	128	118,024	6,115	26,297	25	6,060	96	1,422	131	
92990	COL201806-35	2018	throat swab	410	2,286,954	128	79,897	5,578	32,454	22	4,359	88	635	152	
92991	COL201806-36	2018	throat swab	519	2,205,623	128	66,760	4,250	17,939	36	2,450	147	491	266	
92993	COL201806-38	2018	throat swab	387	2,185,221	128	65,258	5,647	20,836	33	4,386	116	1,243	159	
92994	COL201806-39	2018	throat swab	718	2,360,188	128	83,485	3,288	19,806	35	685	193	466	426	
92996	COL201806-45	2018	throat swab	495	2,275,002	128	74,024	4,596	23,842	30	3,368	113	492	219	

92997	COL201806-48	2018	throat swab	353	2,145,440	128	99,017	6,078	36,686	21	5,491	77	785	119	
92998	COL201806-66	2018	throat swab	465	2,385,480	128	99,880	5,131	21,894	34	3,595	122	651	190	
92999	COL201806-67	2018	throat swab	433	2,239,965	128	73,325	5,174	20,635	31	3,933	112	727	176	
93000	COL201806-68	2018	throat swab	491	2,299,023	128	64,822	4,683	17,733	37	2,858	140	600	226	
93001	COL201806-69	2018	throat swab	463	2,348,175	128	125,791	5,072	25,049	30	4,361	106	526	191	
93002	COL201806-70	2018	throat swab	318	2,155,055	128	93,189	6,777	24,939	26	5,434	87	1,836	116	
93003	COL201806-71	2018	throat swab	529	2,243,409	128	85,570	4,241	19,940	32	2,619	128	489	275	
93004	COL201806-72	2018	throat swab	610	2,376,010	128	67,638	3,896	16,861	43	1,999	173	489	319	
93005	COL201806-77	2018	throat swab	534	2,258,203	128	60,779	4,229	17,842	40	2,559	143	490	277	
93006	COL201806-80	2018	throat swab	681	2,440,842	128	42,189	3,585	14,723	53	1,729	206	482	384	
93007	COL201806-81	2018	throat swab	946	2,312,231	128	56,785	2,445	13,920	47	1,083	214	447	430	
93008	COL201806-86	2018	throat swab	530	2,183,941	128	49,015	4,121	12,423	51	2,401	185	608	270	
93009	COL201806-87	2018	throat swab	446	2,212,546	128	88,327	4,961	24,004	30	4,004	105	497	188	
93010	COL201807-30	2018	throat swab	491	2,359,737	128	66,771	4,806	17,965	41	2,957	150	707	216	
93011	COL201807-31	2018	throat swab	508	2,296,795	128	64,120	4,522	20,016	34	2,824	132	500	242	
93012	COL201807-32	2018	throat swab	477	2,169,695	128	59,301	4,549	17,875	37	3,278	140	501	225	
93013	COL201807-34	2018	throat swab	441	2,266,952	128	98,396	5,141	20,549	35	3,597	121	791	187	
93014	COL201807-36	2018	throat swab	2,305	3,548,458	128	78,605	1,540	9,508	75	477	1,509	453	1,891	Contains additional DNA from Ligilactobacillus sp.
93015	COL201807-38	2018	throat swab	1,068	2,630,977	128	87,394	2,464	19,395	39	510	481	465	752	
93016	COL201807-39	2018	throat swab	2,717	3,839,606	128	59,590	1,414	6,277	113	472	1,852	450	2,268	Contains additional DNA from Ligilactobacillus sp.
93017	COL201807-40	2018	throat swab	591	2,324,937	128	58,774	3,934	23,634	32	1,829	129	473	306	
93018	COL201807-42	2018	throat swab	454	2,183,191	128	69,374	4,809	24,055	29	3,111	113	495	205	
93020	COL201807-44	2018	throat swab	476	2,307,400	128	88,410	4,848	26,423	29	2,894	112	501	208	
93021	COL201807-47	2018	urethral swab	716	2,283,989	128	62,117	3,190	12,167	57	1,174	237	482	430	
93023	COL201807-51	2018	throat swab	3,194	4,359,353	128	63,381	1,365	3,357	195	472	2,211	451	2,683	Contains additional DNA from Ligilactobacillus sp.
93026	COL201807-64	2018	throat swab	440	2,305,125	128	100,444	5,239	28,620	25	4,208	93	619	156	
93027	COL201807-65	2018	throat swab	477	2,232,893	128	112,729	4,682	28,351	24	4,101	90	475	200	
93028	COL201807-66	2018	throat swab	532	2,259,946	128	96,229	4,249	33,625	23	3,866	84	471	248	
93029	COL201807-70	2018	throat swab	344	2,181,321	128	114,206	6,342	37,178	18	7,233	59	1,168	91	
93031	COL201808-27	2018	throat swab	622	2,295,251	128	115,683	3,691	29,081	26	1,157	120	462	341	
93033	COL201808-29	2018	throat swab	627	2,328,601	128	63,319	3,714	15,159	47	1,693	174	478	342	
93034	COL201808-33	2018	throat swab	471	2,352,239	128	117,020	4,995	25,888	26	4,302	95	494	179	
93036	COL201808-37	2018	throat swab	477	2,305,491	128	96,386	4,834	25,678	28	3,556	113	492	211	
93037	COL201808-38	2018	throat swab	542	2,300,221	128	64,500	4,244	26,636	29	2,850	106	473	271	
93038	COL201808-40	2018	throat swab	2428	4,684,243	128	53,298	1,930	5,412	155	519	1,401	468	1,880	Contains additional DNA from Ligilactobacillus sp.
93039	COL201808-41	2018	throat swab	494	2,223,265	128	93,143	4,501	22,297	33	3,753	111	489	228	
93040	COL201808-42	2018	rectal swab	421	2,209,885	128	118,213	5,250	26,536	25	4,646	88	501	161	
93041	COL201808-46	2018	throat swab	955	4,125,756	128	65,156	4,321	14,817	76	2,293	325	547	494	Contains additional DNA from Granulicatella sp.
93042	COL201808-47	2018	throat swab	629	2,269,374	128	113,549	3,608	20,636	31	1,445	136	469	341	
93043	COL201808-48	2018	throat swab	651	2,546,443	128	70,565	3,912	22,061	34	1,984	153	474	343	
93044	COL201808-49	2018	throat swab	472	2,195,193	128	92,662	4,651	24,343	30	3,987	108	489	197	
93045	COL201808-50	2018	throat swab	555	2,255,645	128	75,138	4,065	22,978	32	2,440	120	472	277	
93046	COL201808-51	2018	throat swab	562	2,249,523	128	61,682	4,003	23,151	30	2,563	109	471	271	
93047	COL201808-54	2018	throat swab	468	2,233,320	128	75,976	4,773	20,624	33	3,197	120	498	209	
93048	COL201808-62	2018	throat swab	480	2,252,829	128	74,239	4,694	22,262	31	3,149	114	491	214	
93050	COL201808-72	2018	throat swab	587	2,466,660	128	67,501	4,203	16,077	49	2,452	183	589	284	
93051	COL201809-27	2018	throat swab	615	2,432,019	128	68,961	3,955	18,442	39	2,238	162	475	326	
93053	COL201809-33	2018	throat swab	726	2,369,061	128	84,531	3,264	18,343	37	946	195	470	419	
93055	COL201809-38	2018	throat swab	737	2,478,841	128	59,442	3,364	17,505	47	789	197	472	439	
93056	COL201809-40	2018	throat swab	5063	4,594,766	128	16,547	908	1,415	741	450	3,375	315	3,943	Contains additional DNA from Neisseria meningitidis
93057	COL201809-43	2018	throat swab	646	2,467,260	128	57,179	3,820	18,473	43	1,616	178	485	352	
93058	COL201809-45	2018	throat swab	564	2,297,174	128	51,785	4,074	18,422	41	2,276	151	487	296	
93059	COL201809-46	2018	throat swab	513	2,318,315	128	100,120	4,520	22,506	30	2,832	122	488	248	
93060	COL201809-53	2018	throat swab	451	2,217,445	128	78,982	4,917	24,996	29	3,682	114	503	198	
93061	COL201809-56	2018	throat swab	609	2,304,474	128	68,666	3,785	21,156	35	1,649	145	475	334	
93062	COL201809-57	2018	throat swab	423	2,218,664	128	78,421	5,246	24,877	29	4,003	106	735	167	
93063	COL201809-58	2018	throat swab	515	2,225,283	128	103,472	4,321	23,644	29	3,135	111	484	249	
93065	COL201809-63	2018	throat swab	503	2,243,389	128	100,939	4,461	20,354	31	2,794	125	493	243	

93067	COL201809-72	2018	throat swab	655	2,347,736	128	68,550	3,585	21,123	33	1,375	155	468	358	
93068	COL201810-26	2018	throat swab	510	2,328,826	128	74,108	4,567	20,457	34	2,612	126	496	237	
93069	COL201810-28	2018	throat swab	796	2,406,724	128	58,830	3,024	18,579	39	522	259	466	506	
93070	COL201810-30	2018	throat swab	573	2,289,017	128	91,612	3,995	25,525	29	1,660	121	478	304	
93072	COL201810-33	2018	throat swab	465	2,246,841	128	91,295	4,832	24,752	27	3,165	103	499	199	
93073	COL201810-34	2018	throat swab	2136	3,216,334	128	53,188	1,506	10,761	68	470	1,410	450	1,759	Contains additional DNA from Ligilactobacillus sp.
93074	COL201810-35	2018	throat swab	390	2,183,185	128	103,089	5,598	27,306	25	6,986	82	501	142	
93075	COL201810-36	2018	throat swab	618	2,391,058	128	84,660	3,870	24,962	32	1,762	139	472	331	
93079	COL201810-45	2018	throat swab	631	2,291,368	128	86,433	3,632	22,130	31	1,286	149	467	356	
93081	COL201810-51	2018	throat swab	521	2,306,663	128	91,324	4,428	26,313	28	2,802	112	485	242	
93082	COL201810-53	2018	throat swab	425	2,176,728	128	103,468	5,122	21,657	30	3,829	109	628	169	
93083	COL201810-55	2018	throat swab	2502	3,296,727	128	116,763	1,318	17,767	41	451	1,732	439	2,102	
93084	COL201810-66	2018	throat swab	441	2,270,249	128	159,175	5,148	29,025	24	4,720	94	497	166	
93086	COL201810-74	2018	throat swab	1328	2,381,623	128	62,298	1,794	13,097	53	493	317	254	609	
93087	COL201810-79	2018	throat swab	552	2,336,131	128	85,673	4,233	25,308	29	2,703	112	472	278	
93088	COL201810-80	2018	throat swab	427	2,256,766	128	80,785	5,286	28,773	26	4,177	94	503	167	
93089	COL201810-81	2018	throat swab	506	2,242,941	128	111,818	4,433	32,252	21	3,432	82	470	244	
93090	COL201811-12	2018	urethral swab	518	2,241,173	128	62,870	4,327	17,483	35	2,879	136	484	242	
93091	COL201811-26	2018	throat swab	654	2,326,807	128	100,966	3,558	26,264	24	655	132	461	372	
93092	COL201811-27	2018	throat swab	769	2,376,983	128	88,063	3,092	29,620	25	502	233	459	483	
93093	COL201811-28	2018	throat swab	884	2,420,822	128	95,950	2,739	22,288	34	485	329	454	587	
93094	COL201811-29	2018	throat swab	704	2,381,842	128	84,412	3,384	25,815	29	795	160	460	399	
93095	COL201811-30	2018	throat swab	559	2,256,292	128	106,866	4,037	24,680	28	1,831	113	468	296	
93096	COL201811-32	2018	throat swab	479	2,278,871	128	154,474	4,758	26,335	24	5,502	83	478	215	
93098	COL201811-35	2018	throat swab	332	2,170,364	128	93,403	6,538	37,132	19	7,194	65	1,144	97	
93099	COL201811-37	2018	throat swab	495	2,213,852	128	86,588	4,473	23,277	31	2,820	113	487	226	
93100	COL201811-38	2018	throat swab	531	2,275,974	128	90,107	4,287	26,626	28	2,164	110	474	266	
93101	COL201811-39	2018	throat swab	465	2,352,764	128	97,253	5,060	25,312	31	3,385	119	642	187	
93102	COL201811-40	2018	throat swab	369	2,178,111	128	114,702	5,903	34,098	22	6,256	75	888	117	
93103	COL201811-41	2018	throat swab	516	2,417,616	128	94,376	4,686	28,489	26	3,111	110	490	225	
93104	COL201811-43	2018	throat swab	510	2,301,637	128	103,650	4,514	29,655	24	2,925	100	483	223	
93105	COL201811-44	2018	throat swab	476	2,298,725	128	67,021	4,830	20,563	33	3,583	117	638	185	
93106	COL201811-46	2018	throat swab	888	2,519,509	128	62,164	2,838	22,962	36	489	302	458	569	
93108	COL201811-49	2018	throat swab	648	2,441,592	128	52,049	3,768	18,397	42	1,992	171	472	335	
93109	COL201811-50	2018	throat swab	1039	4,100,496	128	35,484	3,947	8,095	153	2,063	514	1,038	646	Contains additional DNA from Moraxella catarrhalis
93111	COL201811-52	2018	throat swab	757	2,399,843	128	64,055	3,171	18,433	38	2,954	145	487	241	
93112	COL201811-56	2018	throat swab	319	2,155,240	128	124,744	6,757	26,049	26	8,412	82	2,510	104	
93116	COL201811-62	2018	throat swab	1022	2,378,058	128	79,843	2,327	16,560	43	1,497	193	433	389	
93117	COL201812-28	2018	throat swab	383	2,161,330	128	84,885	5,644	20,523	32	4,732	111	1,252	150	
93118	COL201812-29	2018	throat swab	883	2,402,373	128	57,691	2,721	14,659	50	2,241	185	473	319	
93121	COL201812-34	2018	throat swab	403	2,263,359	128	72,722	5,617	28,963	26	4,865	88	895	144	
93122	COL201812-35	2018	throat swab	734	2,362,979	128	43,094	3,220	14,565	52	1,153	222	469	432	
93123	COL201812-36	2018	throat swab	394	2,241,491	128	84,192	5,690	24,517	26	4,297	96	1,281	139	
93124	COL201812-39	2018	throat swab	472	2,287,631	128	99,849	4,847	23,851	29	3,086	113	503	200	
93125	COL201812-40	2018	throat swab	417	2,435,118	128	72,192	5,840	26,811	29	4,736	104	1,136	156	
93126	COL201812-41	2018	throat swab	545	2,420,997	128	93,242	4,443	25,589	29	3,306	112	477	240	
93127	COL201812-42	2018	throat swab	357	2,178,103	128	123,327	6,102	30,680	22	6,308	78	1,080	114	
93128	COL201812-43	2018	throat swab	387	2,203,737	128	106,344	5,695	38,245	20	6,335	67	501	126	
93129	COL201812-45	2018	throat swab	398	2,187,967	128	96,504	5,498	27,355	26	4,146	100	882	151	
93130	COL201812-46	2018	throat swab	440	2,198,990	128	70,456	4,998	22,044	33	3,753	106	620	169	
93131	COL201812-47	2018	throat swab	533	2,274,225	128	94,022	4,267	19,955	36	2,098	141	488	260	
93132	COL201812-50	2018	throat swab	639	2,261,799	128	78,343	3,540	17,939	40	1,271	164	467	369	
93134	COL201812-57	2018	throat swab	632	2,351,507	128	140,521	3,721	29,177	23	1,041	121	464	348	
93135	COL201812-59	2018	throat swab	275	2,139,048	128	103,493	7,779	40,041	19	8,807	60	2,886	79	
93136	COL201812-61	2019	throat swab	425	2,261,879	128	82,870	5,323	28,681	25	4,744	92	598	151	
93137	COL201901-26	2019	throat swab	513	2,313,256	128	65,105	4,510	23,529	31	2,999	123	494	246	
93138	COL201901-27	2019	throat swab	4002	4,439,605	128	57,446	1,110	1,853	210	457	2,979	443	3,472	Contains additional DNA from Capnocytophaga sp.
93139	COL201901-28	2019	throat swab	336	2,177,633	128	76,159	6,482	22,121	28	6,137	94	1,782	128	

93140	COL201901-30	2019	throat swab	695	2,399,430	128	90,215	3,453	20,270	30	1,101	169	463	390	
93141	COL201901-31	2019	throat swab	418	2,257,433	128	72,006	5,401	26,167	27	4,614	99	625	155	
93142	COL201901-32	2019	throat swab	444	2,320,558	128	97,890	5,227	27,290	26	4,046	98	502	175	
93143	COL201901-33	2019	throat swab	358	2,173,279	128	95,404	6,071	35,001	21	5,253	79	1,091	120	
93144	COL201901-34	2019	throat swab	329	2,172,841	128	117,834	6,605	37,910	19	7,646	62	1,338	93	
93145	COL201901-35	2019	throat swab	325	2,196,257	128	96,911	6,758	30,964	22	6,727	76	1,988	103	
93146	COL201901-36	2019	throat swab	370	2,172,700	128	93,464	5,873	22,774	33	6,050	97	1,274	135	
93147	COL201901-38	2019	throat swab	793	2,404,862	128	85,773	3,033	24,291	33	528	267	467	512	
93148	COL201901-39	2019	throat swab	386	2,201,160	128	126,981	5,703	25,624	26	5,860	84	665	137	
93149	COL201901-40	2019	throat swab	701	2,401,847	128	75,287	3,427	19,840	36	1,103	182	463	405	
93150	COL201901-45	2019	throat swab	440	2,253,111	128	100,465	5,121	25,099	27	4,204	97	500	172	
93152	COL201901-50	2019	throat swab	524	2,332,567	128	93,179	4,452	35,457	19	2,692	80	470	250	
93153	COL201901-52	2019	throat swab	298	2,150,444	128	151,788	7,217	29,915	21	9,495	67	2,704	86	
93154	COL201901-53	2019	throat swab	471	2,290,300	128	100,038	4,863	25,034	27	3,705	106	500	199	
93155	COL201901-57	2019	throat swab	414	2,275,149	128	99,981	5,496	25,193	25	4,547	98	762	154	
93156	COL201901-59	2019	throat swab	458	2,286,610	128	82,682	4,993	29,365	26	4,926	92	495	178	
93157	COL201901-61	2019	throat swab	550	2,269,160	128	87,658	4,126	21,012	31	2,542	127	480	272	
93158	COL201901-63	2019	throat swab	595	2,261,959	128	72,061	3,802	21,014	35	1,908	145	471	318	
93159	COL201902-30	2019	throat swab	475	2,318,079	128	88,404	4,881	24,811	30	2,959	115	627	198	
93160	COL201902-33	2019	throat swab	493	2,436,094	128	104,944	4,942	21,627	32	3,656	124	518	211	
93161	COL201902-37	2019	throat swab	447	2,336,326	128	100,846	5,227	22,781	28	4,056	105	632	170	
93162	COL201902-42	2019	throat swab	453	2,222,137	128	84,446	4,906	21,779	30	3,920	110	535	189	
93163	COL201902-46	2019	throat swab	650	2,305,735	128	75,517	3,548	23,306	31	1,030	148	464	370	
93167	COL201902-52	2019	throat swab	355	2,205,678	128	83,946	6,214	27,666	26	5,268	91	1,420	129	
93168	COL201902-54	2019	throat swab	445	2,255,109	128	172,409	5,068	28,063	23	4,361	99	705	157	
93169	COL201902-56	2019	throat swab	301	2,151,500	128	139,767	7,148	37,455	20	8,819	61	2,015	83	
93170	COL201903-26	2019	throat swab	636	2,286,210	128	75,931	3,595	16,841	38	1,483	165	468	356	
93171	COL201903-27	2019	throat swab	387	2,190,192	128	93,338	5,660	30,290	25	5,810	80	823	125	
93172	COL201903-28	2019	throat swab	479	2,455,504	128	91,442	5,127	20,202	34	3,228	142	904	211	
93173	COL201903-30	2019	throat swab	377	2,208,977	128	75,298	5,860	24,835	29	5,213	100	1,214	140	
93174	COL201903-31	2019	throat swab	393	2,191,778	128	71,618	5,578	24,903	27	6,440	81	514	136	
93175	COL201903-32	2019	throat swab	496	2,240,327	128	92,017	4,517	24,998	24	2,986	103	483	227	
93177	COL201903-34	2019	urethral swab	357	2,224,010	128	109,066	6,230	30,772	22	5,716	77	1,017	119	
93178	COL201903-35	2019	throat swab	467	2,264,181	128	85,438	4,849	20,057	33	3,306	124	746	193	
93179	COL201903-37	2019	throat swab	411	2,183,852	128	109,622	5,314	28,379	24	4,100	94	709	154	
93180	COL201903-38	2019	throat swab	424	2,222,517	128	64,484	5,242	23,809	30	4,377	106	820	162	
93181	COL201903-41	2019	throat swab	365	2,184,430	128	135,093	5,985	23,651	25	8,155	82	631	122	
93183	COL201903-43	2019	throat swab	620	2,332,352	128	92,371	3,762	22,632	28	1,654	128	464	335	
93184	COL201903-44	2019	throat swab	368	2,195,695	128	98,440	5,967	36,013	22	6,634	67	672	112	
93185	COL201903-47	2019	throat swab	460	2,238,393	128	102,999	4,867	26,815	28	3,140	110	577	186	
93186	COL201903-48	2019	throat swab	1,098	2,518,911	128	77,952	2,295	14,581	51	1,210	224	431	441	
93187	COL201903-52	2019	throat swab	519	2,417,399	128	87,016	4,658	27,556	28	3,457	100	484	215	
93188	COL201903-54	2019	throat swab	722	2,526,059	128	122,550	3,499	27,336	29	870	164	460	414	
93189	COL201903-55	2019	throat swab	431	2,323,443	128	100,218	5,391	35,339	21	5,204	81	500	166	
93190	COL201903-59	2019	throat swab	632	2,303,607	128	101,807	3,645	28,856	26	1,080	118	462	348	
93191	COL201903-64	2019	throat swab	510	2,355,641	128	144,818	4,619	35,939	17	3,049	78	473	229	
93192	COL201903-66	2019	throat swab	478	2,287,912	128	121,598	4,787	32,125	22	4,327	89	489	206	
93193	COL201903-71	2019	throat swab	308	2,255,963	128	94,402	7,325	31,628	25	7,154	80	2,323	107	
93195	COL201904-32	2019	throat swab	459	2,300,283	128	75,588	5,012	25,111	28	3,245	113	734	184	
93196	COL201904-37	2019	throat swab	457	2,307,736	128	81,658	5,050	24,086	31	3,856	107	608	171	
93197	COL201904-38	2019	throat swab	1,074	2,280,281	123	86,491	2,124	20,196	33	1,045	170	272	404	
93198	COL201904-39	2019	throat swab	464	2,373,019	128	98,828	5,115	25,794	29	3,735	107	631	179	
93199	COL201904-40	2019	throat swab	277	2,131,371	128	104,582	7,695	24,320	27	8,103	83	2,799	103	
93200	COL201904-43	2019	throat swab	365	2,221,240	128	160,586	6,086	36,552	18	6,465	67	952	104	
93201	COL201904-44	2019	throat swab	326	2,164,064	128	101,455	6,639	27,279	25	6,636	73	2,046	99	
93202	COL201904-45	2019	throat swab	400	2,326,346	128	100,951	5,816	28,840	24	4,995	89	1,106	132	
93203	COL201904-46	2019	throat swab	349	2,256,142	128	93,644	6,465	30,098	26	4,986	85	1,330	123	
93204	COL201904-49	2019	throat swab	455	2,441,208	128	118,461	5,366	21,314	34	4,183	126	947	186	

93205	COL201904-52	2019	throat swab	422	2,453,692	128	98,528	5,815	26,908	26	4,256	107	1,119	159	
93206	COL201904-56	2019	throat swab	387	2,247,632	128	100,222	5,808	27,704	27	4,278	98	1,020	146	
93209	COL201904-63	2019	throat swab	1,238	2,539,864	128	78,589	2,052	18,231	39	624	221	306	499	
93210	COL201904-65	2019	throat swab	1,093	2,385,065	128	76,381	2,183	16,526	43	1,391	199	334	404	
93212	COL201904-83	2019	throat swab	318	2,134,577	128	120,840	6,713	24,755	26	6,134	85	1,970	110	
93213	COL201905-33	2019	throat swab	285	2,123,568	128	115,923	7,452	32,990	22	8,784	68	1,922	90	
189701	COL201802-76	2018	urethral swab	1	2,205,116										
